# Peer Support for Type 2 Diabetes Management in Low- and Middle-Income Countries (LMICs): A Scoping Review

**DOI:** 10.5334/gh.1299

**Published:** 2024-02-20

**Authors:** Diana Sherifali, Lilian Pinto da Silva, Pooja Dewan, F. Aaysha Cader, Zainab Dakhil, Bishal Gyawali, Sheila Klassen, Israa Fadhil Yaseen, Milos Jovkovic, Saira Khalid, Donna Fitzpatrick-Lewis, Paige Alliston, Megan Racey

**Affiliations:** 1 McMaster Evidence Review and Synthesis Team, School of Nursing, Faculty of Health Sciences, McMaster University, Hamilton, Ontario, Canada; 2 Faculty of Physical Therapy, Federal University of Juiz de Fora, Juiz de Fora, Minas Gerais, Brazil; 3 British Heart Foundation Cardiovascular Research Centre, University of Glasgow, Scotland, UK; 4 Ibrahim Cardiac Hospital & Research Institute, Dhaka, Bangladesh; 5 Ibn Al-Bitar Cardiac Centre, Al-Kindy College of Medicine/University of Baghdad, Baghdad, Iraq; 6 Global Health Section, Department of Public Health, University of Copenhagen, Copenhagen, Denmark; 7 Department of Global Health and Social Medicine, Harvard Medical School, Boston, Massachusetts, United States of America; 8 Baghdad Heart Center, Baghdad Teaching Hospital, Medical City, Baghdad, Iraq

**Keywords:** Type 2 diabetes, Cardiometabolic, Peer support, Low and middle income, Scoping review

## Abstract

**Background:**

Although there is evidence of peer support in high-income countries, the use of peer support as an intervention for cardiometabolic disease management, including type 2 diabetes (T2DM), in low- and middle-income countries (LMICs), is unclear.

**Methods:**

A scoping review methodology was used to search the databases MEDLINE, Embase, Emcare, PsycINFO, LILACS, CDSR, and CENTRAL.

**Results:**

Twenty-eight studies were included in this scoping review. Of these, 67% were developed in Asia, 22% in Africa, and 11% in the Americas. The definition of peer support varied; however, peer support offered a social and emotional dimension to help individuals cope with negative emotions and barriers while promoting disease management.

**Conclusions:**

Findings from this scopingreview highlight a lack of consistency in defining peer support as a component of CMD management in LMICs. A clear definition of peer support and ongoing program evaluation is recommended for future research.

## Introduction

Cardiometabolic diseases (CMDs) constitute five of the top ten causes of death globally [1]. These primarily include cardiovascular diseases (CVDs) and Type 2 Diabetes Mellitus (T2DM), which share common risk factors such as obesity, hypertension, unhealthy diet, tobacco use, air pollution, and physical inactivity [1]. Patients with T2DM are often required to follow complex medication regimens, modification of lifestyle factors, and adhere to screening and monitoring of complications, all of which involve a commitment on the part of both patients and their caregivers [2,3].

The majority of everyday care for CMDs, such as T2DM, is handled by patients and/or their families, requiring the significant need for self-management of their chronic disease as well as psychosocial and medical support. A peer support intervention could provide a viable solution for empowering people to manage their health, thereby reducing the burden on informal caregivers and healthcare systems. In general, peer support refers to a process through which people who share common experiences or face similar challenges come together as equals to give and receive health based on the knowledge that comes through shared experience [4]. In the health care context, peer support provides emotional, appraisal, informational, and instrumental support through non-hierarchical and often reciprocal relationships by a non-professional who shares a lived experience with those they help [5,6]. Studies have shown that different forms of peer support can help to improve self-management behaviours, including adherence to prescribed medications, following diet and exercise regimens, as well as blood glucose monitoring [6,7,8]. Peer support can also provide human connections, by ‘being there’ through their shared lived experiences, social and emotional support through empathetic listening and encouragement. Additionally, peer support may assist with health system navigation or linkages to clinical care, as well as proactive, flexible, and continual long-term follow-up, particularly in situations where healthcare resources are limited [5,6,9]. In recent years, peer support programs have been widely used to improve chronic disease management, alcohol and substance use as well as breastfeeding outcomes [10].

Type 2 diabetes mellitus is associated with a marked increase in the risk of macrovascular disorders, including CVDs [11,12,13]. Therefore, it is of critical importance to minimize this risk by carefully managing modifiable CVD risk factors in patients with T2DM. Treatment and management strategies are multi-faceted and include pharmacological and lifestyle interventions to reduce elevated blood pressure and blood glucose levels, dyslipidemia, obesity, cigarette smoking, physical inactivity, and prothrombotic factors [12,13] in addition to the proper selection of antidiabetic agents that are associated with the primary prevention of CVD such as heart failure [14]. Globally, less than one-third of patients with T2DM and CVDs receive optimal secondary preventive measures such as lipid and blood pressure control, despite evidence that consistent control of these CVD risk factors leads to lower cardiovascular events, as evidenced by the global Trial Evaluating Cardiovascular Outcomes with Sitagliptin (TECOS) study [15]. This underlines the critical need for primary and secondary prevention programs for CVDs in individuals with diabetes.

Of the CMDs, an estimated 80% of the global population with T2DM resides in low- and middle-income countries (LMICs) [16]. Even more problematic, the incidence of all CMDs in the LMICs is on the rise due to changing risk factor profiles, including genetic, environmental (such as increasing urbanization) and behavioural (such as poor nutrition and lack of physical activities) factors [17]. Although there is some evidence of peer support for CMDs in high-income countries [18], there is a paucity of data on peer support in LMIC, where its burden is greatest. Furthermore, the specific definition and extent of peer support for T2DM and other CMDs in LMICs are not well-known.

Accordingly, this scoping review aims to understand the definitions, models of peer support, and experiences with peer support in patients with CMDs with a focus on T2DM in LMICs. A scoping review will facilitate the synthesis of various research paradigms, methodological approaches (e.g., randomized control trials and before/after studies), and patient or caregiver-reported outcomes and/or experiences. This synthesis methodology is chosen as it supports the mapping of how research is conducted in a certain area or field, clarifying concepts and characterizations in the literature, identifying key factors/issues related to a concept, analyzing and identifying knowledge gaps, examining how research is undertaken in a particular field and as a precursor to a systematic review [19].

## Methods

Considering the promising role of peer support in diabetes and as an initiative by the World Heart Federation (WHF) to decrease CVD burden in diabetes, on October 2021, the authors (healthcare providers from five continents; except MR, MJ, SK, PA, and DFL) came together through the World Heart Federation Emerging Leaders Program in Lisbon/Portugal to work on peer support in Diabetes. This scoping review was guided by the methodologies of Arksey and O’Malley [20], Munn, Peters [21], and Pollock, Davies [22] and adheres to the Preferred Reporting Items for Systematic Reviews and Meta-Analyses (PRISMA) extension for scoping reviews [23].

### Search strategy

The search terms (e.g. cardiometabolic, diabetes, peer support, low middle-income), databases, and strategy were developed in consultation with a research librarian at McMaster University (Supplemental File 1). MEDLINE, Embase, Emcare, PsycINFO, Latin American and Caribbean Health Sciences Literature (LILACS), Cochrane Database of Systematic Reviews (CDSR), and Cochrane Central Register of Controlled Trials (CENTRAL) databases were searched from inception to March 28, 2022. Furthermore, we manually searched reference lists of relevant reviews and included studies for citations that were not captured in our search. We also conducted our search to include grey literature via Google Scholar. Results from the search were deduplicated, and citations were uploaded to a secure internet-based platform for screening (DistillerSR, Evidence Partners Inc., Ottawa, Canada).

### Inclusion and exclusion criteria

We included studies conducted in LMICs according to the Organisation for Economic Co-operation and Development (OECD) [24], with peer support for people living with T2DM or at risk for CMDs which included diabetes, coronary artery disease, stroke, hypertension, disorders of the vascular system and obesity [2,25,26]. If not all, at least part of the study participants needed to have one of the following conditions: (1) T2DM (as defined by the WHO), [27] and (2) be at risk or living with another CMD. For our review, peer support could be delivered through any mode (face-to-face, virtual, phone, or web) and individual or group-based, but had to be delivered by lay individuals which are not part of the country’s healthcare system. According to Dennis (2003), lay individuals can have varying degrees of training and can be selected by community members, health professionals, program developers, or self-selected [5]. Interventions led or facilitated by a professional (or non-peer) were included, provided that the focus of the intervention was to provide peer-to-peer interaction. There was no language filter, but the studies included were all peer-reviewed, and comprised of quantitative study designs. No control group or minimal contact was required. We did not include or exclude studies based on the outcomes measured. Studies were excluded if they were not conducted in an LMIC, if the population did not have CMDs, or if the peer support was part of a multi-component/complex intervention where the effects of the peer support element could not be isolated. Letters, commentaries, editorials, conference abstracts, and doctoral theses were also excluded.

### Data extraction and charting

A team of researchers conducted the screening and data extraction (MR, MJ, SK, DS, DFL). A minimum of two reviewers were required to independently and in duplicate screen titles and abstracts of all potentially eligible studies. Articles marked for inclusion by either team member went on to full-text screening which was completed independently and in duplicate by two team members and required consensus for inclusion or exclusion. After confirmation of the included studies, we looked for related publications that met our search dates and inclusion criteria and grouped multiple publications that were based on the same study/intervention. We developed, piloted, and deployed standardized forms for data extraction which were housed in a web-based systematic review software program. All authors provided feedback and approved the components of these forms. Two team members independently verified all extracted data and disagreements were resolved through discussion and/or third-party consultation. Conflicts were resolved by the lead researcher of this review (MR). For each primary study, one team member extracted study characteristics (including the study aim, sample size, methods, peer support definition and mode of delivery, population demographics, study outcome types and time points, study length, location, and setting), and the 12 components of the template for intervention description and replication (TIDieR) checklist and guide [28]. Outcomes from each study were mapped to the five key functions of peer support as defined by Evans, Daaleman [6,9]. The five key functions of peer support are as follows:

1. Being there: Providing support through shared experiences.
2. Assistance in daily management: Peer supporters can use their own experiences with diet, physical activity and medication adherence to help people manage diabetes in their daily lives and provide key resources, such as where to buy healthy foods.
3. Social and emotional support: Through empathetic listening and encouragement, peers help patients to cope with social or emotional barriers and to stay motivated to reach their goals.
4. Linkages to clinical care and community resources: Peer supporters can help bridge the gap between patients and health professionals and encourage individuals to seek out clinical and community resources when it is appropriate.
5. Ongoing support: Peer supporters successfully keep patients engaged by providing proactive, flexible, and continual long-term follow-up.

## Results

**Figure 1:**
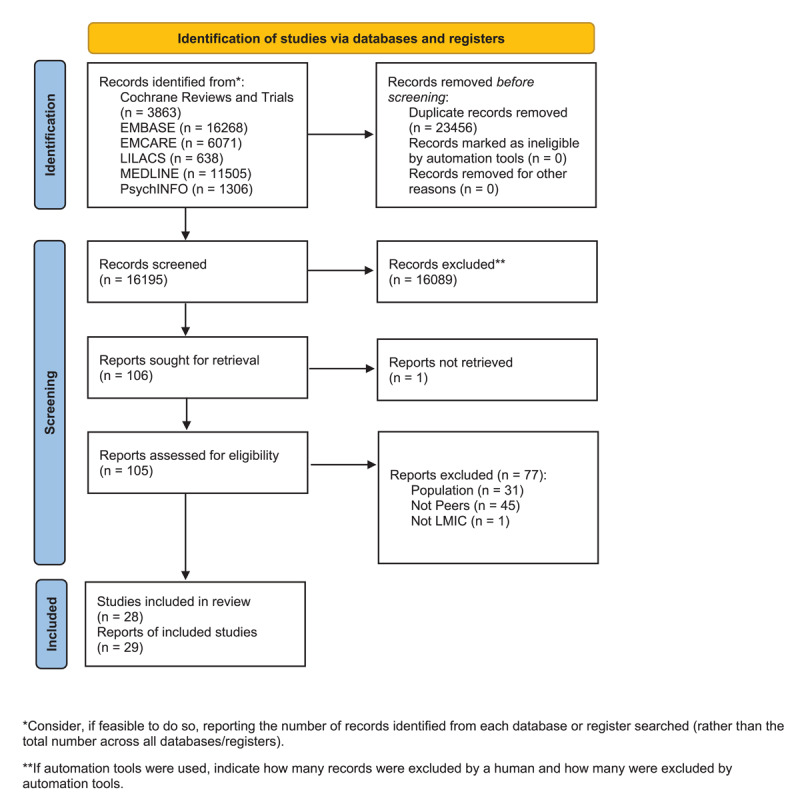
PRISMA Flowchart. Page MJ, McKenzie JE, Bossuyt PM, Boutron I, Hoffmann TC, Mulrow CD, et al. The PRISMA 2020 statement: an updated guideline for reporting systematic reviews. BMJ 2021;372:n71. doi: 10.1136/bmj.n71.

Our search yielded 16,195 citations after duplicates were removed. We assessed 105 full-text citations for eligibility and excluded 77 of these studies, mostly due to the reason that they were not the correct population or did not meet our definition of peers. The remaining 28 studies [8,29,30,31,32,33,34,35,36,37,38,39,40,41,42,43,44,45,46,47,48,49,50,51,52,53,54,55] described in 29 publications [56] were included in a narrative synthesis for this scoping review (Figure 1). The included studies were predominantly randomized controlled trials (RCTs) or controlled clinical trials (CCTs) (n = 20) (Table 1). Other study designs included observational studies (n = 5), one group pre/post studies (n = 2) and a single systematic review (n = 1).

**Table 1:** Characteristics of included studies; by study design.


INTERVENTION STUDIES (n = 22)						

AUTHOR, YEAR	STUDY DESIGN	SAMPLE SIZE^1^	CLINICAL CONDITION TO STUDY INCLUSION	LENGTH OF STUDY	MODE OF PEER SUPPORT	PRIMARY OUTCOME(S)^2^

Ahmadi, 2018 [34]	RCT	120	T2DM	12 weeks	Face to face	Self-Care Behaviours

Assah, 2015 [42]	CCT	192	T2DM	6 months	Face to face, telephone	HbA1c

Baumann, 2015 [39]	One group, pre/post design	60	T2DM	4 months	Face to face, telephone	Diabetes Self-Care Behaviours, BMI, HbA1c

Castillo-Hernandez, 2021 [30]	RCT	58	T2DM	8 months	Face to face	HbA1c, Diabetes-related Quality of Life

Chan, 2014# [53]	RCT	628	T2DM	12 months	Face to face, telephone	HbA1c

Debussche, 2018 [8]	RCT	151	T2DM	12 months	Face to face	HbA1c

Gagliardino, 2013 [55]	RCT	198	T2DM	13 months	Face to face, telephone	HbA1c

Ghasemi, 2019 [33]	RCT	56	Diabetes	8 sessions	Face to face	Diabetes Quality of Life

Ju, 2018 [43]	RCT	400	T2DM	12 months	Face to face, phone, e-mail	Diabetes Distress

Khan, 2018 [54]	CCT	133	T2DM	12 weeks	Face to face	Knowledge, Lifestyle changes, HbA1c, Fasting blood glucose, Weight, BMI, Blood pressure

Khetan, 2019 [41]	RCT	1242	≥ 1 cardiovascular risk factor (Diabetes, hypertension, or current daily smoker)	2 years	Face to face	Systolic blood pressure, fasting blood glucose, average number of cigarettes/bidis smoked

Latina, 2020 [40]	RCT	402	≥ 2 cardiovascular risk factors (Elevated BP, overweight/obese, elevated blood glucose, low physical activity, low fruit and vegetable intake, hyperglycaemia, dyslipidemia or current smoking)	12 months	Face to face	Cardiovascular Health

Paz-Pacheco, 2017 [36]	RCT	155	T2DM	6 months	Face to face	Anthropometric, biochemical characteristics, health behaviour measures

Peimani, 2018 [35]	RCT	200	T2DM	6 months	Face to face, telephone	HbA1c, BMI, Diabetes Self-Management and Self-Efficacy, Health Related Quality of Life

Rotheram-Borus, 2012 [45]	One group, pre/post design	22	T2DM, T1DM (1 participant)	6 months	Face to face, telephone	Process measures, Diabetes-specific measures (BMI, blood pressure, emotional distress, and styles of coping), steps taken, and hours slept

Sazlina, 2015 [50]	RCT	69	T2DM	12 weeks	Face to face, telephone	Physical Activity

Shahsavari, 2021 [46]	RCT	80	T2DM	3 months	Face to face, telephone	Diabetes Quality of Life

Sreedevi, 2017 [37]	RCT	124	T2DM	3 months	Face to face, telephone	Fasting plasma glucose, HbA1c, Quality of Life, Medication adherence

Thuita, 2020 [31]	RCT	153	T2DM	8 weeks	Face to face	Metabolic Syndrome

Yin, 2015# [44]	CCT	13	T2DM	4 years	Telephone	HbA1c

Zeng, 2016 [49]	CCT	325	Diabetes or Hypertension	6 months	Face to face	Depression, Anxiety, and Quality of life

Zhong, 2015 [38]	RCT	726	T2DM	6 months	Face to face	Implementation (Acceptability, Implementation, Reach, Recruitment), Diabetes knowledge, Self-management practices, Attitudes toward self-management, self-efficacy, and social support

**OBSERVATIONAL STUDIES (n = 5)**						

**AUTHOR, YEAR**	**STUDY DESIGN**	**SAMPLE SIZE^1^**	**DISEASES AT BASELINE**	**LENGTH OF STUDY**	**MODE OF PEER SUPPORT**	**PRIMARY OUTCOME^2^**

Hernandez, 2021$ [52]	Retrospective exploratory analysis	6677	Diabetes	2.3 years	Face to face	Blood pressure

Liu, 2020 [51]	Cohort	1284	Diabetes	12 months	Face to face, telephone	HbA1c

Mwakalinga, 2021 [29]	Cross-sectional	176	Diabetes	4 years	Face to face	Diabetes Knowledge, Attitude, and Empowerment

Rao, 2020$ [47]	Cohort	4210	Diabetes	3.4 years	Face to face	Blood glucose, blood pressure, weight, HbA1c, lipid panel, urine protein, Medication adherence

Taniguchi, 2017$ [48]	Cohort	2230	Diabetes	24 months	Face to face	Fasting blood glucose, blood pressure

**REVIEW (n = 1)**						

**AUTHOR, YEAR**	**STUDY DESIGN**	**# OF STUDIES**	**AIM OF REVIEW**	**INCLUSION CRITERIA**	**EXCLUSION CRITERIA**	**MAIN FINDING**

Werfalli, 2020 [32]	Systematic review	11	Explores the nature of Community-based peer and community health worker-led diabetes self-management programs in low- and middle-income countries’ primary care settings and evaluates implementation strategies and diabetes-related health outcomes	RCTs, non-randomised controlled trials, and quasi-randomised controlled trials. Participants ≥ 18 years of age and have either type 1 or T2D, but not gestational diabetes nor diabetes due to other causes. Studies that reported contact with an individual or a group of peers with a minimum follow up period of 3 months. Studies that reported at least one behavioural, psychological, or clinical measure. English studies between the years 2000–2019	Peer support that was exclusively telephone- and web-based. Peer support was part of a multicomponent/complex intervention, where the effects of the peer support element could not be isolated	Community-based peer and community health worker-led diabetes self-management programs were inconsistently associated with improvements in clinical, behavioural, and psychological outcomes. Limited and low level of evidence for benefit from peer support in diabetes care outcomes


RCT: Randomized controlled trial; CCT: Controlled clinical trial; N/A: not applicable; N/R: not reported; CVD: Cardiovascular disease; T2DM: Type 2 Diabetes Mellitus; HbA1c: Hemoglobin A1c or glycated hemoglobin; BMI: body mass index; CHW: Community health worker (A peer can be interpreted in different ways. In this study, a peer refers to a community health worker (CHW) working and staying in the same area as the patients he/she serves). 1. Full study sample size enrolled in study; 2. Primary outcome as defined by study authors. Some studies had multiple primary outcomes as they were pilot or feasibility studies. Details on the measurement tool used can be found in Supplemental File 2. # Related publications (n = 2). $ Related publications (n = 3).

### Study characteristics and reporting of studies

**Figure 2:**
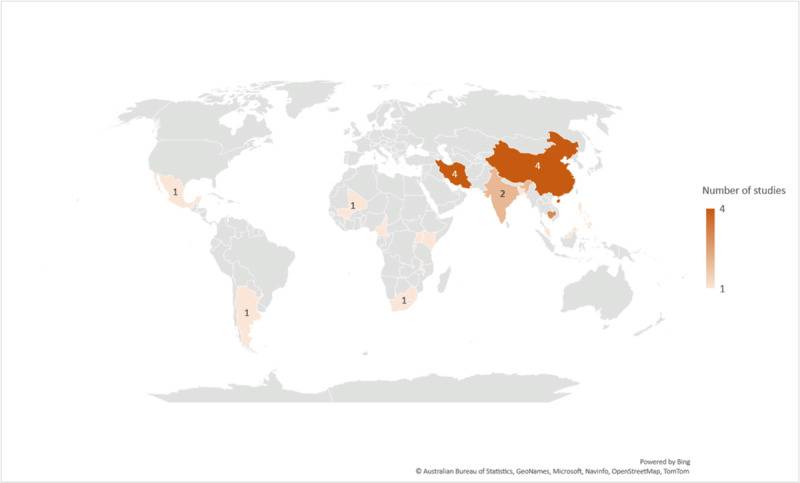
Number of included papers, by country (n = 27). * *Note*: One systematic review is not included in this figure. Additional details on continent, countries, and number of studies: North America – 2 countries (Mexico and Grenada) total 2 studies; South America – 1 country (Argentina) total 1 study; Africa – 6 countries (Uganda, South Africa, Mali, Kenya, Malawi, and Cameroon) total 6 studies; Asia – 8 countries (Iran, Philippines, Malaysia, India, Hong Kong, China, Cambodia, and Bangladesh) total 18 studies.

Characteristics of the primary papers can be found in Table 1 and the full characteristics of the individual primary papers can be found in Supplemental File 2. The included studies from 17 LMICs were published between 2012 and 2021 with 64% (n = 18) having been published in the last five years (Figure 2). There were four studies from China, four studies from Iran, three studies from Cambodia, two studies each from India and Hong Kong, and one study each from Malaysia and South Africa. The remaining 10 countries were each represented by one included study in our review. Intervention studies ranged from 13 participants to over 6,000 and from four weeks to two years in duration.

### Characteristics of peer support interventions

**Figure 3:**
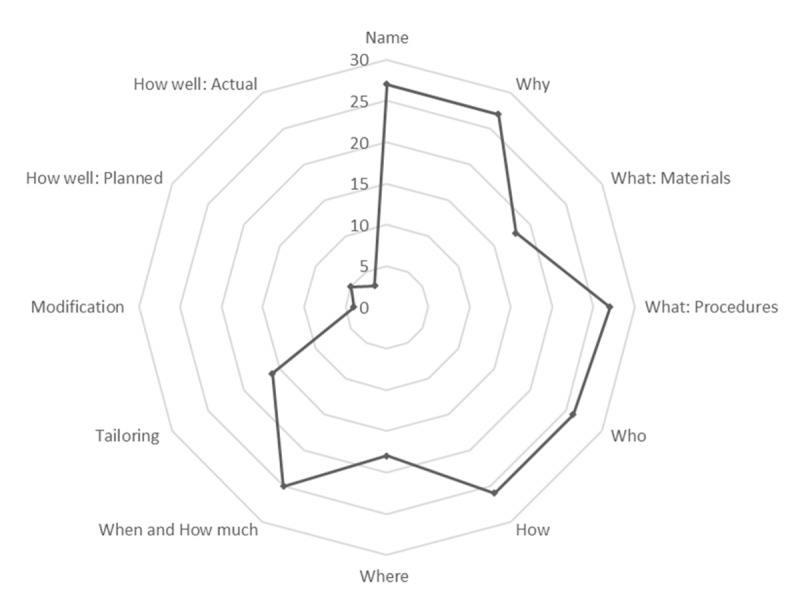
Spider chart of the total reporting of each component from the modified TIDieR checklist. The spider chart illustrates that the components of the TIDieR checklist that were most commonly reported among the group of included studies were: name, why, what: procedures, who, how, when and how much. * *Note*: One systematic review is not included in this figure.

We assessed the TIDieR checklist for the 27 intervention and observational studies in our review. We did not complete the checklist for the systematic review as that is beyond the purpose of the TIDieR tool. Depending on the nature of the study and its design, some components were not applicable, rather than not reported (see Supplemental File 3 for more details). Of the 12 components from the TIDieR checklist, all the included studies reported on the study name, study objective, and procedures or methods (Figure 3). Other common items were who provided (n = 26), how (n = 26), when and how much (n = 25), where (n = 18), materials (n = 18), and tailoring (n = 16). Many of these components are related to standard reporting guidelines required and adhered to by peer-reviewed journals. In addition, due to the nature of peer support studies, the included studies were designed to be tailored and personalized to the participants. Some studies did not report on any level of personalization, or this component was not applicable as the peer support was group-based and generic. Very few studies reported on any study modifications (n = 4) and how well the intervention was delivered, either planned (n = 5) or actual (n = 3) measures of adherence. It is important to note that fidelity goes beyond participant attendance measures and was specific to reporting in the study of fidelity and adherence to delivery as planned.

Table 2 provides details regarding the definition of peers and how the peers interacted with the participants (mode of peer support). The definition and terminology of peers varied depending on the study but due to our inclusion criteria, none of the peers were healthcare professionals or meant to act in place of healthcare professionals and the services they provide. In general, the majority of peers were those living in the community, with similar experiences, who had good control of their diseases and/or risk factors (if applicable) and adequate communication skills. However, not all peers had lived experiences of diabetes or other CMD risk factors. In such cases, these individuals were often chosen due to their status or role as community champions or leaders. Many of the peers were trained for study components, or it was otherwise unclear but included positions that were paid, volunteered, or received other honorariums for their time. All but one study included face-to-face interaction between the peers and the participants. The study which did not use in-person interaction instead chose phone calls, and another 11 studies incorporated phone calls in addition to face-to-face peer interaction. One study also used e-mail correspondence between peers and participants. Further details regarding when, where, and how the peers interacted with the participants and their role in the study can be found in Supplemental Files 2 and 3.

**Table 2:** Details of peer support and providers.


INTERVENTION STUDIES (n = 22)				

AUTHOR, YEAR	COUNTRY	DEFINITION OF PEER SUPPORT	SETTING	PROVIDERS

Ahmadi, 2018 [34]	Iran	Peer education is an information exchange of attitudes and behaviours from individuals who are not specialists but have similar experiences.	Diabetes Clinic at a university medical center hospital.	A peer was a known diabetic with good control of blood glucose, few complications, able to manage sessions, had personal interest to collaborate and provide support, had good social communication skills (e.g., good appearance, tone of voice, eye contact) and education higher than middle school. Peers underwent 2 weeks training from the first author.

Assah, 2015 [42]	Cameroon	Peer-support care models provide a low-cost, flexible means to supplement formal healthcare support for chronic diseases.	Out of the hospital setting	A peer supporter was recruited for each of the 10 groups of the intervention arm based on their past history and clinical profile: better glycaemic and metabolic control than their peers, more compliant with their clinic visits, and more experiential knowledge on diabetes. They underwent a two-day training workshop.

Baumann, 2015 [39]	Uganda	Peer support is a promising approach toward achieving self-care goals.	Diabetes clinic	Peer champions were patients able to read and speak English who received additional training in communication skills to provide peer partners emotional support and assistance with daily management.

Castillo-Hernandez, 2021 [30]	Mexico	Provision of support from an individual member of the community, with experiential knowledge based on sharing similar life experiences.	Community centre	All peer leaders (supporters) were known diabetics with most recent HbA1C <8%. They were required to have good communication skills and were trained on basic aspects of diabetes, communication and leadership skills according to the Peer Leader Manual.

Chan, 2014# [53]	Hong Kong	Provision of support for daily management, linkage to clinical care, and ongoing social and emotional support.	3 Diabetes centres	Peer supporters were motivated patients with well-controlled T2DM who received 32 hours of training to become peer supporters. They were reinforced on the principles of communication and empathic listening and encouraged to share their positive experiences to assist their peers to manage diabetes on a day-to-day basis. Additionally, they were reminded of factors that could influence blood glucose level, eg, diet, exercise, poor sleep, stress, changes in daily routines, bodyweight, medications, and concurrent illnesses, and thus the importance of self-monitoring of blood glucose. Some of them were active members of patient groups organized by lay associations or diabetes centres.

Debussche, 2018 [8]	Mali	N/R	Community setting	Peer educators (PEs) were recruited from the local association of diabetic patients. The following criteria were used for selection – having diabetes, living in the locality, undergoing regular checks with a referent physician, volunteering to deliver educational sessions, and being fluent in both French and the local Bambara language. The recruited PEs attended an initial 4-day training program. They underwent two further evaluations before being actively involved in the project.

Gagliardino, 2013 [55]	Argentina	A reasonable approach involving people with diabetes in the delivery of education and support needed for long-term self-management.	Houssay Center in La Plata	Peers were recruited on the basis of their excellent diabetes control, self-motivation, communication and support skills and interest. They were trained for 3 days using the curriculum of the health professionals Training Course on Diabetes Education.

Ghasemi, 2019 [33]	Iran	Peer education is a process in which motivated and trained individuals are responsible for education of their peers that aims to raise awareness and improve skills in the individuals and enable them to accept their responsibility in protecting their health	Two health centres	Recruitment and details of peers was not described. The authors describe the peers to be interested and highly motivated. Peers who were training their group were further supervised by the researcher.

Ju, 2018 [43]	China	The provision of support (through activities) from an individual with experiential knowledge based on sharing of similar life experiences	Eight community health centres	Peer leaders were chosen based on residence, demographics and other characteristics, including interpersonal skills evident in interviews, time available and willingness to cooperate as part of a team and follow study protocols. Peer leaders guided participants to carry out activities with the help of community health centres or medical volunteers.

Khan, 2018 [54]	Bangladesh	People with diabetes educating other patients regarding diabetes	Outpatient Department (OPD) of BIRDEM (the Tertiary Hospital of Diabetic Association of Bangladesh)	Peer educators were diabetics for at least 5 years with the following characteristics – age>40yrs, HbA1C <7%, graduates, committed to training and willing to spend sufficient time, enthusiastic to be peer educators and residing in Dhaka city. They underwent a three-day training program with pre and post-training assessments.

Khetan, 2019 [41]	India	N/R	Community	Community health workers (CHWs) were recruited for the study and were not previously a part of the health system. The inclusion criteria do become a CHW included being a female resident of the study area for at least the past two years, between 18 and 45 years of age, having a tenth-grade level of education and possessing spoken and written knowledge of the local language. They received staggered training focused on hypertension, followed by diabetes, and then smoking. Training for each risk factor was delivered over 1 to 2 weeks (3 h/day). All CHWs were retained from the start to the end of the intervention, with zero attrition.

Latina, 2020 [40]	Grenada	Peer support is a low-cost intervention and therefore decrease CV risk.	5 Local parishes	A Peer leader was a motivated community lay-person. They underwent an additional three-hour training session on leadership and communication skills in addition to the relevant healthy behaviour promotion.

Paz-Pacheco, 2017 [36]	Philippines	‘Community catalysts,’ to promote a healthy lifestyle among people with diabetes in the community	Village health centres	Volunteer peer educators were recruited among the participants (known diabetics). They attended a two-day workshop during which they received a course manual that described both the course content and process on how to teach them.

Peimani, 2018 [35]	Iran	Peer support programs are a promising way to boost social and emotional support, help patients in day-to-day management of living with diabetes and promote linkages to clinical care	University specialty clinic.	Peers who were known diabetics were nominated by physicians and diabetes educator nurses in the clinic based on their diabetes control (HbA1C <8%), good interpersonal skills, self motivation and good active and non-judgemental listening skills. The peers were also to be able to read and write and had to attend a three-day course.

Rotheram-Borus, 2012 [45]	South Africa	N/R	Xhosa township	Peer mentors were positive role models who had lost weight and increased exercise after their T2DM diagnosis. They were trained in the management of diabetes, support processes and group management by the project team.

Sazlina, 2015 [50]	Malaysia	Assistance in applying disease management and prevention plans in daily life, emotional and social support, linkage to clinical care and on-going support.	Primary healthcare clinic.	Peer mentors were volunteers with ≥5 years of T2DM, engaged in regular physical activity, had HbA1c <8% and living in the community of the study location. Peer mentors also attended a 2-day training session.

Shahsavari, 2021 [46]	Iran	Peer support is delivered by similar people with diabetes in social and emotional contexts to improve patients’ relationships with clinical caregivers and help them manage their daily activities of a life with diabetes.	Public spaces (mosques, coffee shops or restaurants)	Peers were those diagnosed with T2DM for at least 1 year (latest HbA1c <8%), having at least a high school diploma, had basic knowledge about diabetes, had no diabetes related chronic complications, attending all peer education sessions and being approved for their communication and interpersonal skills in the face-to-face interview session by the research team.

Sreedevi, 2017 [37]	India	Peer support was defined as support from an individual with experiential knowledge based on a sharing of similar life experiences or prevention plans in daily life	Rural health training centre.	Peer mentors (PMs) were identified from the community. Eligibility criteria included having T2DM for at least 1 years with a random plasma glucose (RPG) <=250mg/dl in the last reading, someone who was generally adherent to treatment and behaviour change regimen as judged by the investigation team. Had capacity and commitment to undergo the training required, an understanding of patient confidentiality and undertaking to liaise with the concerned doctor if unanticipated problems arose during the course of the study. Peer mentors underwent a two-day training program.

Thuita, 2020 [31]	Kenya	Peer to peer social and emotional support has been shown to help people apply disease management or prevention plans in daily life, and links individuals with clinical, community, and other resources.	Thika Level 5 Hospital.	The Nutrition education with Peer-to-Peer support (NEP) group were given peer-to-peer support training in addition to the nutrition training program. Members of the peer support group were encouraged to set and share with one another other weekly goals for specific changes in their eating and physical activity behaviour. A trained peer educator living with diabetes for 13 years joined the PI during monthly meetings and encouraged participants in the peer support groups by sharing his experience.

Yin, 2015# [44]	Hong Kong	Peer support refers to the transfer of experiential knowledge of a specific behavior or coping strategy for a stressor between people who share a particular characteristic.	Managed in the usual care setting of their hospital or community-based clinic.	Peers were those living with T2DM, aged 18 to 75 years with HbA1C <8%, had a good understanding of living with diabetes, clear communication skills. They underwent the ‘train-the-trainer’ program.

Zeng, 2016 [49]	China	N/R	Community health centres	Peers were community volunteers who had received guidance from counselors. Meetings by the peers focused on (a) the management of chronic diseases, (b) healthy lifestyles, (c) psychological coping skills for dealing with diabetes and hypertension, (d) knowledge about depression and anxiety, and (e) self-awareness of negative emotions. The meetings also provided emotional and social support to the participants.

Zhong, 2015 [38]	China	Assistance in daily management, social and emotional support, linkage to clinical care and community resources, and ongoing availability of support.	Community Health Service Centers and participants homes.	Peer leaders were those diagnosed with T2DM for more than 1 year, willing to volunteer and generally adhered to both medication and behavioral management regimens. Additional criteria were altruism, positive and sociable personality, availability of time, an understanding of the importance of patient confidentiality, good relationships with community residents and leadership in their communities. They underwent 3 days’ training. Training emphasized the key functions of peer support promoted by Peers for Progress. Peer leaders were retired adults who had diabetes for a mean of 9.3 years.

**OBSERVATIONAL STUDIES (n = 5)**				

**AUTHOR, YEAR**	**COUNTRY**	**DEFINITION OF PEER SUPPORT**	**SETTING**	**PROVIDERS**

Hernandez, 2021$ [52]	Cambodia	N/R	N/R	Peer Educators (PEs) were patients with diabetes and/or hypertension selected for their motivation who screened and initiated management of community members in their local villages, Training of PEs was undertaken by MoPoTsyo (Cambodian NGO).

Liu, 2020 [51]	China	N/R	Community Health Centers	Majority of the peer leaders (PLs) were individuals living with diabetes who were recruited based on existing relationships with people in the community and trained with knowledge and skills to help patients make the transition from discussing problems to taking action using a ‘Diabetes Action Plan’ as a framework.

Mwakalinga, 2021 [29]	Malawi	N/R	Kamuzu Central Hospital	Study did not specify characteristics of the peers. They were trained using support material developed by the Peers for Progress organization.

Rao, 2020$ [47]	Cambodia	Peer educator programs have been used to improve chronic disease management by providing educational support and linkages to care, particularly in resource-poor settings.	Peer educator homes	Peer educators (PEs) were community members with diabetes selected based on literacy, motivation and social aptitude. They underwent six-week training course developed by physicians, pharmacists, and experienced peer educators.

Taniguchi, 2017$ [48]	Cambodia	Peer support programs utilize peer educators who are non-professionals to provide a variety of functions, including social and emotional support, assistance with disease management, and linkage to clinical care and community resources aiming to engage patients in self-management of their disease to sustain behaviours needed to manage diabetes and decrease the risk of diabetes complications.	Typically, peer educator homes	Peer educators (PEs) were people with diabetes and were selected on their ability to read and write and their willingness to commit to fulfill the role. They received 6 weeks training.


N/A: not applicable; N/R: not reported; # Related publications (n = 2). $ Related publications (n = 3).

### Peer support impact on outcomes

**Figure 4:**
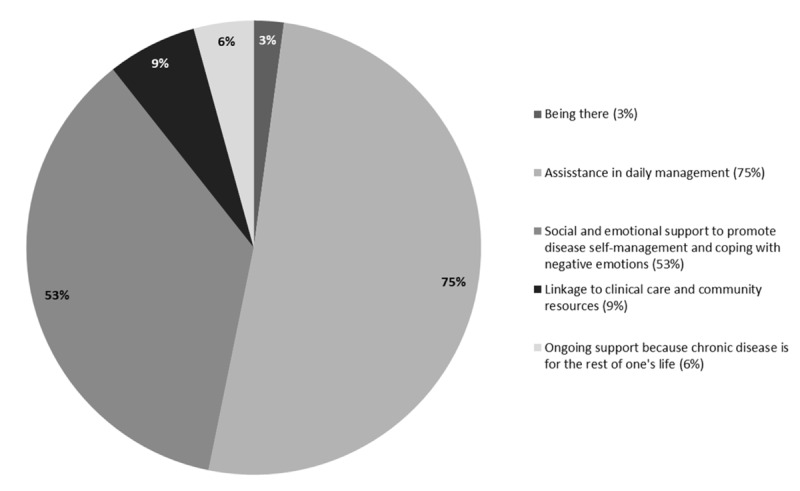
Mapping of outcomes from included studies to the five key functions of peer support as defined by Evans et. al (number of papers = 27; studies could be mapped to more than one function of peer support).

Outcomes from each included study were mapped to the five key functions of peer support as defined by Evans, Daaleman (Figure 4) [6]. Primary outcomes of each study can be found in Table 1 and all outcomes for the included studies can be found in Supplemental File 2, including how these outcomes mapped to the five key functions of peer support. It is important to note that some studies had multiple primary outcomes as they were pilot or feasibility studies, and not aimed or powered to assess effectiveness based on a primary outcome. Two of the studies included in our review directly referenced the key functions of peer support in their methods and/or results [10,38,39]. The majority of studies (75%; n = 24) had outcomes that related to assistance in daily management. This function of peer support aligned with outcomes such as self-care behaviours, anthropometric measurements, glucose control, knowledge, and dietary practices. The second common key function of peer support was social and emotional support to promote disease self-management and coping with negative emotions and barriers (53%; n = 17). This function of peer support included outcomes such as quality of life, empowerment, cognitive and psychological behavioural questionnaires, and scales related to anxiety, distress and depression, and well-being surveys. Being there (n = 1), linkage to clinical care and community resources (n = 3), and ongoing support (n = 2) were rarely captured by outcomes in our included studies. This may be due to the ambiguity of the ‘being there’ function, which is hard to measure as well as the lack of long-term follow-up in the included studies which would aid in the ‘ongoing support’ function.

## Discussion

This review addresses a gap that currently exists in the literature surrounding the definition and models of peer support for T2DM management in LMICs and provides context for the ways in which peer support can differ within LMICs. This scoping review revealed that, currently, only 12% of the 142 countries classified as low-and-middle-income countries, according to the OECD, have developed at least one study on peer support for T2D and cardiovascular disease prevention and management. Sixty-seven percent of the 27 primary studies included in this review were developed in Asia, 22% in Africa, and 11% in the Americas. This finding demonstrates the need to adopt other ways to understand the definitions, models of peer support, and experiences with peer support for people living with or at risk for cardiometabolic diseases in low-and-middle-income countries. The influence of factors such as limited access to healthcare providers, financial and geographical barriers, mistrust in the Western Medical system in many rural LMIC areas, fear of accessing medical care, or a lack of education regarding the need to access care should be explored to better understand peer support interventions in LMICs. The authors of this review are exploring the role and implementation of peer support through international surveys, with particular attention to LMICs.

According to the International Diabetes Federation, the top 10 countries for the number of adults between 20 and 79 years living with diabetes in 2021 and 2045 include China, India, Pakistan, Indonesia, Brazil, Mexico, Bangladesh, Egypt, and Turkey [57]. Since our search did not find studies from Pakistan, Brazil, Egypt, and Turkey, concerns were brought to light in finding ways to know what is being done in these countries regarding peer support for people living with T2DM.

This scoping review found a lack of consistency in the definition of peer support across included studies. Additionally, there was no mention of the peer support definition in 26% of the primary studies, which limits the analysis of the complete peer support definition understanding focusing on T2D management in LMICs. All studies reported a description of the role of the peer supporter. Regarding the five key functions of peer support as defined by Evans et al. [6], assistance in daily management was the function of peer support most often covered in the studies. Different terms were used to define peers, including a champion [39], leader [30,38,40,43,51], educator [8,31,36,47,48,52,54], or mentor [37,45,50]. Although the descriptions of the roles were isimilar across studies, the terminology used to define each peer may have implications on the perceptions of the role at the ground level.

Additionally, some studies did not include a peer that shared the experience of living with a CMD. A study from Grenada described the individuals delivering a peer support intervention as motivated community lay-person [40]. Community volunteers delivered peer support in a community health centre-based intervention in China [49]. A study from India trained community health workers (CHWs) to interact with patients and provide support [41]. The CHWs were not previously part of the healthcare system, had spoken and written knowledge of the local language, and received training in chronic disease management ahead of the intervention. The implications of a known member of the community delivering support in comparison to a peer living with the same chronic disease are unclear. Considering the limited number of healthcare professionals available in LMIC to deliver support, community members with the appropriate training in chronic disease management may be a viable solution for accessing care. Similar to peer support, this approach also allows for patients to build the social and emotional bonds that come with connecting with a familiar member of the community.

The included studies in this review also demonstrated varied approaches to organizing peer support networks. The three observational studies from Cambodia [47,48,52] were launched out of the MoPoTsyo program, a non-governmental organization incorporating a community-centred approach when delivering long-term care in the management of hypertension and diabetes [52]. This program implements a standardized six-week training course for peers selected to participate in a peer support program designed in collaboration with physicians, pharmacists, and experienced peer educators [47]. In contrast, the RCT from India [41] recruited individuals from the community to be trained as CHWs and implemented the intervention without a clear definition of peer support to guide the intervention. The CHWs received a flipbook aid to reference during visits with patients and participated in inconsistent training throughout the intervention focused on a specific disease or self-management task.

Most studies focused on clinical outcomes and the more frequent outcome investigated in response to the peer support interventions was Hb1Ac. Many studies had cardiovascular risk factors as primary outcomes, which is desirable for cardiovascular disease prevention. The follow-up period varied between eight weeks to three years, with most studies lasting for 12 months. In some studies, limited funding was implicated for the short duration of follow-up which in turn may have influenced their results.

The current scoping review found that about two-thirds of the research regarding peer support for patients with CMDs including T2DM in LMICs were published in the last five years, indicating an increased interest in this topic for better patient outcomes. Among the reasons for the increased efforts to implement peer support programs are the global shortage of healthcare providers and the financial burden associated with healthcare services as these peer support programs appear to be cost-effective [58]. Peer support in LMICs may also be increasing in prevalence because healthcare systems have prioritized the delivery of care for those with acute conditions [52]. LMICs are beginning to implement strategies to address the difficulties patients with chronic conditions face when accessing healthcare as there are fewer CMD specialists or specialty clinics available. The community-driven nature of peer support fosters a sense of autonomy for patients in managing their chronic condition while connecting with a network of individuals that understand their unique life experiences.

Although most of the peer support programs are conducted in emerging countries, there is an orientation toward establishing such programs even in high-income countries [58]. As peer support focuses on bringing together individuals who connect through their shared experiences, the social and emotional benefits introduce a holistic perspective to diabetes management. Additionally, the provision of knowledge based on lived experience is invaluable. As peer educators are often used when traditional forms of healthcare are more difficult to access, the role of peer support is largely unknown within a more established healthcare system [47]. There is much to uncover regarding the effective development and implementation of these programs and more information is needed on the long-term outcomes of these programs on diabetes management [48].

### Strengths and limitations

While intended as a scoping review, we used a methodological and rigorous process, similar in methods to a systematic review with duplication and verifications in screening and data extraction. Our review was thorough as we searched seven databases. Our diverse inclusion criteria and timely review of the literature allowed for a greater depth of insights from the included studies. It is important to note that we did not critically appraise the literature; however, this is not the aim of the scoping review. Furthermore, the heterogeneity around the definitions of peer support, and the paucity of data from LMIC rendered a critical appraisal difficult.

Due to the definition of peer support being varied across studies, the included studies support a theoretical gap existing in understanding what peer support is and how to implement it in different contexts [59]. The use of mapping based on the five components of peer support [6] limited our analysis to focusing only on the effectiveness of interventions. As some of the interventions did not incorporate each of the components in the model, our approach to analyzing the findings may not have captured other dimensions of peer coaching unique to each intervention. Additionally, an empirical gap exists in understanding how to deliver effective programming [59]. Many of the studies lacked program evaluation, limiting the evidence available to suggest an effective methodology to follow when implementing peer support in LMICs.

## Conclusion

This scoping review highlights the different ways in which peer support is defined and utilized as a component of T2DM management. Findings from the included group of studies show the use of peer support in promoting self-management behaviours and influencing mental, social, and emotional dimensions related to health. There is an opportunity for future research to consider the different definitions of what a peer can look like in a community setting, explore the impact of the terminology used to define a peer (i.e., mentor, leader, champion) on study outcomes, and to investigate the effectiveness of peer support interventions in LMICs. We also recommend that future research aims to standardize a definition of peer support, offer more comprehensive descriptions when presenting peer support outcomes, and implement programmatic monitoring and evaluation when delivering interventions to determine how to best implement these programs.

## Data Accessibility Statement

The main study data is the qualitative data extraction of included papers, most of which are included in the manuscript tables. Any other supporting data relating to this review is available from the authors.

## Additional Files

The additional files for this article can be found as follows:

10.5334/gh.1299.s1 Supplemental File 1. Search Strategy.

10.5334/gh.1299.s2 Supplemental File 2. Characteristics of Included Studies.

10.5334/gh.1299.s3 Supplemental File 3. TIDiER.
