# Supplementary material for: Peer Support for Type 2 Diabetes Management in Low- and Middle-Income Countries (LMICs): A Scoping Review
Source: Glob Heart. 2024 Feb 20;19(1):20. doi: 10.5334/gh.1299 (PMC10885823; doi:10.5334/gh.1299)
Supplement: Supplemental File 1. — Search Strategy. [file gh-19-1-1299-s1.pdf]

Supplemental File 1. Search Strategy

DIABETES PEER SUPPORT LMIC COUNTRIES
MARCH 2022

Database: OVID **Medline** Epub Ahead of Print, In-Process & Other Non-Indexed Citations, Ovid MEDLINE(R) Daily and Ovid MEDLINE(R) 1946 to Present
Search Strategy:
----
1 exp Diabetes Mellitus/ (472726)
2 cardiometabolic health.mp. (1845)
3 exp Cardiovascular Diseases/ (2594936)
4 diabetes.ti,ab,kw. (590241)
5 (cardiovascular or coronary artery disease or coronary heart disease, or myocardial or isch#emic heart disease?).tw. (877371)
6 Metabolic Syndrome/ (35750)
7 (metabolic adj2 syndrome?).ti,ab. (57790)
8 exp Stroke/ (156423)
9 ((brain vascular or cerebrovascular or cerebral vascular) adj2 (accident or stroke)).ti,ab. (6088)
10 CVA.ti,ab,kw. or cerebrovascular.kw. or cerebral vascular.kw. or metabolic syndrome.kw. (16365)
11 stroke.ti,ab,kw. (278712)
12 exp Hypertension/ (304787)
13 hypertension.ti,ab,kw. (423518)
14 exp Dyslipidemias/ (84454)
15 dyslipid#emia?.ti,ab,kw. (6416)
16 (hypercholesterol#emia or hyperlipid#emia).ti,ab,kw. (9654)
17 exp obesity/ or weight gain/ (266958)
18 (obes* or (overweight or over weight or overeat* or over eat*)).ti,ab,kw. (370104)
19 ((bmi or body mass index) adj2 (gain or loss or change)).ti,ab,kw. (5359)
20 or/1-19 (3900403)
21 peer support.ti,ab,kw. (5825)
22 (peer and support).ti,ab,kw. (17352)
23 exp Self-Help Groups/ (10563)
24 self-help.ti,ab,kw. (7141)
25 support group?.ti,ab,kw. (8217)
26 psychosocial support group?.ti,ab,kw. (32)
27 exp health Education/ (257060)
28 education.ti,ab,kw. (519552)
29 (lay health worker? or lay worker? or lay health advisor? or volunteer health advisor? or community health worker? or health advisor? or outreach worker? or health coach or lay support).ti,ab,kw. (7562)
30 Patient Navigation/ (935)
31 patient navigator?.ti,ab,kw. (640)

32 or/21-31 (745830)
33 20 and 32 (98204)
34 Developing Countries/ (78943)
35 developing countr*.ti,ab,kw. (96082)
36 low* middle income countr*.ti,ab,kw. (5747)
37 limited resource?.ti,ab,kw. (10942)
38 developing world.ti,ab,kw. (9225)
39 LMIC.ti,ab,kw. (3047)
40 ("low and middle income" adj2 (countr* or nation)).ti,ab,kw. (20894)
41 "low or middle income".tw. adj2 (countr* or nation).ti,ab,kw. (378)
42 third world nations.ti,ab,kw. (45)
43 (Afghanistan or Albania or Algeria or Angola or Antigua or Barbuda or Argentina or Armenia or Azerbaijan).ti,ab,kw,sh. (44702)
44 (Bangladesh or Belarus or Belize or Benin or Bhutan or Bolivia or Bosnia or Herzegovina or Botswana or Brazil or Burkina Faso or Burundi).ti,ab,kw,sh. (180627)
45 (Cabo Verde or Cambodia or Cameroon or Central African Republic or Chad or China or Colombia or Comoros or Congo or Costa Rica or Cote d'Ivoire or Cuba).ti,ab,kw,sh. (390231)
46 (Djibouti or Dominica or Dominican Republic or Ecuador or Egypt or El Salvador or Equatorial Guinea or Eritrea or Eswatini or Ethiopia).ti,ab,kw,sh. (61933)
47 (Fiji or Gabon or Gambia or Georgia or Ghana or Grenada or Guatemala or Guinea or Guinea-Bissau or Guyana or Haiti or Honduras or India or Indonesia or Iran or Iraq).ti,ab,kw,sh. (410785)
48 (Jamaica or Jordan or Kazakhstan or Kenya or Kiribati or Korea or Kosovo or Kyrgyzstan or Lao People's Democratic Republic or Lebanon or Lesotho or Liberia or Libya).ti,ab,kw,sh. (121982)
49 (North Macedonia or Madagascar or Malawi or Malaysia or Maldives or Mali or Marshall Islands or Mauritania or Mauritius or Mexico or Micronesia or Moldova or Mongolia or Montenegro or Montserrat or Morocco or Mozambique or Myanmar).ti,ab,kw,sh. (137515)
50 (Namibia or Nauru or Nepal or Nicaragua or Niger or Nigeria or Niue or Pakistan or Palau or Panama or Papua New Guinea or Paraguay or Peru or Philippines).ti,ab,kw,sh. (142284)
51 (Rwanda or Saint Helena or Samoa or Sao Tome or Senegal or Serbia or Sierra Leone or Solomon Islands or Somalia or South Africa or South Sudan or Sri Lanka or Saint Lucia or Saint Vincent or Grenadines or Sudan or Suriname or Syrian Arab Republic).ti,ab,kw,sh. (107017)
52 (Tajikistan or Tanzania or Thailand or Timor-Leste or Togo or Tokelau or Tonga or Tunisia or Turkey or Turkmenistan or Tuvalu or Uganda or Ukraine or Uzbekistan or Vanuatu or Venezuela or Vietnam or Wallis or Futuna or West Bank or Gaza Strip or Yemen or Zambia or Zimbabwe).ti,ab,kw,sh. (237251)
53 or/34-52 (1752229)
54 33 and 53 (14013)
55 randomized controlled trial.pt. (562242)
56 controlled clinical trial.pt. (94759)
57 Random Allocation/ (106775)
58 Double-Blind Method/ (170801)
59 single-blind method/ (31729)

60 *Research Design/ (41347)
61 evaluation studies/ (261684)
62 Comparative Study/ (1910701)
63 exp Longitudinal Studies/ (156426)
64 cross-over studies/ (53073)
65 clinical trial.tw. (167417)
66 clinical trial.pt. (534454)
67 latin square.tw. (5149)
68 (time adj series).tw. (38177)
69 (before adj2 after adj3 (stud$ or trial$ or design$)).tw. (15408)
70 random$.tw. (1300360)
71 (matched communities or matched schools or matched populations).tw. (343)
72 control$.tw. (4264247)
73 (comparison group$ or control group$).tw. (537214)
74 matched pairs.tw. (7158)
75 (outcome study or outcome studies).tw. (8443)
76 (quasiexperimental or quasi experimental or pseudo experimental).tw. (17373)
77 (nonrandomi?ed or non randomi?ed or pseudo randomi?sed or quasi randomi?ed).tw. (34358)
78 prospectiv$.tw. (795467)
79 volunteer$.tw. (206459)
80 exp epidemiological studies/ or cross-sectional study.mp. or cross-sectional studies.mp. or before-after study.mp. or case-control study.mp. or cohort study.mp. or cohort studies.mp. or population-based study.mp. or exp "surveys and questionnaires"/ (3815976)
81 (systematic review or meta-analysis).mp,pt. or metaanalysis.mp. (371718)
82 (retrospective adj2 (study or trial* or studies)).tw. (312418)
83 retrospective study.kw. (1319)
84 survey?.ti,ab,kw. (699499)
85 or/55-84 (10017018)
86 54 and 85 (11505)

Database**: Embase <**1974 to 2022 March 23>
Search Strategy:
----
1 randomization/ (93315)
2 exp clinical trial/ (1681627)
3 exp Double-Blind procedure/ (193387)
4 exp Single-Blind procedure/ (45568)
5 exp Crossover procedure/ (69764)
6 clinical trial.tw. (246369)
7 ((singl$ or doubl$ or treble$ or tripl$) and (mask$ or blind$)).tw. (299611)
8 latin square.tw. (5508)
9 placebo/ (378316)
10 placebo$.tw. (340380)

11 random$.tw. (1767937)
12 Comparative Study/ (941802)
13 evaluation/ (178246)
14 clinical trial.tw. (246369)
15 latin square.tw. (5508)
16 (before adj2 after adj3 (stud$ or trial$ or design$)).tw. (20494)
17 ((singl$ or doubl$ or trebl$ or tripl$) adj5 (blind$ or mask)).tw. (260469)
18 (matched communities or matched schools or matched populations).tw. (469)
19 control$.tw. (5548271)
20 (comparison group$ or control group$).tw. (768142)
21 matched pairs.tw. (10712)
22 (outcome study or outcome studies).tw. (12732)
23 (quasiexperimental or quasi experimental or pseudo experimental).tw. (21660)
24 (nonrandomi?ed or non randomi?ed or pseudo randomi?sed or quasi randomi?ed).tw. (46604)
25 prospectiv$.tw. (1230183)
26 volunteer$.tw. (277649)
27 exp Randomized Controlled Trial/ (702130)
28 Cross-sectional study/ or exp clinical study/ or cross-sectional study.mp. or cross-sectional studies.mp. or before-after study.mp. or case-control study.mp. or cohort study.mp. or cohort studies.mp. or population-based study.mp. (11365164)
29 Systematic review.mp. or exp meta analysis/ or meta-analysis.ti,ab. or metaanalysis.ti,ab. (562238)
30 exp health survey/ (247571)
31 exp clinical study/ (11046106)
32 retrospective study.mp. (1258670)
33 or/1-32 (16526872)
34 exp diabetes mellitus/ (1085511)
35 exp cardiovascular disease/ (4509415)
36 cardiometabolic health.ti,ab,kw. (2244)
37 diabetes.ti,ab,kw. (903008)
38 (cardiovascular or coronary artery disease or coronary heart disease, or myocardial or isch#emic heart disease?).ti,ab,kw. (1264915)
39 metabolic syndrome X/ (92599)
40 (metabolic adj2 syndrome?).ti,ab. (90178)
41 exp cerebrovascular accident/ (259699)
42 ((brain vascular or cerebrovascular or cerebral vascular) adj2 (accident or stroke)).ti,ab. (10128)
43 CVA.ti,ab,kw. or cerebrovascular.kw. or cerebral vascular.kw. or metabolic syndrome.kw. (34325)
44 stroke.ti,ab,kw. (448055)
45 exp hypertension/ (823580)
46 hypertension.ti,ab,kw. (650917)
47 dyslipidemia/ (84848)

48 (hypercholesterol#emia or hyperlipid#emia or dyslipid#emia?).ti,ab,kw. (23878)
49 exp obesity/ (586751)
50 weight gain/ (98883)
51 weight reduction/ (171231)
52 (obes$ or weight gain or weight loss or overweight or over weight or overeat$ or over eat$ or weight change$).ti,ab,kw. (721467)
53 ((bmi or body mass index) adj2 (gain or loss or change)).ti,ab,kw. (9105)
54 or/34-53 (6128335)
55 33 and 54 (3946762)
56 peer support.ti,ab,kw. (8086)
57 self help/ (14157)
58 self-help.ti,ab,kw. (9107)
59 exp support group/ (13688)
60 support group?.ti,ab,kw. (12901)
61 diabetes education/ or health education/ (105939)
62 education.ti,ab,kw. (683306)
63 (lay health worker? or lay worker? or lay health advisor? or volunteer health advisor? or community health worker? or health advisor? or outreach worker? or health coach or lay support).ti,ab,kw. (9542)
64 lay health worker/ (162)
65 patient navigat*.ti,ab,kw. (2437)
66 or/56-65 (785873)
67 developing country/ (97902)
68 middle income country/ (14645)
69 low income country/ (10163)
70 developing countr*.ti,ab,kw. (88757)
71 low* middle income countr*.ti,ab,kw. (6831)
72 limited resource?.ti,ab,kw. (15037)
73 developing world.ti,ab,kw. (11679)
74 LMIC.ti,ab,kw. (4110)
75 ("low and middle income" adj2 (countr* or nation?)).ti,ab,kw. (24394)
76 ("low or middle income" adj2 (countr* or nation?)).ti,ab,kw. (453)
77 third world nations.ti,ab,kw. (51)
78 (Afghanistan or Albania or Algeria or Angola or Antigua or Barbuda or Argentina or Armenia or Azerbaijan).ti,ab,kw,sh. (55931)
79 (Bangladesh or Belarus or Belize or Benin or Bhutan or Bolivia or Bosnia or Herzegovina or Botswana or Brazil or Burkina Faso or Burundi).ti,ab,kw,sh. (207378)
80 (Cabo Verde or Cambodia or Cameroon or Central African Republic or Chad or China or Colombia or Comoros or Congo or Costa Rica or Cote d'Ivoire or Cuba).ti,ab,kw,sh. (428777)
81 (Djibouti or Dominica or Dominican Republic or Ecuador or Egypt or El Salvador or Equatorial Guinea or Eritrea or Eswatini or Ethiopia).ti,ab,kw,sh. (69636)
82 (Fiji or Gabon or Gambia or Georgia or Ghana or Grenada or Guatemala or Guinea or Guinea-Bissau or Guyana or Haiti or Honduras or India or Indonesia or Iran or Iraq).ti,ab,kw,sh. (479876)

83 (Jamaica or Jordan or Kazakhstan or Kenya or Kiribati or Korea or Kosovo or Kyrgyzstan or Lao People's Democratic Republic or Lebanon or Lesotho or Liberia or Libya).ti,ab,kw,sh. (151083)
84 (North Macedonia or Madagascar or Malawi or Malaysia or Maldives or Mali or Marshall Islands or Mauritania or Mauritius or Mexico or Micronesia or Moldova or Mongolia or Montenegro or Montserrat or Morocco or Mozambique or Myanmar).ti,ab,kw,sh. (158076)
85 (Namibia or Nauru or Nepal or Nicaragua or Niger or Nigeria or Niue or Pakistan or Palau or Panama or Papua New Guinea or Paraguay or Peru or Philippines).ti,ab,kw,sh. (171274)
86 (Rwanda or Saint Helena or Samoa or Sao Tome or Senegal or Serbia or Sierra Leone or Solomon Islands or Somalia or South Africa or South Sudan or Sri Lanka or Saint Lucia or Saint Vincent or Grenadines or Sudan or Suriname or Syrian Arab Republic).ti,ab,kw,sh. (122644)
87 (Tajikistan or Tanzania or Thailand or Timor-Leste or Togo or Tokelau or Tonga or Tunisia or Turkey or Turkmenistan or Tuvalu or Uganda or Ukraine or Uzbekistan or Vanuatu or Venezuela or Vietnam or Wallis or Futuna or West Bank or Gaza Strip or Yemen or Zambia or Zimbabwe).ti,ab,kw,sh. (260177)
88 or/67-87 (2000422)
89 55 and 66 and 88 (16268)

Database: **Ovid Emcare** <1995 to 2022 Week 11>
Search Strategy:
----
1 randomization/ (19165)
2 exp clinical trial/ (436540)
3 exp Double-Blind procedure/ (53320)
4 exp Single-Blind procedure/ (14052)
5 exp Crossover procedure/ (20487)
6 clinical trial.tw. (64744)
7 ((singl$ or doubl$ or treble$ or tripl$) and (mask$ or blind$)).tw. (73923)
8 latin square.tw. (496)
9 placebo/ (106129)
10 placebo$.tw. (80244)
11 random$.tw. (522922)
12 Comparative Study/ (134132)
13 evaluation/ (11266)
14 clinical trial.tw. (64744)
15 latin square.tw. (496)
16 (before adj2 after adj3 (stud$ or trial$ or design$)).tw. (7194)
17 ((singl$ or doubl$ or trebl$ or tripl$) adj5 (blind$ or mask)).tw. (65223)
18 (matched communities or matched schools or matched populations).tw. (149)
19 control$.tw. (1111642)
20 (comparison group$ or control group$).tw. (192432)
21 matched pairs.tw. (2998)
22 (outcome study or outcome studies).tw. (4955)
23 (quasiexperimental or quasi experimental or pseudo experimental).tw. (14583)

24 (nonrandomi?ed or non randomi?ed or pseudo randomi?sed or quasi randomi?ed).tw. (14749)
25 prospectiv$.tw. (311022)
26 volunteer$.tw. (67184)
27 exp Randomized Controlled Trial/ (215637)
28 Cross-sectional study/ or exp clinical study/ or cross-sectional study.mp. or cross-sectional studies.mp. or before-after study.mp. or case-control study.mp. or cohort study.mp. or cohort studies.mp. or population-based study.mp. (2650667)
29 Systematic review.mp. or exp meta analysis/ or meta-analysis.ti,ab. or metaanalysis.ti,ab. (211954)
30 exp health survey/ (85055)
31 exp clinical study/ (2549480)
32 retrospective study.mp. (262067)
33 or/1-32 (3583831)
34 exp diabetes mellitus/ (246205)
35 exp cardiovascular disease/ (951671)
36 cardiometabolic health.ti,ab,kw. (972)
37 diabetes.ti,ab,kw. (202284)
38 (cardiovascular or coronary artery disease or coronary heart disease, or myocardial or isch#emic heart disease?).ti,ab,kw. (266113)
39 metabolic syndrome X/ (21239)
40 (metabolic adj2 syndrome?).ti,ab. (19758)
41 exp cerebrovascular accident/ (65490)
42 ((brain vascular or cerebrovascular or cerebral vascular) adj2 (accident or stroke)).ti,ab. (2089)
43 CVA.ti,ab,kw. or cerebrovascular.kw. or cerebral vascular.kw. or metabolic syndrome.kw. (8808)
44 stroke.ti,ab,kw. (112413)
45 exp hypertension/ (170741)
46 hypertension.ti,ab,kw. (106067)
47 dyslipidemia/ (18495)
48 (hypercholesterol#emia or hyperlipid#emia or dyslipid#emia?).ti,ab,kw. (4098)
49 exp obesity/ (167089)
50 weight gain/ (27164)
51 weight reduction/ (45140)
52 (obes$ or weight gain or weight loss or overweight or over weight or overeat$ or over eat$ or weight change$).ti,ab,kw. (184484)
53 ((bmi or body mass index) adj2 (gain or loss or change)).ti,ab,kw. (2649)
54 or/34-53 (1346435)
55 33 and 54 (891103)
56 peer support.ti,ab,kw. (5029)
57 self help/ (5997)
58 self-help.ti,ab,kw. (4411)
59 exp support group/ (8886)

60 support group?.ti,ab,kw. (5824)
61 diabetes education/ or health education/ (43229)
62 education.ti,ab,kw. (311337)
63 (lay health worker? or lay worker? or lay health advisor? or volunteer health advisor? or community health worker? or health advisor? or outreach worker? or health coach or lay support).ti,ab,kw. (5016)
64 lay health worker/ (116)
65 patient navigat*.ti,ab,kw. (943)
66 or/56-65 (353569)
67 developing country/ (22833)
68 middle income country/ (7499)
69 low income country/ (4549)
70 developing countr*.ti,ab,kw. (25801)
71 low* middle income countr*.ti,ab,kw. (3386)
72 limited resource?.ti,ab,kw. (5173)
73 developing world.ti,ab,kw. (3380)
74 LMIC.ti,ab,kw. (1691)
75 ("low and middle income" adj2 (countr* or nation?)).ti,ab,kw. (11431)
76 ("low or middle income" adj2 (countr* or nation?)).ti,ab,kw. (215)
77 third world nations.ti,ab,kw. (8)
78 (Afghanistan or Albania or Algeria or Angola or Antigua or Barbuda or Argentina or Armenia or Azerbaijan).ti,ab,kw,sh. (11979)
79 (Bangladesh or Belarus or Belize or Benin or Bhutan or Bolivia or Bosnia or Herzegovina or Botswana or Brazil or Burkina Faso or Burundi).ti,ab,kw,sh. (53550)
80 (Cabo Verde or Cambodia or Cameroon or Central African Republic or Chad or China or Colombia or Comoros or Congo or Costa Rica or Cote d'Ivoire or Cuba).ti,ab,kw,sh. (99775)
81 (Djibouti or Dominica or Dominican Republic or Ecuador or Egypt or El Salvador or Equatorial Guinea or Eritrea or Eswatini or Ethiopia).ti,ab,kw,sh. (20079)
82 (Fiji or Gabon or Gambia or Georgia or Ghana or Grenada or Guatemala or Guinea or Guinea-Bissau or Guyana or Haiti or Honduras or India or Indonesia or Iran or Iraq).ti,ab,kw,sh. (105894)
83 (Jamaica or Jordan or Kazakhstan or Kenya or Kiribati or Korea or Kosovo or Kyrgyzstan or Lao People's Democratic Republic or Lebanon or Lesotho or Liberia or Libya).ti,ab,kw,sh. (41212)
84 (North Macedonia or Madagascar or Malawi or Malaysia or Maldives or Mali or Marshall Islands or Mauritania or Mauritius or Mexico or Micronesia or Moldova or Mongolia or Montenegro or Montserrat or Morocco or Mozambique or Myanmar).ti,ab,kw,sh. (40453)
85 (Namibia or Nauru or Nepal or Nicaragua or Niger or Nigeria or Niue or Pakistan or Palau or Panama or Papua New Guinea or Paraguay or Peru or Philippines).ti,ab,kw,sh. (38154)
86 (Rwanda or Saint Helena or Samoa or Sao Tome or Senegal or Serbia or Sierra Leone or Solomon Islands or Somalia or South Africa or South Sudan or Sri Lanka or Saint Lucia or Saint Vincent or Grenadines or Sudan or Suriname or Syrian Arab Republic).ti,ab,kw,sh. (34394)
87 (Tajikistan or Tanzania or Thailand or Timor-Leste or Togo or Tokelau or Tonga or Tunisia or Turkey or Turkmenistan or Tuvalu or Uganda or Ukraine or Uzbekistan or Vanuatu or

Venezuela or Vietnam or Wallis or Futuna or West Bank or Gaza Strip or Yemen or Zambia or Zimbabwe).ti,ab,kw,sh. (66621)
88 or/67-87 (486852)
89 55 and 66 and 88 (6071)

Database: APA PsycInfo <1806 to March Week 3 2022>
Search Strategy:
----
1 exp diabetes/ (19448)
2 exp cardiovascular disorders/ (67238)
3 diabetes.ti,ab. (31665)
4 (cardiovascular or coronary artery disease or coronary heart disease, or myocardial or isch#emic heart disease?).tw. (40739)
5 (metabolic adj2 syndrome?).ti,ab. (3657)
6 cerebrovascular accidents/ (22852)
7 ((brain vascular or cerebrovascular or cerebral vascular) adj2 (accident or stroke)).ti,ab. (863)
8 stroke.ti,ab. (35033)
9 exp hypertension/ (7927)
10 hypertension.tw. (17296)
11 dyslipid#emia.tw. (211)
12 (hypercholesterol#emia or hyperlipid#emia).tw. (216)
13 obesity/ (26867)
14 (obes* or overweight or over weight or overeat* or over eat*).tw. (50602)
15 ((bmi or body mass index or weight) adj2 (gain or loss or change)).tw. (24054)
16 or/1-15 (185191)
17 exp support groups/ (6263)
18 (peer? and support).tw. (25453)
19 self-help.tw. (9627)
20 support group?.tw. (8513)
21 health education/ (14113)
22 education.tw. (405090)
23 (lay health worker? or lay worker? or lay health advisor? or volunteer health advisor? or community health worker? or health advisor? or outreach worker? or health coach or lay support).tw. (2102)
24 coaching/ or life coaching/ (4881)
25 patient navigat*.tw. (384)
26 or/17-25 (452002)
27 16 and 26 (15928)
28 developing countries/ (6010)
29 developing countr*.tw. (9951)
30 low* middle income countr*.tw. (491)
31 limited resource?.tw. (3534)
32 developing world.tw. (1565)

33 LMIC.tw. (585)
34 "low and middle income".tw. (4273)
35 "low or middle income".tw. (99)
36 (third world nations or third world countr*).tw. (301)
37 (Afghanistan or Albania or Algeria or Angola or Antigua or Barbuda or Argentina or Armenia or Azerbaijan).tw. (7835)
38 (Bangladesh or Belarus or Belize or Benin or Bhutan or Bolivia or Bosnia or Herzegovina or Botswana or Brazil or Burkina Faso or Burundi).tw. (19396)
39 (Cabo Verde or Cambodia or Cameroon or Central African Republic or Chad or China or Colombia or Comoros or Congo or Costa Rica or Cote d'Ivoire or Cuba).tw. (44397)
40 (Djibouti or Dominica or Dominican Republic or Ecuador or Egypt or El Salvador or Equatorial Guinea or Eritrea or Eswatini or Ethiopia).tw. (6779)
41 (Fiji or Gabon or Gambia or Georgia or Ghana or Grenada or Guatemala or Guinea or Guinea-Bissau or Guyana or Haiti or Honduras or India or Indonesia or Iran or Iraq).tw. (48878)
42 (Jamaica or Jordan or Kazakhstan or Kenya or Kiribati or Korea or Kosovo or Kyrgyzstan or Lao People's Democratic Republic or Lebanon or Lesotho or Liberia or Libya).tw. (22130)
43 (North Macedonia or Madagascar or Malawi or Malaysia or Maldives or Mali or Marshall Islands or Mauritania or Mauritius or Mexico or Micronesia or Moldova or Mongolia or Montenegro or Montserrat or Morocco or Mozambique or Myanmar).tw. (20700)
44 (Namibia or Nauru or Nepal or Nicaragua or Niger or Nigeria or Niue or Pakistan or Palau or Panama or Papua New Guinea or Paraguay or Peru or Philippines).tw. (17682)
45 (Tajikistan or Tanzania or Thailand or Timor-Leste or Togo or Tokelau or Tonga or Tunisia or Turkey or Turkmenistan or Tuvalu or Uganda or Ukraine or Uzbekistan or Vanuatu or Venezuela or Vietnam or Wallis or Futuna or West Bank or Gaza Strip or Yemen or Zambia or Zimbabwe).tw. (32031)
46 (Rwanda or Saint Helena or Samoa or Sao Tome or Senegal or Serbia or Sierra Leone or Solomon Islands or Somalia or South Africa or South Sudan or Sri Lanka or Saint Lucia or Saint Vincent or Grenadines or Sudan or Suriname or Syrian Arab Republic).tw. (18923)
47 or/28-46 (223441)
48 27 and 47 (1306)

COCHRANE

Search Name: DM Peer Support LMIC
Date Run: 26/03/2022 10:33:08
Comment:

ID Search Hits
#1 MeSH descriptor: [Diabetes Mellitus] explode all trees 34485
#2 cardiometabolic next health 845
#3 MeSH descriptor: [Cardiovascular Diseases] explode all trees 115677
#4 diabetes 96751
#5 cardiovascular or (coronary next artery next disease) or (coronary next heart next disease) or myocardial or(isch?emic next heart next disease?) 141504
#6 MeSH descriptor: [Metabolic Syndrome] explode all trees 2030

#7 metabolic near/2 syndrome? 8538
#8 MeSH descriptor: [Stroke] explode all trees 11143
#9 (("brain vascular" or cerebrovascular or "cerebral vascular") near/2 (accident or stroke)) 15709
#10 cva or stroke or "metabolic syndrome" 84177
#11 MeSH descriptor: [Hypertension] explode all trees 19593
#12 hypertension 70232
#13 MeSH descriptor: [Dyslipidemias] explode all trees 7853
#14 dyslipid?emia? or hypercholesterol?emia or hyperlipid?emia 17406
#15 MeSH descriptor: [Obesity] explode all trees 15455
#16 MeSH descriptor: [Weight Gain] explode all trees 2759
#17 obes* or overweight or over weight or overeat* or (over next eat*) 80424
#18 ((bmi or (body next mass next index)) near/2 (gain or loss or change)) 1669
#19 #1 or #2 or #3 or #4 or #5 or #6 or #7 or #8 or #9 or #10 or #11 or #12 or #13 or #14 or #15 or #16 368797
#20 peer next support 1516
#21 MeSH descriptor: [Self-Help Groups] explode all trees 797
#22 support next group? 2335
#23 psychosocial next support 751
#24 MeSH descriptor: [Health Educators] explode all trees 29
#25 education and (health or diabetes or "weight loss" or "weight control" or "weight management") 54930
#26 ((lay next health) next worker?) or (lay next worker?) or (lay next health next advisor?) or ((volunteer next health) next advisor?) or ((community next health) next worker?) or (health next advisor?) or (outreach next worker?) or (health next coach*) or (lay next support) 3359
#27 MeSH descriptor: [Patient Navigation] explode all trees 149
#28 patient next navigat* 633
#29 #20 or #21 or #22 or #23 or #24 or #25 or #26 or #27 or #28 60892
#30 #19 and #29 16499
#31 developing next countr* 4957
#32 MeSH descriptor: [Developing Countries] explode all trees 909
#33 low* next middle next income next countr* 392
#34 limited next resource? 859
#35 developing next world 515
#36 LMIC 333
#37 ("low and middle income" near/2 (countr* or nation?)) 2236
#38 "low or middle income".tw. near/2 (countr* or nation?) 196
#39 third next world next nation? 6
#40 (Afghanistan or Albania or Algeria or Angola or Antigua or Barbuda or Argentina or Armenia or Azerbaijan) 4518
#41 (Bangladesh or Belarus or Belize or Benin or Bhutan or Bolivia or Bosnia or Herzegovina or Botswana or Brazil or Burkina Faso or Burundi) 21516
#42 (Cabo Verde or Cambodia or Cameroon or Central African Republic or Chad or China or Colombia or Comoros or Congo or Costa Rica or Cote d'Ivoire or Cuba) 64692

#43 (Djibouti or Dominica or Dominican Republic or Ecuador or Egypt or El Salvador or Equatorial Guinea or Eritrea or Eswatini or Ethiopia) 9916
#44 (Fiji or Gabon or Gambia or Georgia or Ghana or Grenada or Guatemala or Guinea or Guinea-Bissau or Guyana or Haiti or Honduras or India or Indonesia or Iran or Iraq) 60056
#45 (Jamaica or Jordan or Kazakhstan or Kenya or Kiribati or Korea or Kosovo or Kyrgyzstan or Lao People's Democratic Republic or Lebanon or Lesotho or Liberia or Libya) 27501
#46 (North Macedonia or Madagascar or Malawi or Malaysia or Maldives or Mali or Marshall Islands or Mauritania or Mauritius or Mexico or Micronesia or Moldova or Mongolia or Montenegro or Montserrat or Morocco or Mozambique or Myanmar) 14372
#47 (Namibia or Nauru or Nepal or Nicaragua or Niger or Nigeria or Niue or Pakistan or Palau or Panama or Papua New Guinea or Paraguay or Peru or Philippines) 13054
#48 Rwanda or Saint Helena or Samoa or Sao Tome or Senegal or Serbia or Sierra Leone or Solomon Islands or Somalia or South Africa or South Sudan or Sri Lanka or Saint Lucia or Saint Vincent or Grenadines or Sudan or Suriname or Syrian Arab Republic 11093
#49 Tajikistan or Tanzania or Thailand or Timor-Leste or Togo or Tokelau or Tonga or Tunisia or Turkey or Turkmenistan or Tuvalu or Uganda or Ukraine or Uzbekistan or Vanuatu or Venezuela or Vietnam or Wallis or Futuna or West Bank or Gaza Strip or Yemen or Zambia or Zimbabwe 37372
#50 #31 or #32 or #33 or #34 or #35 or #36 or #37 or #38 or #39 or #40 or #41 or #42 or #43 or #44 or #45 or #46 or #47 or #48 or #49 242327
#51 #30 and #50 4015
#52 #30 and #50 in Cochrane Reviews 997
#53 #30 and #50 in Trials 2866

LILACS
diabetes or (cardiovascular disease) or (cardiovascular diseases) or (coronary heart disease) or (coronary artery disease) or hypertension or (ischemic heart disease) or (ischaemic heart disease) or (Cerebrovascular accident) or stroke or (brain vascular accident) or (cerebral vascular accident) or dyslipidemia or hyperlipidemia or hypercholesterolemia or obese or oebsity or "weight loss" or "weight gain" or "weight management" or "weight reduction" or "weight control" or overweight or overeat
AND
(community health worker) or (support group) or (support groups) or (peer support) or (outreach worker) or (outreach workers) or (self help) or (lay health worker) or (Lay health workers) or (lay worker) or (lay workers) or (patient navigator) or (patient navigators) or (patient navigation) or (health advisor) or (health advisors) or (health coach) or (diabetes education) or (health education)
638 refs
