# Supplementary material for: Peer Support for Type 2 Diabetes Management in Low- and Middle-Income Countries (LMICs): A Scoping Review
Source: Glob Heart. 2024 Feb 20;19(1):20. doi: 10.5334/gh.1299 (PMC10885823; doi:10.5334/gh.1299)
Supplement: Supplemental File 2. — Characteristics of Included Studies. [file gh-19-1-1299-s2.pdf]

Supplemental File 2: Characteristics of Included Studies

Author, Year	Ahmadi, 2018
Location (city, country)	Iran
Objective	Compared the effect of education by health care provider (CP) and peer (PE) on self-care behaviors among Iranian patients with diabetes.
Study Design	Clinical randomized control trial.
Inclusion/Exclusion Criteria	Inclusion: 40-65 years of age; type 2 diabetes; history of diabetes for more than six months and willingness to take part in the study<br><br>Exclusion: suffering from another acute or chronic physical disease (cardiac, respiratory, hepatic, musculoskeletal, or renal); and mental disorders<br><br>Peer educators Inclusion: good control of blood glucose, a few complications, ability to manage sessions, personal interest to collaborate and provide support, good social communication skills (e.g., good appearance, tone of voice, eye contact) and education higher than middle school
Overall n (number invited)	120
Intervention n (number invited)	CP: 40, PE: 40
Control n (number invited)	40
Loss to follow-up, n (%)	0
Age Mean (SD)	NR
Female I: n (%); C: n (%)	CP: 24 (60), PE: 25 (63); C: 25 (63)
Male I: n (%); C: n (%)	CP: 16 (40), PE: 15 (37); C: 15 (37)
Diseases at Baseline	Type 2 diabetes
BMI Mean (SD)	NR
Blood pressure Mean (SD)	NR
HbA1c Mean (SD)	NR
Description of Intervention	Meeting plans included 12 weeks of education, one session per week during the first six weeks and one session every other week during the second six weeks.<br>Each session lasted for one hour. Sessions were held in groups of 20 patients. Session content was designed based on the needs of a diabetes patient with particular attention to the main items of the summary of diabetes self-care activities (SDSCA) questionnaire such as diet control, physical activities, medication adherence and foot care which was all coordinated with the peer. The peer was asked to exchange his experience in diabetes control.
Description/definition of peer support	A peer was a known diabetic with good control of blood glucose, few complications, able to manage sessions, had personal interest to collaborate and provide support, had good social communication skills (e.g., good

	appearance, tone of voice, eye contact) and education higher than middle school. Peers underwent 2 weeks training from the first author.<br><br>Definition: Peer education is an information exchange of attitudes and behaviors from individuals who are not specialists but have similar experiences.
Mode of peer support	Face to face
Setting/Context: Location or Primary site of delivery of intervention	Diabetes Clinic at a university medical center hospital.
Description of Control	Received the clinic's routine diabetes education program and did not undergo any intervention.
Duration of intervention and control	12 weeks
Time points of outcome measures	Baseline, 12 weeks/post intervention.
List of outcomes and measurement tool	Self-care behaviors: SDSCA (summary of diabetes self-care activities questionnaire)
Outcomes mapped to Evans et al	(2) assistance in daily management

Author, Year	Assah, 2015
Location (city, country)	Yaounde, Cameroon
Objective	Examined the effectiveness of a community-based multilevel peer support intervention in addition to usual diabetes care on improving glycaemic levels, blood pressure and lipids in patients with Type 2 diabetes in Yaounde, Cameroon.
Study Design	Non-randomized controlled trial.
Inclusion/Exclusion Criteria	Inclusion: adults with poorly controlled Type 2 diabetes (HbA1c > 7%)<br><br>Exclusion: NR
Overall n (number invited)	192
Intervention n (number invited)	96
Control n (number invited)	96
Loss to follow-up, n (%)	0
Age Mean (SD)	I: 57.1 (9.3); C: 57.2 (9.1)
Female I: n (%); C: n (%)	I: 51 (53); C: 51 (53)
Male I: n (%); C: n (%)	I: 45 (47); C: 45 (47)
Diseases at Baseline	Type 2 diabetes
BMI Mean (SD)	I: 28.6 (3.9)
Blood pressure Mean (SD)	I: SBP: 142.04 (19.52); DBP: 84.44 (11.31)
HbA1c Mean (SD)	I: 9.6 (1.7); C: 9.8 (1.6)
Description of Intervention	The intervention arm underwent a structured community-based multilevel peer support intervention adapted to the sociocultural context of Cameroon, in addition to their usual clinical care.<br>A peer supporter was recruited for each of the 10 groups of the intervention arm based on their past history and clinical profile: better glycaemic and metabolic control than their peers, more compliant with their clinic visits, and more experiential knowledge on diabetes. These peer supporters, who were invited to volunteer, underwent a two–day training workshop that fortified their knowledge and skills to support persons with diabetes, emphasizing on building and reinforcing the participant's knowledge on diabetes, training on communication skills, effective group and face-to-face meetings, and use of personal history as examples in peer support.<br>The peer support intervention was implemented through group meetings, personal encounters between peer supporters and their group members, and telephone calls. There were six group meetings following a standard schedule, five monthly personal encounters and phone calls between peers and group members. Group meetings (on diet and healthy eating, physical exercise, observance to treatment, feet and body care, complications of diabetes and living with diabetes), were held monthly at locations related to

	each group's common affinity, out of the hospital setting.
Description/definition of peer support	A peer supporter was recruited for each of the 10 groups of the intervention arm based on their past history and clinical profile: better glycaemic and metabolic control than their peers, more compliant with their clinic visits, and more experiential knowledge on diabetes. They underwent a two-day training workshop.<br><br>Definition: Peer-support care models provide a low-cost, flexible means to supplement formal healthcare support for chronic diseases.
Mode of peer support	Face to face, phone
Setting/Context: Location or Primary site of delivery of intervention	NR
Description of Control	Usual clinical care
Duration of intervention and control	Six months
Time points of outcome measures	Baseline, 6 months post intervention
List of outcomes and measurement tool	Weight<br><br>height<br><br>blood pressure<br><br>waist and hip circumference<br><br>fat mass<br><br>HbA1c<br><br>fasting glycaemia<br><br>blood lipids<br><br>SDSCA questionnaire
Outcomes mapped to Evans et al	(2) assistance in daily management

Author, Year	Baumann, 2015
Location (city, country)	Mityana, Uganda
Objective	Tested the feasibility of a peer intervention to improve the following: diabetes self-care behaviors, glycemic control, social support and emotional well-being, linkages to health care providers, and to assess the sustainability of the intervention 18 months later.
Study Design	Pre-post quasi-experimental study
Inclusion/Exclusion Criteria	Inclusion: Patients over 18 years old with type 2 diabetes and receiving care at Mityana diabetes clinic<br><br>Exclusion: NR
Overall n (number invited)	60 (Peer Champions (Ch): N = 30, Peer Partners (P): N = 30)
Intervention n (number invited)	N/A
Control n (number invited)	N/A
Loss to follow-up, n (%)	19 (32)
Age Mean (SD)	Median (IQR): Ch: 53 (13), P: 53 (11)
Female I: n (%); C: n (%)	Ch: 10 (63), P: 18 (72)
Male I: n (%); C: n (%)	Ch: 6 (37), P: 7 (28)
Diseases at Baseline	Diabetes
BMI Mean (SD)	O: 26.79 (4.95)
Blood pressure Mean (SD)	O: SBP: 146.34 (24.39), DBP: 85.39 (12.34)
HbA1c Mean (SD)	O: 11.10 (4.30)
Description of Intervention	This single-group pre-post study examined a four-month peer support intervention in which participants were trained in diabetes self-care, some serving as peer champions and others as peer partners. Participants were asked to complete a written contact log after each contact with a peer; a prepaid telephone network was activated among all participants, and call logs were recorded electronically. Measures of diabetes self-care and physiologic outcomes were obtained at a final group meeting four months later (T2).<br>The curriculum addressed areas of diabetes self-care that included healthy eating, being active, taking medications, monitoring blood sugar, problem solving, reducing risks, and problem solving. All participants were given a packet of materials that contained the following: a consent form, the Diabetes Self-Care Questionnaire, Screening Data Form, Take Care of Your Feet poster, Peer Champion Contact Logbook, Peer Champion Training Booklet, and “The ABC’s of Diabetes” brochure.
Description/definition of peer support	Peer champions were patients able to read and speak English who received additional training in communication skills to provide peer partners emotional support and assistance with daily management.

	Definition: Peer support is a promising approach toward achieving self-care goals.
Mode of peer support	Face to face and phone
Setting/Context: Location or Primary site of delivery of intervention	Diabetes clinic
Description of Control	N/A
Duration of intervention and control	Four months
Time points of outcome measures	Baseline, four months post intervention.
List of outcomes and measurement tool	Self-Care: Diabetes Self-Care Questionnaire<br><br>height: measuring tape<br><br>Weight: scale<br><br>BMI: kg/m^2 and estimated using a chart from Boston Medical Center<br><br>blood pressure: aneroid blood pressure cuff and stethoscope<br><br>HbA1c: Roche COBAS Integra 400/700/800 analyzer<br><br>Participant logbooks<br><br>Phone records<br><br>Narrative Notes
Outcomes mapped to Evans et al<br>*as specified in manuscript	(2) assistance in daily management,<br>(3) social and emotional support to promote disease self management and coping with negative emotions<br>(4) linkage to clinical care and community resources<br>(5) ongoing support because chronic disease is for the rest of one's life

Author, Year	Castillo-Hernandez, 2021
Location (city, country)	Komchén, Mexico
Objective	Evaluated the impact of peer support in addition and in comparison to diabetes self-management education (DSME) alone on diabetes control and diabetes-related quality-of-life outcomes in people of Mayan origin with type 2 diabetes.
Study Design	Randomized controlled trial.
Inclusion/Exclusion Criteria	Inclusion: >18 years and a physician's diagnosis of type 2 diabetes<br><br>Exclusion: severe medical or psychiatric conditions, such as hearing impairment or substance abuse
Overall n (number invited)	58
Intervention n (number invited)	29
Control n (number invited)	29
Loss to follow-up, n (%)	O: 4 (7); I: 1 (3); C: 3 (10)
Age Mean (SD)	I: 59 (9.4); C: 56 (10.3)
Female I: n (%); C: n (%)	I: 27 (93); C: 29 (100)
Male I: n (%); C: n (%)	I: 0 (0); C: 2 (7)
Diseases at Baseline	Type 2 diabetes
BMI Mean (SD)	I: 29.2 (5); C: 30.2 (4)
Blood pressure Mean (SD)	I: SBP: 131 (24); DBP: 78 (11); C: SBP: 126 (26); DBP: 79 (14)
HbA1c Mean (SD)	I: 8.8 (2.2); C: 8.6 (1.8)
Description of Intervention	DSME and peer leader (PL) support<br>All participants in the study and PLs attended a four-month government-sponsored DSME program named "A 7 Pasos del Control" (i.e. "Seven Steps to Achieve Control") delivered by a dietitian and diabetes educator certified by the Mexican Council of Diabetes Educators. The DSME program consisted of 16 one-hour weekly group sessions that were offered both in the morning and afternoon to increase opportunities for participation.<br>In addition, all study participants completed a comprehensive one-on-one nutrition counselling session with a dietitian during the first month of the study. Study subjects were also encouraged to participate in two 50-minute exercise sessions per week during the eight months of the study.<br>Participants in the PSEG also attended peer-support meetings facilitated by nine PLs throughout the eight months of the study. Therefore, the PL support intervention took place during the DSME period (first 16 weeks) as well as during the next four months when the PLs informally discussed the content of the DSME with subjects in the intervention group.<br>Each PL facilitated a single group of three or four participants. There were 20 peer-support meetings over eight months per group: one session every week.

	Each meeting was facilitated by the PL without the presence of health professionals.<br>Leaders were trained to start each meeting with an “icebreaker” introduction, followed by a discussion session and goal setting, as described in the Peer Leader Manual. The discussion segment was based on the DSME theme of the previous education session for the first four months, and for the remaining sessions the theme was identified by each PL according to the needs and interests of the groups. The duration of the meetings varied between 30 and 60 minutes, depending on attendance and group engagement in the discussion.
Description/definition of peer support	All peer leaders (supporters) were known diabetics with most recent HbA1C <8%. They were required to have good communication skills and were trained on basic aspects of diabetes, communication and leadership skills according to the Peer Leader Manual.<br><br>Definition: Provision of support from an individual member of the community, with experiential knowledge based on sharing similar life experiences.
Mode of peer support	Face to face
Setting/Context: Location or Primary site of delivery of intervention	Community centre
Description of Control	DSME alone
Duration of intervention and control	I: Eight months; C: Four months
Time points of outcome measures	Baseline, four- and eight-month follow-up
List of outcomes and measurement tool	A1C: high-performance liquid chromatography (Model D-10TM; Bio-Rad Laboratories, Hercules, California, United States)<br>Diabetes-related quality of life: Diabetes-related Quality of Life Questionnaire<br>Systolic and diastolic blood pressures: automatic blood pressure monitor [HEM-7220; Omron, Kyoto, Japan]<br>BMI: weight: balance [F514; Omron] and height: stadiometer [Seca 213 Mobile; Seca, Hamburg, Germany]<br>Diet: 24-hour dietary recall (R-24)<br>Physical activity: International Physical Activity Questionnaire
Outcomes mapped to Evans et al	(2) assistance in daily management,<br>(3) social and emotional support to promote disease self-management and coping with negative emotions,

Author, Year	Chan, 2014
Location (city, country)	Hong Kong
Objective	Investigated if frequent contacts through a telephone-based peer support program (Peer Support, Empowerment, and Remote Communication Linked by Information Technology [PEARL]) would improve cardiometabolic risk and health outcomes by enhancing psychological well-being and self-care in patients receiving integrated care implemented through a web-based multicomponent quality improvement program (JADE [Joint Asia Diabetes Evaluation]).
Study Design	Randomized clinical trial.
Inclusion/Exclusion Criteria	Inclusion: Chinese patients with type 2 diabetes mellitus (T2DM) aged 18 to 70 years who underwent comprehensive assessments and expressed willingness to participate<br><br>Exclusion: illiteracy; inability to communicate in Chinese; and JADE Risk Category 1 (no complication, ≤1 risk factor, and low-risk score); psychotic symptoms, thought disorders, and suicidal ideas, patients requiring psychiatric treatment
Overall n (number invited)	628
Intervention n (number invited)	312
Control n (number invited)	316
Loss to follow-up, n (%)	O: 42 (7); I: 16 (5); C: 26 (8)
Age Mean (SD)	O: 54.7 (9.3); I: 54.5 (9.9); C: 54.8 (8.6)
Female I: n (%); C: n (%)	I: 134 (43); C: 139 (44)
Male I: n (%); C: n (%)	I: 178 (57); C: 177 (56)
Diseases at Baseline	T2DM
BMI Mean (SD)	O: 26.9 (4.5); I: 26.6 (4.3); C: 27.1 (4.6)
Blood pressure Mean (SD)	O: SBP: 136 (19), DBP: 80 (11); I: SBP: 136 (19), DBP: 80 (10); C: SBP: 135 (19), DBP: 80 (11)
HbA1c Mean (SD)	O: 8.2 (1.6); I: 8.2 (1.7); C: 8.2 (1.6)
Description of Intervention	For patients assigned to the JADE + PEARL group, the nurses put them in groups of 10 and arranged a separate two-hour session when two to three groups of patients were introduced to their assigned peer supporters, each of whom was assigned 10 patients. None of the peer supporters were aware of the details of the control group. During these weekend sessions, the nurses facilitated group sharing on self-care and stress management. After exchanging telephone numbers, the peer supporters were instructed to call their assigned peers at least 12 times—initially, biweekly calls for three months, then monthly calls for three months, and then one call every other month for six months, with an anticipated 15 minutes per call. Both peer supporters and peers were encouraged to call one another ad lib.

Description/definition of peer support	Peer supporters were motivated patients with well-controlled T2DM who received 32 hours of training to become peer supporters. They were reinforced on the principles of communication and empathic listening and encouraged to share their positive experiences to assist their peers to manage diabetes on a day-to-day basis. Additionally, they were reminded of factors that could influence blood glucose level, e.g., diet, exercise, poor sleep, stress, changes in daily routines, bodyweight, medications, and concurrent illnesses, and thus the importance of self-monitoring of blood glucose. Some of them were active members of patient groups organized by lay associations or diabetes centres. All peer supporters were given a booklet on resources (eg, websites and telephone numbers of community centers, lay associations, and hospital diabetes centers and titles of self-help books) and a three-monthly checklist to document the discussion items (diet, exercise, self-monitoring of blood glucose, sick day management, foot care, emotional support, resources for information, and clinical care), duration of each call, and relevant remarks.<br><br>Definition: Provision of support for daily management, linkage to clinical care, and ongoing social and emotional support.
Mode of peer support	face to face, phone
Setting/Context: Location or Primary site of delivery of intervention	Three diabetes centers
Description of Control	Twice-weekly structured comprehensive assessments.
Duration of intervention and control	12 months
Time points of outcome measures	Baseline, 12 months post intervention
List of outcomes and measurement tool	HbA1c<br><br>Quality of life: 5-item Euroqol (EQ-5D)<br><br>Depression: 9-item Patient Health Questionnaire (PHQ-9)<br><br>Psychological distress: 21-item Depression Anxiety Stress Scale (DASS-21)<br><br>Self-efficacy: 20-item Diabetes Empowerment Scale DES-20<br><br>Diabetes Distress: Chinese 15-item Diabetes Distress Scale (CDDS-15)<br><br>Self-care: 14-item Summary for Diabetes Self-care Activities (SDSCA1-14)
Outcomes mapped to Evans et al	(2) assistance in daily management,<br>(3) social and emotional support to promote disease self-management and coping with negative emotions,

Author, Year	Debussche, 2018
Location (city, country)	Bamako, Mali
Objective	Evaluated the effectiveness of peer-led self-management education in improving glycaemic control in patients with type 2 diabetes in a low-income country.
Study Design	Randomised controlled trial.
Inclusion/Exclusion Criteria	Inclusion: between 30 and 80 years; underwent regular follow-ups and monitoring in Bamako consultation units for poorly controlled T2D (HbA1c ≥ 8%); accepted to attend all peer-led educational sessions; and agreed to have their clinical and biological measurements taken until completion of the protocol<br><br>Exclusion: Patients with type 1 diabetes and those who suffered from severe complications in the three months preceding enrolment (infections, severe renal failure, coronary events, foot lesions) or from concomitant illnesses that threatened their functional or vital prognosis
Overall n (number invited)	151
Intervention n (number invited)	76
Control n (number invited)	75
Loss to follow-up, n (%)	O: 11 (7); I: 6 (8); C: 5 (7)
Age Mean (SD)	O: 52.5 (9.8); I: 53.9 (9.8); C: 51.1 (9.6)
Female I: n (%); C: n (%)	I: 57 (75); C: 58 (77)
Male I: n (%); C: n (%)	I: 19 (25); C: 17 (23)
Diseases at Baseline	T2D
BMI Mean (SD)	O: 26.6 (5.4); I: 28.3 (5.4); C: 28.8 (5.5)
Blood pressure Mean (SD)	SBP: O: 129.9 (23.9); I: 132.8 (26.9); C:127.1 (20.1); DBP: O: 81.6 (10.6); I: 82.9 (10.5); C: 80.3 (10.6)
HbA1c Mean (SD)	O: 10.7 (1.8); I: 10.6 (1.8); C: 10.8 (1.9)
Description of Intervention	Three courses delivered in the community by trained peer educators over one year. Each course was composed of four different thematic sessions (4–10 participants) offered over a period of three months (months 1–3, 7–9, and 10–12). The initial protocol stated 1.5 hour-long sessions, however, the actual duration of sessions during the trial with peer educators was 1.5–2 hours. The themes addressed were cardiovascular risk management, food intake, exercise, and blood glucose and insulin management. The content, approach and programme of each group session were detailed in specific booklets for learners (including learners with literacy difficulties) and culturally adapted for Mali (food habits, language specificities, occupational and environment issues).<br>The peer-led structured patient education intervention drew on the 'Learning Nests' (Nids d'apprentissage) approach. Briefly, this empowerment-based

	approach, derived from socio-constructivist theory, considers the context of the illness, prevailing health practices, and the chronic dimension of the disease. It promotes patients' understanding of key concepts in their interactions with their social environment. Educational sessions were structured around five components:<br>patients' analysis of their own knowledge and practices, the recognition of individual contexts and problem-solving, individual heterogeneity as an asset for self-assessment and action, culturally-tailored educational materials, and the long-term dimension of disease management.<br>The 'Learning Nests' approach incorporated and emphasised behavioural strategies as opposed to traditional didactic teaching. These strategies include the implementation of curricula and the distribution of hand-outs designed to meet the specific needs of target populations.
Description/definition of peer support	Peer educators (PEs) were recruited from the local association of diabetic patients. The following criteria were used for selection - having diabetes, living in the locality, undergoing regular checks with a referent physician, volunteering to deliver educational sessions, and being fluent in both French and the local Bambara language. The recruited PEs attended an initial 4-day training program. They underwent two further evaluations before being actively involved in the project.<br><br>Definition: N/R
Mode of peer support	Face to face
Setting/Context: Location or Primary site of delivery of intervention	Two secondary health centres.
Description of Control	Conventional care
Duration of intervention and control	12 months
Time points of outcome measures	Baseline, 3-, 6- and 12-month follow-up.
List of outcomes and measurement tool	HbA1c: CloverA1c (Infopia Co., Ltd., South Korea)<br>Weight<br>BMI<br>Waist circumference: tape measure<br>Systolic and diastolic blood pressure<br>Anti-diabetic and anti-hypertensive treatment<br>Diabetes knowledge score: 77-open items questionnaire<br>Dietary practices: 24-hour dietary recall and a food frequency questionnaire
Outcomes mapped to Evans et al	(2) assistance in daily management,

Author, Year	Gagliardino, 2013
Location (city, country)	La Plata, Argentina
Objective	Compare clinical, metabolic and psychological outcomes in people with type 2 diabetes 1 year after attending a structured diabetes education programme implemented by professional educators versus<br>the same programme implemented by trained peers with diabetes that also provided ongoing peer support.
Study Design	Randomized control trial
Inclusion/Exclusion Criteria	Inclusion: eligible patients were 25–75 years old, followed for at least 2 years and with more than two diabetes encounters.<br>Exclusion: end stage renal disease, class III or IV cardiac failure, cancer, blindness, drug or alcohol addiction and inability to provide self-care<br><br>Inclusion: Peers recruited on the basis of their excellent diabetes control, self-motivation, communication and support skills and interest
Overall n (number invited)	198
Intervention n (number invited)	93
Control n (number invited)	105
Loss to follow-up, n (%)	NR
Age Mean (SD)	I: 62 (9); C: 60 (10)
Female I: n (%); C: n (%)	I: 50 (53); C: 53 (50)
Male I: n (%); C: n (%)	I: 43 (47); C: 52 (50)
Diseases at Baseline	T2D
BMI Mean (SD)	I: 32 (7); C: 33 (6)
Blood pressure Mean (SD)	I: SBP: 137 (28); DBP: 78 (14); C: SBP: 130 (23); DBP: 76 (14)
HbA1c Mean (SD)	I: 7.1 (1.5); C: 7.3 (1.5)
Description of Intervention	The study included two different groups. The first group is the control patient education group (control) that received the educational intervention at the Houssay Centre. The Diabetes Structured Education Courses for People with T2DM was released through trained educators to no more than ten ambulatory patients in a group setting that allowed active interaction between the educator and participants. It consisted of four weekly teaching units (90–120 min each) and a reinforcement session at 6 months. The first teaching unit included general concepts about type 2 diabetes, the symptoms of hypoglycaemia and hyperglycaemia and glucose self-monitoring, with strong emphasis on the importance of active patient participation in disease control and treatment. In the second teaching unit, the effect of obesity on insulin sensitivity and the advantages of weight reduction and of patient learning to classify and select foods according to their calorie content were discussed. The third teaching unit explained the importance of foot care and

	regular practice of physical activity, while during the fourth unit they learned the basic rules for 'sick days' and which were the examinations and laboratory tests necessary to have good diabetes care. Many illustrated educational materials were used, as well as a book provided to each patient that included the main contents of the programme. To test the diabetes knowledge of the participants, we used a multiple-choice questionnaire.<br>The second group is the peer patient education plus peer support group (peer) that received identical education plus the active participation of peers, who were integrated into the educational models and specific peer activities. The goal of the latter activities was to provide continuing psychological and behavioural support and to teach the patients how to apply in everyday life the knowledge acquired during the education course, based upon the peer's personal experience. The peer's postcourse role and activity were complementary to the formal education; for that purpose, we implemented two different activities: (a) peer education and (b) peer support. Peer education was integrated into the educational units serving as 'real world living models' for the attendee. One peer worked fulltime and was responsible for the overall management of peers; he or she received a small direct compensation for his or her teaching, supervisory and administrative role.<br>For each educational module of the course, there was a specific set of supporting activities that the educator-peer shared with the supporting peers. To test the diabetes knowledge of participants, we used the same multiple choice questionnaire mentioned earlier. Following the initial education course, peers had regular and continuing scheduled contacts with their supportees. Their contacts took the combined pre-established form of scheduled face-to-face visits or whenever a specific issue warranted, and frequent interactions by mobile telephone. The face-to-face visits among peers and their supportees were scheduled every second month. The telephone communications took place at least weekly for the first 6 months, biweekly for the next 3 months and monthly for the remaining study period. They were based on structured interviews that inquired into the patients' clinical, metabolic and psychological progress. The data requested included body weight, blood pressure, self-monitoring blood glucose (SMBG) values, psychological status, medication, meal plan and physical activity adherence and other coping mechanisms. This information was recorded and sent to the coordinator, becoming part of the patient's follow-up. In addition to these one-on-one telephone calls, we promoted monthly group calls among peers to share experiences, difficulties and alternative solutions implemented. A critical role of the peers was to provide throughout this system the psychological support that their supportees needed to cope with the day-to-day vicissitudes of diabetes self-care. Thus, more frequent interactions in person or by telephone were encouraged at times when more intensive psychological support was considered appropriate.
Description/definition of peer support	Peers were recruited on the basis of their excellent diabetes control, self-motivation, communication and support skills and interest. They were trained for 3 days using the curriculum of the health professionals Training

	Course on Diabetes Education.<br><br>Definition: A reasonable approach involving people with diabetes in the delivery of education and support needed for long-term self-management.
Mode of peer support	Face to face (group) and telephone (individuals)
Setting/Context: Location or Primary site of delivery of intervention	Houssay Center in La Plata
Description of Control	4-week structured diabetes education course delivered by professional educators
Duration of intervention and control	12 months
Time points of outcome measures	Baseline, 1 month, 6 months, 12 months
List of outcomes and measurement tool	Changes in diabetes classical symptoms<br>BMI<br>Blood pressure<br>FBG<br>A1c<br>Cholesterol<br>HDL<br>Triglycerides<br>Diabetes distress questionnaire<br>Well-being survey
Outcomes mapped to Evans et al	(2) assistance in daily management,<br>(3) social and emotional support to promote disease self-management and coping with negative emotions,

Author, Year	Ghasemi, 2019
Location (city, country)	Isfahan, Iran
Objective	Assessed the effect of peer education on the quality of life (QOL) of elderly people with diabetes.
Study Design	Randomized clinical trial.
Inclusion/Exclusion Criteria	Inclusion: >65 years; medical record in the healthcare centers; willing to participate in the study; literate; received insulin or glucose- lowering medications; no long- term side effects of diabetes<br><br>Exclusion: absent from more than two sessions; and those who were not willing to participate
Overall n (number invited)	56
Intervention n (number invited)	28
Control n (number invited)	28
Loss to follow-up, n (%)	O: 12 (21); I: 7 (25); C: 5 (18)
Age Mean (SD)	NR
Female I: n (%); C: n (%)	I: 13 (55); C: 14 (66)
Male I: n (%); C: n (%)	I: 10 (45); C: 7 (34)
Diseases at Baseline	Diabetes
BMI Mean (SD)	NR
Blood pressure Mean (SD)	NR
HbA1c Mean (SD)	NR
Description of Intervention	Individuals who were interested and highly motivated were educated by peers. Both groups received eight sessions of training, each lasting 30–45 minutes. The content of the training sessions consisted of educational information regarding self- care, including exercise, diet, and skin care, and elements regarding QOL and common worries related to diabetes using lectures, discussions, and question and answer. The content of the sessions were devised under the supervision of the researcher and a diabetes specialist. The educational sessions in both groups were held at the health centers (Imam Ali and Ghaedi centers) on Tuesdays and Wednesdays at different times (at 9–10 a.m. for the peer- trained group and 10–11 a.m. for the researcher- trained group).
Description/definition of peer support	Recruitment and details of peers was not fully described. The authors describe the peers to be interested and highly motivated. Peers who were training their group were further supervised by a researcher.<br><br>Definition: Peer education is a process in which motivated and trained individuals are responsible for organized education of their peers that aims to raise awareness and improve skills in the target individuals and enable them

	to accept their responsibility in protecting their health.
Mode of peer support	Face to face
Setting/Context: Location or Primary site of delivery of intervention	Health centers (Imam Ali and Ghaedi centers)
Description of Control	Received training by the researcher.
Duration of intervention and control	8 sessions
Time points of outcome measures	baseline, immediately after the intervention, one month after the intervention
List of outcomes and measurement tool	Quality of life: Diabetes Quality- of- Life (DQOL)
Outcomes mapped to Evans et al	(3) social and emotional support to promote disease self-management and coping with negative emotions,

Author, Year	Hernandez, 2021
Location (city, country)	Takeo province, Cambodia.
Objective	Evaluated a programme in Cambodia, financed by a revolving drug fund, which utilizes Peer Educators to manage diabetes and hypertension in the community. Assessed clinical outcomes and retention in the programme.
Study Design	Retrospective exploratory analysis.
Inclusion/Exclusion Criteria	Inclusion: aged >18 years living in the largely rural Takeo province in Cambodia, who were screened and diagnosed with diabetes or hypertension; FBG ≥126 mg/dl or post-prandial blood glucose (PPBG) ≥180 mg/dl and hypertension as systolic ≥140 mmHg and diastolic ≥90 mmHg.<br><br>Exclusion: NR
Overall n (number invited)	6677
Intervention n (number invited)	N/R
Control n (number invited)	N/A
Loss to follow-up, n (%)	Attrition 1st year: 44.1% (SD 10.3)<br>Attrition 2nd year: 38.3% (5.8)
Age Mean (SD)	55.7 (12.8)
Female I: n (%); C: n (%)	4479 (67.1)
Male I: n (%); C: n (%)	2198 (32.9)
Diseases at Baseline	Diabetes<br>Hypertension
BMI Mean (SD)	22.8 (3.8)
Blood pressure Mean (SD)	NR
HbA1c Mean (SD)	NR
Description of Intervention	MoPoTsyo used a community-centred approach to long-term care for diabetes and hypertension management. Peer educators (PEs), patients selected for their motivation, were trained to screen and manage these conditions in their local village and health centre catchment area. The role of PEs also includes arranging village health meetings to discuss lifestyle measures and diet. The programme itself provided laboratory services and operated a revolving drug fund (RDF) for vetted, reliable and lower-priced drugs supplied to contracted pharmacies.<br>However, training of PEs is still undertaken by MoPoTsyo with a view to transitioning this to the care of national services in the coming 5 years.
Description/definition of peer support	Peer Educators (PEs) were patients with diabetes and/or hypertension selected for their motivation who screened and initiated management of community members in their local villages, Training of PEs was undertaken by MoPoTsyo (Cambodian NGO).

	Definition: N/R
Mode of peer support	Face to face.
Setting/Context: Location or Primary site of delivery of intervention	N/R
Description of Control	N/A
Duration of intervention and control	2.3 (1.9) years
Time points of outcome measures	Baseline<br>Time to follow-up was calculated as the date of follow-up visit minus enrolment date, in years to the nearest 6 months.
List of outcomes and measurement tool	Height: height measurement stick<br><br>Weight: generic scales<br><br>BMI<br><br>FBG or PPBG: 2007 Roche Accu-Chek and ACON On Call Plus machine (used from Dec 1st onwards)<br><br>HbA1c: SIEMENS DCA<br><br>BP: Automatic Citizen CH-432B machine, AS-35B, ALP-K2 and Nissei SL 1902<br><br>The population in the program enrolled with hypertension or diabetes who were controlled over time<br><br>loss to follow-up of participants enrolled in the program.
Outcomes mapped to Evans et al	(2) assistance in daily management,

Author, Year	Ju, 2018
Location (city, country)	Nanjing, China
Objective	Investigated whether peer support would reduce diabetes distress and improve glycaemic control when added to usual diabetes education among adults with Type 2 diabetes in China.
Study Design	Cluster randomized controlled trial.
Inclusion/Exclusion Criteria	Inclusion: Type 2 diabetes according to the WHO criteria; were aged ≥18 years and participated voluntarily<br><br>Exclusion: unstable conditions (e.g. unstable angina pectoris, blood pressure >200/100 mmHg, coexisting infection); mental illness (e.g. substance misuse, schizoaffective disorder, bipolar disorder) or disturbance likely to compromise participation; carcinoma treated with surgery, radiotherapy or chemotherapy in the previous six months; or participation in other research likely to compromise or complicate participation in the study
Overall n (number invited)	400
Intervention n (number invited)	200
Control n (number invited)	200
Loss to follow-up, n (%)	O: 95 (24); I: 58 (29); C: 37 (19)
Age Mean (SD)	O: 68 (NR); I: 67.8 (7.4); C: 68.6 (8.0)
Female I: n (%); C: n (%)	I: 129 (65); C: 135 (68)
Male I: n (%); C: n (%)	I: 71 (35); C: 65 (32)
Diseases at Baseline	Type 2 diabetes
BMI Mean (SD)	NR
Blood pressure Mean (SD)	NR
HbA1c Mean (SD)	I: 6.8 (1.5); C: 6.7 (1.4)
Description of Intervention	Peer leaders guided participants to carry out activities with the help of community health centres or medical volunteers. The activities included themed and non-themed activities.<br>Themed activities reviewed diabetes knowledge and skills at least once a month. An important part of this self-management support was teaching and reinforcing self-management skills. Guided by peer leaders, participants discussed and shared a variety of skills, including healthy meal planning, food preparation, blood glucose monitoring, medication management and physical activities. Peer leaders encouraged participants to communicate and share experience with each other. The leaders also worked with participants to apply knowledge and skills in practice, such as setting or achieving goals, solving problems and overcoming barriers.<br>Non-themed activities included informal communication among participants through home visits, telephone, e-mails and so on. These were facilitated by

	the peer support with usual education being organized through community health centres serving individual communities. Many participants knew each other and had frequent occasion to meet each other informally within their communities; thus, there was a variety of ways to increase knowledge about diabetes and self-management for peers, such as during tai chi and openair fitness dancing sessions.
Description/definition of peer support	Peer leaders were chosen based on residence, demographics and other characteristics, including interpersonal skills evident in interviews, time available and willingness to cooperate as part of a team and follow study protocols. Peer leaders guided participants to carry out activities with the help of community health centres or medical volunteers.<br><br>Definition: The provision of support from an individual with experiential knowledge based on sharing of similar life experiences. Guided participants to carry out activities with the help of community health centres or medical volunteers.
Mode of peer support	Face to face, phone, e-mail
Setting/Context: Location or Primary site of delivery of intervention	Eight community health centres
Description of Control	Usual education which consisted of 2 h each month of focused diabetes education. A variety of diabetes self-management interventionists including physicians, certified diabetes educators, dieticians, psychologists and podiatric nurses led individual sessions. Participants also used other resources such as newspapers, networks or other medical institutions to gain information about diabetes self-management.
Duration of intervention and control	12 months
Time points of outcome measures	Baseline, 12 months post intervention.
List of outcomes and measurement tool	Diabetes Distress: Diabetes Distress Scale<br><br>fasting plasma glucose (FPG), 2-h<br><br>postprandial glucose (PPG)<br><br>HbA1c
Outcomes mapped to Evans et al	(2) assistance in daily management,<br>(3) social and emotional support to promote disease self-management and coping with negative emotions,

Author, Year	Khan, 2018
Location (city, country)	Dhaka, Bangladesh
Objective	Compared the effectiveness of a diabetes education program guided by health professionals versus peers in improving diabetes care among people with type 2 diabetes.
Study Design	Quasi-experimental design (intervention without randomization)
Inclusion/Exclusion Criteria	Inclusion (patients): Patients with type 2 diabetes with HbA1C > 8% residing in Dhaka city from the Outpatient Department (OPD) of BIRDEM (the Tertiary Hospital of Diabetic Association of Bangladesh).<br><br>Inclusion (health professionals): Four of ten female medical graduates, practicing in the city of Dhaka, with more than four years of experience in diabetes-management education, were selected as professionals and they were provided diabetes education in the local language<br><br>Inclusion (peer educators): had diabetes at least for five years; age >40 years; HbA1c <7%; graduation in education; committed to training and willing to spend sufficient time; enthusiastic to be peer educators; and residing in Dhaka city<br><br>Exclusion: Patients with other medical complications or those, who were unable to answer a short list of simple questions (sociodemographic information, such as name, address, disease complications, etc.)
Overall n (number invited)	133
Intervention n (number invited)	Peer educator guided group: 66; health professional guided group: 67
Control n (number invited)	N/A
Loss to follow-up, n (%)	O: 9 (7); I: 1 (2), 8 (12)
Age Mean (SD)	I: 54.1 (9.4), 52.9 (11.4)
Female I: n (%); C: n (%)	I: 18 (27.7), 26 (44.07)
Male I: n (%); C: n (%)	I: 47 (72.3), 33 (55.93)
Diseases at Baseline	Type 2 diabetes
BMI Mean (SD)	I: 25.29 (3.05), 24.76 (5.9)
Blood pressure Mean (SD)	I: SBP 140.77 (17.5), DBP 85.19 (9.2), SBP 134.69 (16.6), DBP 82.65 (10.6)
HbA1c Mean (SD)	I: 9.5 (2.1), 9.5 (1.7)
Description of Intervention	Sixty-seven patients led by the health professionals were divided to four groups, and each group was directed by a professional. Sixty-six patients guided by peer educators were also divided to four groups, and each group was led by two peer educators.<br>All the patients (both professionals and peer educator groups) attended a two-hour diabetes education program once at the time of their enrollment,

	following a predesigned curriculum. The education program was followed by face-to-face and group discussions (using leaflets, a flip-chart, and posters) for any problems they faced, thereby allowing them to freely discuss general management of diabetes.<br>After 12 weeks, changes in knowledge, self-care activities, glycemic status, weight, and blood pressure among patients were observed following the information derived from the same questionnaire.
Description/definition of peer support	Peer educators were diabetics for at least 5 years with the following characteristics – age>40yrs, HbA1C <7%, graduates, committed to training and willing to spend sufficient time, enthusiastic to be peer educators and residing in Dhaka city. They underwent a three-day training program with pre and post-training assessments.<br><br>Definition: People with diabetes could be chosen to educate other patients regarding diabetes, known as peer support.
Mode of peer support	Face to face and group discussions
Setting/Context: Location or Primary site of delivery of intervention	Outpatient Department (OPD) of BIRDEM (the Tertiary Hospital of Diabetic Association of Bangladesh)
Description of Control	N/A
Duration of intervention and control	12 weeks
Time points of outcome measures	Baseline and 12 weeks follow up
List of outcomes and measurement tool	HbA1C<br><br>Fasting blood glucose<br><br>Weight<br><br>BMI<br><br>blood pressure<br><br>knowledge level: knowledge-assessment questionnaires<br><br>lifestyle changes
Outcomes mapped to Evans et al	(2) assistance in daily management,<br>(3) social and emotional support to promote disease self-management and coping with negative emotions,

Author, Year	Khetan, 2019
Location (city, country)	Dalkhola, India
Objective	Tested the hypothesis that utilizing CHWs to manage hypertension, diabetes and smoking in an integrated manner would lead to improved control of these conditions.
Study Design	Cluster randomized controlled trial.
Inclusion/Exclusion Criteria	Inclusion: age 35-70 years and permanent residence in the area allotted to the given CHW; of the screened individuals, those with ≥ 1 cardiovascular risk factor (either one of hypertension [blood pressure ≥ 140/90 mm Hg on two separate days or on antihypertensive medication], diabetes [fasting blood glucose (FBG) ≥126 mg/dl on two separate days or on antihyperglycemic medication], or current daily smoker [self-reported])<br><br>Exclusion: bed bound; deemed unable to participate in the intervention due to significant disabilities such as deafness, blindness, or intellectual disability; and had stayed <6 months in the study area before the screening date.
Overall n (number invited)	1242
Intervention n (number invited)	736
Control n (number invited)	506
Loss to follow-up, n (%)	O: 248 (20); 118 (16); C: 130 (26)
Age Mean (SD)	I: 52.1 (9.6); C: 51.7 (9.8)
Female I: n (%); C: n (%)	I: 296 (40); C: 185 (36)
Male I: n (%); C: n (%)	I: 440 (60); C: 321 (64)
Diseases at Baseline	Hypertension<br>Diabetes
BMI Mean (SD)	NR
Blood pressure Mean (SD)	I: SBP: 154.9; C: SBP: 159.5
HbA1c Mean (SD)	NR
Description of Intervention	Once patients were enrolled at the end of the second screening visit, CHWs provided home based counseling to people with hypertension. This usually lasted for around an hour, and consisted of a behavior change strategy focused on modifying the individual's lifestyle (diet and physical activity), improving health care seeking behavior, and addressing barriers to medication adherence. Importantly, in addition to lifestyle modifications, CHWs specifically focused on encouraging physician visits, medication purchase, and medication adherence. The communication was conducted in the native language of the participant. CHWs had a flipbook aid that summarized these strategies and provided a template for discussion. As many of the patients were illiterate, the patient facing side of the flipbook only had pictures, with all textual information conveyed verbally.

	Involvement of family members was encouraged. CHWs visited patients with hypertension every two months till the end of the study. At follow-up visits, CHWs had a template to use for their encounter, which was focused on reinforcing previous recommendations, understanding barriers to behavior change, behavior change communication, and problem solving. Each CHW had a paper diary in which they recorded details of their patient encounters, in a predefined format.<br>Six months after the start of the hypertension intervention, CHWs underwent training for diabetes and started visiting patients with diabetes. These visits followed a similar format and frequency as hypertension, and a separate flipbook was provided for diabetes counseling.<br>Two months after the start of the diabetes intervention, CHWs received training for the smoking intervention and started visiting patients who smoked, aided by a flipbook. However, the frequency and nature of visits for smoking were customized depending on whether the participant was contemplative or pre-contemplative about quitting smoking.<br>Once all the three interventions were underway, CHWs continued to follow all participants under their care until the end of the study. For a patient with multiple risk factors (e.g., hypertension and diabetes), CHW visits at the start of the study focused on hypertension, and after undergoing diabetes training at the 6-month mark, CHWs also began to counsel the patient on diabetes (while continuing the hypertension intervention).
Description/definition of peer support	Community health workers (CHWs) were recruited for the study and were not previously a part of the health system. The inclusion criteria do become a CHW included being a female resident of the study area for at least the past two years, between 18 and 45 years of age, having a tenth-grade level of education and possessing spoken and written knowledge of the local language. They received staggered training focused on hypertension, followed by diabetes, and then smoking. Training for each risk factor was delivered over 1 to 2 weeks (3 h/day). All CHWs were retained from the start to the end of the intervention, with zero attrition.<br><br>Definition: N/R
Mode of peer support	Face to face.
Setting/Context: Location or Primary site of delivery of intervention	Community
Description of Control	Received a handout at the end of the screening process, that explained to them their respective risk factor(s) (hypertension, diabetes, or smoking). They also received brief verbal advice regarding the same from the field worker. After screening, participants in the control group had one visit at the end of the first year of the intervention and a final visit at the end of the study. There was no other contact between control group participants and the study.
Duration of intervention and	2 years

control	
Time points of outcome measures	Baseline, 2 years post intervention.
List of outcomes and measurement tool	SBP: automated blood pressure monitors (Omron HEM 8711 [Omron Automation Pvt. Ltd., Gurgaon, India])<br><br>FBG: glucometers (Accu-chek Performa Nano [Roche Diabetes Care India Pvt. Ltd, Mumbai India])<br><br>Mean number of daily cigarettes/bidis smoked<br><br>DBP: automated blood pressure monitors (Omron HEM 8711 [Omron Automation Pvt. Ltd., Gurgaon, India])<br><br>Control rates of hypertension and diabetes<br><br>Proportion of participants with diabetes who were on a statin or aspirin<br><br>Proportion of participants with hypertension and tobacco use on a statin<br><br>Proportion of tobacco users who quit smoking<br><br>Mean reduction in weight and waist circumference for those who were overweight or had increased waist size at baseline, respectively
Outcomes mapped to Evans et al	(2) assistance in daily management,

Author, Year	Latina, 2020
Location (city, country)	Grenada
Objective	Tested the effectiveness of peer support strategy for CV risk reduction in the island of Grenada, a low-middle income country (LMIC).
Study Design	Randomized controlled trial.
Inclusion/Exclusion Criteria	Inclusion: 18-85 years of age with at least two CV risk factors, which included: elevated blood pressure (systolic ≥120 mm Hg and/or diastolic ≥80 mm Hg), overweight or obese (BMI ≥25 kg/m^2; waist circumference >40 inches for men, >35 inches for women), elevated blood glucose level (random glucose ≥140 mg/dL), low level of physical activity (<150 minutes of at least moderate physical activity/week), low fruit and vegetable intake (<2 servings/day), hyperglycemia (generously defined as fasting glucose >100 mg/dL), dyslipidemia (total cholesterol ≥250 mg/dL or LDL-C ≥130 mg/dL), or current smoking.<br><br>Exclusion: NR
Overall n (number invited)	402
Intervention n (number invited)	206
Control n (number invited)	196
Loss to follow-up, n (%)	O: 34 (8); I: 13 (6); C: 21 (11)
Age Mean (SD)	O: 51.4 (14.5); I: 51.1 (14.2): C: 51.8 (14.9)
Female I: n (%); C: n (%)	I: 134 (65); C: 131 (67)
Male I: n (%); C: n (%)	I: 72 (35); C: 65 (33)
Diseases at Baseline	Diabetes<br>Hypertension<br>Obesity
BMI Mean (SD)	NR
Blood pressure Mean (SD)	NR
HbA1c Mean (SD)	NR
Description of Intervention	Participants were instructed how to measure their weight and blood pressure accurately, and scales as well as blood pressure machines were distributed at community health centers in each parish for self-monitoring. The intervention group was organized into groups of 8–12 individuals in their local parish. The peer leaders underwent an additional three-hour training session on leadership and communication skills in addition to the relevant healthy behavior promotion.<br>The peer group meetings were planned to meet monthly for one year. The peer group leaders were educated using evidence-based guidelines, and encouraged to promote 150 minutes weekly of physical activity, consumption of at least five fruits and vegetables daily, smoking cessation, and blood

	pressure management. Peer leaders were provided topics to discuss at the monthly meetings, such as low salt diet and hypertension, diabetes prevention, coping strategies for stress, and smoking cessation; along a blood pressure machine with two different cuff sizes. Leaders were able to adapt themes for each meeting to their particular interest. The Project administrator in Grenada made routine visits to each group to encourage and monitor the attendance, and to receive feedback from the group leader. Although group leaders were provided attendance sheets, this information was not systematically collected because of different issues.
Description/definition of peer support	A Peer leader was a motivated community lay-person. They underwent an additional three-hour training session on leadership and communication skills in addition to the relevant healthy behaviour promotion.<br><br>Definition: Peer support is a low-cost intervention and therefore decrease CV risk.
Mode of peer support	Face to face.
Setting/Context: Location or Primary site of delivery of intervention	5 Local parishes
Description of Control	Received the series of educational lectures at the time of enrollment, followed by self-management for one year.
Duration of intervention and control	12 months
Time points of outcome measures	Baseline and at 12 months after completion of the intervention.
List of outcomes and measurement tool	CV health: Fuster-BEWAT score (FBS)<br><br>Quality of life: SF-36 survey score (SF36)
Outcomes mapped to Evans et al	(2) assistance in daily management,<br>(3) social and emotional support to promote disease self-management and coping with negative emotions,

Author, Year	Liu, 2020
Location (city, country)	Shanghai, China
Objective	Development, initial evaluation, and community expansion of peer support (PS) for populations with diabetes staged in the context of the Shanghai Integration Model integrating primary and specialty care for diabetes.
Study Design	Cohort
Inclusion/Exclusion Criteria	Inclusion: Across the Community Health Centers (CHCs) participants were recruited through announcements and posters in CHCs; oral invitations from GPs during outpatient visits; recommendations by peer leaders (PLs) living in the same community; recommendations by Community Self-Management Groups (CSMGs) leaders or staff working in residential committees; or through quarterly telephone or in-person assessments mandated by government policies for all with diabetes and other chronic diseases<br><br>Exclusion: NR
Overall n (number invited)	1284
Intervention n (number invited)	NA
Control n (number invited)	NA
Loss to follow-up, n (%)	179 (14)
Age Mean (SD)	Mean (95% CI): 68.00 (67.59–68.41)
Female I: n (%); C: n (%)	O: 732 (57)
Male I: n (%); C: n (%)	O: 552 (43)
Diseases at Baseline	Diabetes<br>Mean PHQ depression:<br>Minimal (score = 0–4): 74.2%<br>Mild (score = 5–9): 18.7%<br>Moderate (score = 10–14): 4.1%<br>Moderately severe (score = 15–19): 2.5%<br>Severe (score = 20–27): 0.5%
BMI Mean (SD)	Mean (95% CI): 25.08 (24.88–25.27)
Blood pressure Mean (SD)	Mean (95% CI): SBP: 135.94 (135.04–136.84), DBP: 78.41 (77.94–78.89)
HbA1c Mean (SD)	Mean (95% CI): 7.52 (7.45–7.60)
Description of Intervention	Monthly diabetes education classes and activities, led by CHC staff or co-led with PLs.<br>Informal Community Activities to Encourage Healthy Lifestyles and Diabetes Management.<br>Informal Community Activities to Promote a Sense of Community and Support Among Those with Diabetes.<br>WeChat among PLs and participants.<br>Individual follow up or meetings with those who needed or requested extra

	support.<br>PL interactions with families. Encouraged spouses to attend group meetings and activities. PLs conducted home visits to help family members understand the importance of insulin therapy for their loved ones with diabetes<br>Collaborations with other resources within neighborhoods.
Description/definition of peer support	Majority of the peer leaders (PLs) were individuals living with diabetes who were recruited based on existing relationships with people in the community and trained with knowledge and skills to help patients make the transition from discussing problems to taking action using a “Diabetes Action Plan” as a framework.<br><br>Definition: N/R
Mode of peer support	Face to face, phone
Setting/Context: Location or Primary site of delivery of intervention	Community Health Centers
Description of Control	N/A
Duration of intervention and control	12 months
Time points of outcome measures	Baseline, 12 months post intervention
List of outcomes and measurement tool	HbA1c<br>BMI<br>SBP<br>DBP<br>TC<br>TG<br>HDLc<br>LDLc<br>Depression: PHQ-8<br>Quality of life: EQ-5D<br>Diabetes specific distress: Diabetes Distress Scale
Outcomes mapped to Evans et al	(2) assistance in daily management,<br>(3) social and emotional support to promote disease self-management and coping with negative emotions,

Author, Year	Mwakalinga, 2021
Location (city, country)	Malawi
Objective	Assessed and evaluated the Kamuzu Central Hospital (KCH) diabetic peer support program's (DPSP) impact four years after its establishment by assessing knowledge, self-efficacy and behaviours of DPSP members compared to non-members.
Study Design	Cross-sectional descriptive study.
Inclusion/Exclusion Criteria	Inclusion: diagnosis of diabetes for at least one year or members of diabetic peer support program (DPSP) for one year or more.<br><br>Exclusion: NR
Overall n (number invited)	176 (DPSP: 98; Non-Member: 78)
Intervention n (number invited)	N/A
Control n (number invited)	N/A
Loss to follow-up, n (%)	0
Age Mean (SD)	DPSP: 54.04 (13.11); Non-Member: 47.73 (13.87)
Female I: n (%); C: n (%)	DPSP: 60 (61); Non-Member: 45 (58)
Male I: n (%); C: n (%)	DPSP: 38 (39); Non-Member: 33 (42)
Diseases at Baseline	diabetes
BMI Mean (SD)	NR
Blood pressure Mean (SD)	NR
HbA1c Mean (SD)	NR
Description of Intervention	N/A
Description/definition of peer support	Study did not specify characteristics of the peers. They were trained using support materials developed by the Peers for Progress organization.<br><br>Definition: N/R
Mode of peer support	Face to face
Setting/Context: Location or Primary site of delivery of intervention	Kamuzu Central Hospital
Description of Control	N/A
Duration of intervention and control	Four years
Time points of outcome measures	Four years after DPSP's establishment
List of outcomes and	knowledge: Diabetes Knowledge Test (DKT)

measurement tool	self-efficacy and behaviours: Diabetes Attitude Scale (DAS-3) and the Diabetes Empowerment Scale (DES)
Outcomes mapped to Evans et al	(2) assistance in daily management,<br>(3) social and emotional support to promote disease self-management and coping with negative emotions,

Author, Year	Paz-Pacheco, 2017
Location (city, country)	San Juan, Philippines
Objective	Assessed the effectiveness of a community-based diabetes self-management education (DSME) program in improving anthropometric, biochemical, and health behavior outcomes among persons with diabetes.
Study Design	Prospective, education-intervention trial / RCT
Inclusion/Exclusion Criteria	Inclusion: recruited by announcement by village health workers (for those already known to have type 2 diabetes) or after they have been newly identified to have type 2 diabetes in the earlier prevalence study<br><br>Exclusion: NR
Overall n (number invited)	155
Intervention n (number invited)	85
Control n (number invited)	70
Loss to follow-up, n (%)	O: 31 (20); I: 13 (15); C: 18 (26)
Age Mean (SD)	O: 57.1 (11.5); I: 57.6 (11.5); C: 56.5 (11.7)
Female I: n (%); C: n (%)	I: 60 (71); C: 49 (70)
Male I: n (%); C: n (%)	I: 25 (29); C: 21 (30)
Diseases at Baseline	Diabetes
BMI Mean (SD)	I: 23.7 (3.8); C: 24.2 (4.4)
Blood pressure Mean (SD)	I: SBP: 130 (31), DBP: 79.01 (12.03); C: SBP: 131 (30), DBP: 78.36 (10.33)
HbA1c Mean (SD)	I: 6.35 (3.95); C: 7.25 (3.7)
Description of Intervention	Participants in both groups were given oral advice on diet, exercise, foot care, and medication compliance on each follow-up visit. The participants in the intervention group additionally received DSME.<br>There were eight modules in the DSME program: overview of diabetes mellitus; diabetes and exercise; diabetes and diet; pharmacologic treatment of diabetes; insulin use; acute complications of diabetes; microvascular and macrovascular complications of diabetes; and foot care.<br>These modules were delivered by peer educators with visual aids followed by group discussions.<br>The teaching sessions were held in the village health centers, with six to 15 participants in attendance in each session. Two modules were taught per session. Each of the four weekly sessions lasted for about one hour.
Description/definition of peer support	Volunteer peer educators were recruited among the participants (known diabetics). They attended a two-day workshop during which they received a course manual that described both the course content and process on how to teach them.<br><br>Definition: 'Community catalysts,' to promote a healthy lifestyle among

	people with diabetes in the community.
Mode of peer support	Face to face
Setting/Context: Location or Primary site of delivery of intervention	Village health centers.
Description of Control	Standard care
Duration of intervention and control	4 weeks
Time points of outcome measures	Baseline and after three and six months.
List of outcomes and measurement tool	Body mass index<br><br>Waist–hip ratio<br><br>SBP<br><br>DBP<br><br>HbA1c<br><br>Fasting blood sugar<br><br>Cholesterol<br><br>LDL<br><br>HDL<br><br>Triglycerides<br><br>behavioral measures: Regular exercise, Smoking, Foot examination, Alcohol intake, Illicit drug use
Outcomes mapped to Evans et al	(2) assistance in daily management

Author, Year	Peimani, 2018
Location (city, country)	Tehran, Iran
Objective	Assessed the effectiveness of a peer support intervention, in which patients with T2DM were provided ongoing self-management support by trained peers with diabetes directed at improving self-care behaviors, self-efficacy and life quality.
Study Design	Randomized controlled trial.
Inclusion/Exclusion Criteria	Inclusion: aged between 25 and 75 years; diagnosed with T2DM for more than 12 months; planned to continue receiving care at this clinic for the next six months; and had access to a telephone.<br><br>Exclusion: current debilitating medical or related condition (e.g. severe mental illness, end-stage cancer, blindness, and inability to provide self-care).
Overall n (number invited)	200
Intervention n (number invited)	100
Control n (number invited)	100
Loss to follow-up, n (%)	0
Age Mean (SD)	I: 59.0 (11.3); C: 58.8 (11.7)
Female I: n (%); C: n (%)	I: 47 (47); C: 49 (49)
Male I: n (%); C: n (%)	I: 53 (53); C: 51 (51)
Diseases at Baseline	T2DM
BMI Mean (SD)	I: 28.11 (5.29); C: 28.35 (4.80)
Blood pressure Mean (SD)	NR
HbA1c Mean (SD)	I: 7.29 (1.33); C: 7.47 (1.49)
Description of Intervention	The intervention group was subdivided into ten groups, each comprising ten persons and randomly paired with one of the trained peers for the next six months. Each peer was asked to facilitate one group meeting each month during the same period. Participants were reminded by their peers to attend all of the scheduled monthly group sessions.<br>In each session, participants discussed and shared their experiences and challenges of diabetes management with each other (two by two) and with the group members and supported each other to set and achieve their goals. Peer encouraged all group members to speak and actively participate in discussions and did not allow participants go outside the mainstream of discussions.<br>Peers were advised that group meetings should last up to two hours.<br>Peers had regular scheduled telephone contacts weekly with their 10 patients. The aim of these contacts, which were mostly unstructured and individualized, was to provide continuing social, emotional and behavioral support and to help the patients on how to apply their diabetes knowledge in

	everyday life based on the peer's personal experience and to discuss the practical issues arising from living with diabetes.
Description/definition of peer support	Peers who were known diabetics were nominated by physicians and diabetes educator nurses in the clinic based on their diabetes control (HbA1C <8%), good interpersonal skills, self motivation and good active and non-judgemental listening skills. The peers were also to be able to read and write and had to attend a three-day course.<br><br>Definition: Peer support programs are a promising way to boost social and emotional support, help patients in day-to-day management of living with diabetes and promote linkages to clinical care.
Mode of peer support	Face to face and phone
Setting/Context: Location or Primary site of delivery of intervention	University specialty clinic.
Description of Control	Received usual clinic education.
Duration of intervention and control	Six months
Time points of outcome measures	Baseline, six months/post intervention
List of outcomes and measurement tool	self-care activities: Diabetes Self-Management Questionnaire (DSMQ)<br><br>self-efficacy: Diabetes Management Self-Efficacy Scale (DMSES)<br><br>health-related quality of life: Swedish Health-Related Quality of Life Survey (SWED-QUAL)<br><br>HbA1c<br><br>BMI
Outcomes mapped to Evans et al	(2) assistance in daily management,<br>(3) social and emotional support to promote disease self-management and coping with negative emotions,

Author, Year	Rao, 2020
Location (city, country)	Cambodia
Objective	Quantified MoPoTsyo participant utilization of each program service and defined the relationship between each program service and glycemic control.
Study Design	Retrospective cohort study.
Inclusion/Exclusion Criteria	Inclusion: NR<br><br>Exclusion: if baseline enrollment assessment was not done; one or more baseline characteristics were missing or out of reasonable range (e.g., glucose level of zero assumed to be measurement or data entry error); hypertension only and not diabetes; less than 90 days of follow-up data was available since medication adherence could not be accurately calculated in these instances; baseline enrollment assessment was completed more than 90 days before or after first use of MoPoTsyo services since these data do not reflect characteristics before program participation; participant had not used any MoPoTsyo services for 12 months, which is MoPoTsyo's definition of an inactive member.
Overall n (number invited)	7980
Intervention n (number invited)	N/A
Control n (number invited)	N/A
Loss to follow-up, n (%)	NR
Age Mean (SD)	O: 55 (11)
Female I: n (%); C: n (%)	O: 2752 (65)
Male I: n (%); C: n (%)	O: 1458 (35)
Diseases at Baseline	Diabetes, hypertension
BMI Mean (SD)	NR
Blood pressure Mean (SD)	NR
HbA1c Mean (SD)	NR
Description of Intervention	MoPoTsyo Patient Information Center is a Cambodian NGO established in 2004 that has trained peer educators to find and engage members of their communities with diabetes, implement educational sessions on diabetes, and visit these community members monthly to reinforce training and monitor glucose. In addition to its peer educator network, MoPoTsyo also facilitates the following services for its member patients: physician consultations; routine laboratory tests; and low-cost medications through contracted local pharmacies<br>MoPoTsyo selects peer educators among community members with diabetes based on literacy, motivation, and social aptitude. Each peer educator candidate undergoes a six-week training course developed by physicians, pharmacists, and experienced peer educators. This course aims to teach peer

	educators about the biology of diabetes as well as key components of monitoring and treatment. At the end of the training course, candidates must pass an exam in order to become qualified MoPoTsyo peer educators. Peer educators return to their communities and perform house-to-house diabetes screening. Once enrolled, participants with diabetes may attend group sessions—typically monthly—hosted by peer educators in their homes for disease monitoring (point-of-care glucose, blood pressure, and weight), self-management education, and support. Ongoing support and supervision of peer educators is provided by other peer educators in their district and MoPoTsyo staff.
Description/definition of peer support	Peer educators (PEs) were community members with diabetes selected based on literacy, motivation and social aptitude. They underwent six-week training course developed by physicians, pharmacists, and experienced peer educators.<br><br>Definition: Peer educator programs have been used to improve chronic disease management by providing educational support and linkages to care, particularly in resource-poor settings.
Mode of peer support	Face to face
Setting/Context: Location or Primary site of delivery of intervention	NR
Description of Control	N/A
Duration of intervention and control	N/A
Time points of outcome measures	Data collection was from 2006-2016.
List of outcomes and measurement tool	Fasting plasma glucose<br><br>HbA1c<br><br>Medication adherence<br><br>number of peer educator visits<br><br>Number of physician consultations<br><br>Number of laboratory tests
Outcomes mapped to Evans et al	(2) assistance in daily management,<br>(4) linkage to clinical care and community resources, and

Author, Year	Rotheram-Borus, 2012
Location (city, country)	Cape Town, South Africa
Objective	Tested the feasibility and acceptability of a mobile phone–based peer support intervention among women in resource-poor settings to self-manage their diabetes.
Study Design	Pilot study.
Inclusion/Exclusion Criteria	Inclusion: Women diagnosed with diabetes (T2DM, T1DM) receiving health services at a local health clinic were approached by their nursing sister and the project director to participate.<br><br>Exclusion: NR
Overall n (number invited)	22
Intervention n (number invited)	N/A
Control n (number invited)	N/A
Loss to follow-up, n (%)	1 (5)
Age Mean (SD)	53 (12.8)
Female I: n (%); C: n (%)	O: 22 (100)
Male I: n (%); C: n (%)	O: 0
Diseases at Baseline	diabetes
BMI Mean (SD)	O: 39.6 (12.0)
Blood pressure Mean (SD)	O: SBP: 149.0 (24.3), DBP: 88.9 (13.4)
HbA1c Mean (SD)	O: 8.1 (4.0)
Description of Intervention	The intervention had three components: a series of 12 psychoeducational group sessions that address improving one's lifestyle of eating, exercising, and abstaining from alcohol and drugs; mobile phone probes that ask about health daily; and text messaging to a buddy to support lifestyle changes.<br>Diabetes Buddies, offered a laddered system of peer support. The program paired volunteer peers to offer each other intensive, reciprocal, ongoing support via a mobile phone. Low-cost, easy-to-use, mobile phone text-messaging technology added an element of remote support for enhanced reach, effectiveness, and scalability. Two peer mentors with diabetes were identified: positive role models who had lost weight and increased exercise after their T2DM diagnosis. After they were trained in management of diabetes, support processes, and group management by the project team, peer mentors received payment and functioned as peer educators, who conducted a series of 12 drop-in informational support meetings and offered support to the Diabetes Buddy pairs.<br>Weekly sessions were held that included a sequence of identifying weekly successes; learning new information about nutrition, exercise, and disease

	self-management; problem solving in how to apply the information in daily life; managing uncomfortable emotions such as anger, anxiety, or depression; role-playing new alternative strategies for coping with stress; and sharing a meal. In addition to providing information and support, meetings included self-check of basic diabetes markers (weight, waist circumference, blood pressure). For on-time arrival, women were given pedometers to self-monitor their number of steps daily.
Description/definition of peer support	Peer mentors were positive role models who had lost weight and increased exercise after their T2DM diagnosis. They were trained in the management of diabetes, support processes and group management by the project team.<br><br>Definition: N/R
Mode of peer support	Face to face, phone
Setting/Context: Location or Primary site of delivery of intervention	Xhosa township
Description of Control	N/A
Duration of intervention and control	12 weeks
Time points of outcome measures	Baseline, three months post intervention, six months follow-up.
List of outcomes and measurement tool	Process measures included the number of voice or text messages exchanged by buddies, attendance at psychoeducational small group meetings, and perceived quality of the intervention<br><br>Intermediate measures included the number of steps taken per day and number of hours slept each night<br><br>Diabetes-specific measures included BMI, blood pressure, emotional distress: Brief Symptom Inventory, and styles of coping
Outcomes mapped to Evans et al	(1) being there,<br>(2) assistance in daily management,<br>(3) social and emotional support to promote disease self-management and coping with negative emotions,<br>(5) ongoing support because chronic disease is for the rest of one's life.

Author, Year	Sazlina, 2015
Location (city, country)	Selangor, Malaysia
Objective	Evaluated whether personalized feedback (PF) only or combined with peer support (PS) improves physical activity among older Malays with type 2 diabetes (T2DM) compared to usual care only.
Study Design	Randomized controlled trial.
Inclusion/Exclusion Criteria	Inclusion: community-dwelling Malays aged ≥60 years; diagnosed with T2DM for ≥one year; sedentary lifestyle [physical activity <150 min/week of moderate intensity]; and with follow-up care for their T2DM ≥ two visits in the last 12 months.<br><br>Exclusion: Recent adjustment in the treatment regime needing increase dose of medication in the last two months; fasting blood glucose of >13 mmol/L; presence of cognitive impairment (Elderly Cognitive Assessment Questionnaire ≤7); uncontrolled hypertension (blood pressure ≥180/100 mmHg); coronary artery syndrome; hemiparesis or hemiplegia; known advanced osteoarthritis or conditions deterring walking activity; psychiatric disorders (such as depression, anxiety, psychosis); complications of diabetes (such as proliferative retinopathy, renal impairment); uncontrolled respiratory conditions (such as asthma or chronic obstructive pulmonary disease); hearing impairment; visual impairment (visual acuity worse than 6/18 after optical correction); lives in residential homes.
Overall n (number invited)	69
Intervention n (number invited)	PF: 23, PS: 23
Control n (number invited)	23
Loss to follow-up, n (%)	O: 17 (25); I: PF: 4 (17), PS: 6 (26); C: 7 (30)
Age Mean (SD)	median (IQR): O: 64.00 (7.00); I: PF: 63.00 (8.00), PS: 64.00 (7.00); C: 63.00 (7.00)
Female I: n (%); C: n (%)	I: PF: 9 (39), PS: 11 (48); C: 12 (52)
Male I: n (%); C: n (%)	I: PF: 14 (61), PS: 12 (52); C: 11 (48)
Diseases at Baseline	T2DM
BMI Mean (SD)	median (IQR): O: 26.80 (5.50); I: PF: 26.60 (4.40), PS: 27.00 (5.50); C: 26.10 (7.00)
Blood pressure Mean (SD)	O: SBP: 138.30 (13.32), DBP: 80.00 (10.00); I: PF: SBP: 137.35 (6.42), DBP: 78.00 (17.00), PS: SBP: 138.52 (13.32), DBP: 80.00 (10.00); C: SBP: 139.04 (10.68), DBP: 80.00 (10.00)
HbA1c Mean (SD)	median (IQR): O: 8.10 (1.90); I: PF: 8.30 (1.70), PS: 8.10 (2.00); C: 8.10 (2.70)
Description of Intervention	PF and PS groups engaged in a 12-week regular unsupervised walking activity. The participants performed gradual walking activity toward the recommended 30 min/day on ≥five days in a week at moderate intensity and monitored their walking activity intensity using the Talk Test.

	Participants in the PF and PS groups received structured PF and usual diabetes care. The feedback comprised participants' physical activity patterns (based on the calculated minutes spent walking in a week each month) provided in three one-to-one sessions with the first author during monthly clinic visits. Their attending doctors at the clinic provided the usual diabetes care.<br>The participants in the PS group received support from peer mentors in addition to the PF and usual diabetes care. The peer mentors were volunteers aged ≥60 years with T2DM who lived in the same community as the participants. They motivated and provided support to the participants to walk regularly based on the feedback through three face-to-face and three telephone contacts over the 12 weeks.
Description/definition of peer support	Peer mentors were volunteers with ≥5 years of T2DM, engaged in regular physical activity, had HbA1c <8% and living in the community of the study location. Peer mentors also attended a 2-day training session.<br>Definition: Assistance in applying disease management and prevention plans in daily life, emotional and social support, linkage to clinical care and on-going support.
Mode of peer support	Face to face, phone
Setting/Context: Location or Primary site of delivery of intervention	Primary healthcare clinic.
Description of Control	Usual diabetes care.
Duration of intervention and control	12 weeks
Time points of outcome measures	Baseline, 12 weeks/post intervention, 24- and 36-weeks follow-up.
List of outcomes and measurement tool	Physical activity level: pedometer (Yamax Digi-Walker® CW 700/701, Japan) and Physical Activity Scale for the Elderly (PASE)<br>HbA1c<br>Blood pressure<br>Body composition (weight, body mass index, waist circumference, body fat percentage)<br>Lipid profiles (low-density lipoprotein cholesterol, high-density lipoprotein Cholesterol, triglycerides)<br>Cardiorespiratory fitness and balance: 6-min walk test<br>Quality of life: SF-12 Health Survey<br>Psychological wellbeing: General Health Questionnaire-12<br>Perceived social support from significant other family and friends: Multidimensional Scale for Perceived Social Support (MSPSS)<br>Self-Efficacy: Self-Efficacy for Exercise Scale
Outcomes mapped to Evans et al	(2) assistance in daily management<br>(3) social and emotional support to promote disease self-management and coping with negative emotions<br>(4) linkage to clinical care and community resources,

Author, Year	Shahsavari, 2021
Location (city, country)	Aligoudarz, Iran
Objective	Investigated the effect of peer support on the QOL among type 2 diabetic patients in deprived areas.
Study Design	Randomized clinical trial.
Inclusion/Exclusion Criteria	Inclusion: diagnosed with T2D by a specialist physician for at least six months and having a diabetes record in the clinic; >18 years of age; HbA1C higher than 7%; not participating in diabetes- related education programs in the last six months; no cognitive disorders; no physical disability, not being educated in medical sciences or related fields of study; and access to the telephone (mobile or landline).<br><br>Exclusion: death of the participants; absence in more than two education sessions; the emergence of new physical problems leading to inability to self- care; and withdrawal from cooperation.
Overall n (number invited)	80
Intervention n (number invited)	40
Control n (number invited)	40
Loss to follow-up, n (%)	0
Age Mean (SD)	I: 53.65 (14.26); C: 54.47 (12.89)
Female I: n (%); C: n (%)	I: 23 (58); C: 24 (60)
Male I: n (%); C: n (%)	I: 17 (42); C: 16 (40)
Diseases at Baseline	T2D
BMI Mean (SD)	NR
Blood pressure Mean (SD)	NR
HbA1c Mean (SD)	NR
Description of Intervention	Randomly assigned to four groups of 10 members.<br>The peer support program was conducted within three months. During this period, a two- hour education session was held in public places (mosque, coffee shop, and restaurant)/month. The content of the education included the principles of diabetes self- care. In the education sessions, the members of the group, while examining the barrier to and facilitators of implementing self- care behaviors, shared their experiences, discussed them, and provided solutions. The peers also arranged a one- hour session, group exercise, and a two- hour group food shopping program for the patients/month. In addition, the peers monitored the patients' care and supported them over the telephone. The duration of telephone calls was 15–20 min once a week. In all the sessions, the peers followed the

	predetermined topics.<br>They also submitted written details of sessions and telephone conversations to the research team. After reviewing peers' reports, the research team provided them with the necessary feedback to improve the quality of the sessions. During the study, the research team was in contact with the peers by phone. In addition to the three‐ day self‐ care education, the patients in both groups received the routine clinic care, including monthly visits by a diabetes nurse and a nutritionist.
Description/definition of peer support	Peers were those diagnosed with T2DM for at least 1 year (latest HbA1c <8%), having at least a high school diploma, had basic knowledge about diabetes, had no diabetes related chronic complications, attending all peer education sessions and being approved for their communication and interpersonal skills in the face-to-face interview session by the research team.<br><br>Definition: Peer support is delivered by similar people with diabetes in social and emotional contexts to improve patients' relationships with clinical caregivers and help them manage their daily activities of a life with diabetes.
Mode of peer support	Face to face, phone
Setting/Context: Location or Primary site of delivery of intervention	Public spaces (mosques, coffee shops or restaurants)
Description of Control	Routine clinic care.
Duration of intervention and control	Three months
Time points of outcome measures	Baseline, three months post intervention.
List of outcomes and measurement tool	QOL: Diabetes QOL Brief Clinical Inventory (DQOL‐ BCI)
Outcomes mapped to Evans et al	(3) social and emotional support to promote disease self-management and coping with negative emotions,

Author, Year	Sreedevi, 2017
Location (city, country)	Kerala, India
Objective	Studied the feasibility and effect of two low-cost interventions; yoga (Y) and peer support (P) on glycaemic and other outcomes among women with type two diabetes.
Study Design	Randomized controlled trial.
Inclusion/Exclusion Criteria	Inclusion: Women with type 2 diabetes mellitus diagnosed within the last eight years and between 30–65 years of age; HbA1c between 7–10%.<br><br>Exclusion: already practicing Yoga; meditation; women with chronic alcohol consumption and those with known diabetes complications; pregnant and lactating women; women with diabetes on alternate system of medicine Treatment; BMI >35 kg/m^2 and those with serious medical and psychiatric conditions.
Overall n (number invited)	124
Intervention n (number invited)	Y: 41, P: 42
Control n (number invited)	41
Loss to follow-up, n (%)	O: 25 (20); I: Y: 9 (22), P: 10 (24); C: 6 (15)
Age Mean (SD)	O: 51.9 (7.3); I: Y: 51.97 (7.40), P: 51.92 (8.32); C: 51.92 (6.57)
Female I: n (%); C: n (%)	I: 83 (100); C: 41 (100)
Male I: n (%); C: n (%)	I: 0; C: 0
Diseases at Baseline	Diabetes
BMI Mean (SD)	I: Y: 24.83 (3.89), P: 25.54 (4.52); C: 24.68 (4.1)
Blood pressure Mean (SD)	I: Y: SBP: 134.76 (20.63), DBP: 84 (11.43), P: SBP: 128.69 (18.07), DBP: 83.5 (9.5); C: SBP: 124.63 (15.7), DBP: 79.63 (7.64)
HbA1c Mean (SD)	I: Y: 9.5 (1.65), P: 9.4 (1.62); C: 9.6 (1.85)
Description of Intervention	Yoga: Instructor driven yoga sessions were conducted for 60 min on two days/week. On the other days the women were instructed to practice at home and maintain a daily log. The 60 min sessions consisted of 25 min-Surya namaskara-12 steps, 5–7 min deep relaxation-muscle relaxation technique, 15 min Asana or yoga postures. The postures consisted of Pavanamuktasana in the supine position, Bhujangasana and Shalabhasana in the prone position, Ardhamatsyaendrasana in the sitting position as recommended by yoga experts for diabetes. This was followed by 15 min of pranayama. A record of the food eaten, drugs consumed and exercise particulars were also maintained for two days/week considered to be representative of the entire week. This was reviewed every month. On the basis of this record the dietician determined adherence to diet based on the approximate calorie consumption in relation to the type of work done (sedentary/moderate/heavy work).<br>Peer support: The peer mentors provided support to the study participants in

	a ratio of 1:14. Each peer mentor would visit 13–14 women with diabetes. A face to face meeting with the woman with diabetes in a week for about 45–60 min on assistance in applying disease management or prevention plans in daily life, providing emotional and social support and pro active flexible ongoing support.<br>This was followed up by a telephone call in the same week. A monthly review of the activities was also undertaken by the principal investigator. During the first visit, the peer mentor collected the treatment details including drugs, diet and physical activity. In the follow up visits the peer mentor advised and monitored the woman regarding diet, exercise, timely consumption of drugs, emotional stress, symptoms, foot care etc. In the third month during the last visit the peer mentor conducted a final assessment regarding the entire process, its acceptability, difficulties and usefulness to the woman with diabetes. The woman with diabetes in the peer support group was also given a diary to record the visit, advice of the peer mentor and the changes brought about.
Description/definition of peer support	Peer mentors (PMs) were identified from the community. Eligibility criteria included having T2DM for at least 1 years with a random plasma glucose (RPG) <=250mg/dl in the last reading, someone who was generally adherent to treatment and behaviour change regimen as judged by the investigation team. Had capacity and commitment to undergo the training required, an understanding of patient confidentiality and undertaking to liaise with the concerned doctor if unanticipated problems arose during the course of the study. Peer mentors underwent a two-day training program.<br>Definition: Peer support was defined as support from an individual with experiential knowledge based on a sharing of similar life experiences or prevention plans in daily life.
Mode of peer support	Face to face and phone
Setting/Context: Location or Primary site of delivery of intervention	Rural health training centre.
Description of Control	Usual standard care including continuing oral hypoglycaemic drugs, advise on diabetic diet and exercise for at least 10 min/day to a level of 150 min/week.
Duration of intervention and control	Three months
Time points of outcome measures	Baseline, three months post intervention.
List of outcomes and measurement tool	Fasting plasma glucose: Hexokinase method; HbA1c: High performance liquid chromatography method; Quality of life: WHO QOL- BREF; Pharmacological adherence: Morisky Medication adherence scale (MMAS–8); Cholesterol: cholesterol oxidase-peroxidase method; BMI; Waist hip ratio (WHR); Blood pressure
Outcomes mapped to Evans et al	(2) assistance in daily management,

Author, Year	Taniguchi, 2017
Location (city, country)	Takeo province, Cambodia.
Objective	Evaluated outcomes of MoPoTsyo's diabetes program in Takeo Province by assessing glycemic and blood pressure outcomes for individuals diagnosed with diabetes over a 24-month follow-up period between 2007–2013.
Study Design	Retrospective cohort.
Inclusion/Exclusion Criteria	Inclusion: diabetes with or without high BP; >18 years of age; initial fasting blood glucose (FBG), at least one follow-up FBG, and an initial BP check.<br><br>Exclusion: age <18.
Overall n (number invited)	2230
Intervention n (number invited)	N/A
Control n (number invited)	N/A
Loss to follow-up, n (%)	220 (10)
Age Median (IQR)	54.5 (46.3, 61.9)
Female I: n (%); C: n (%)	1538 (69)
Male I: n (%); C: n (%)	692 (31)
Diseases at Baseline	Diabetes<br>Hypertension
BMI Median (IQR)	23.1 (20.6, 25.5)
Blood pressure Median (IQR)	SBP: 130 (118, 146); DBP: 82 (74, 91)
HbA1c Mean (SD)	NR
Description of Intervention	Peer educators screened travelled from house-to-house in each village screening for the presence of diabetes. To screen for diabetes, MoPoTsyo used urine glucose test strips. Peer educators handed out these testing strips to households and set up a follow-up appointment to check for positive results. Patients with positive urine screens were then tested using blood glucose levels to confirm the diagnosis of diabetes. MoPoTsyo used glucometers testing capillary blood for a fasting blood glucose (FBG) level greater than 126mg/dl or postprandial blood glucose (PPBG) level greater than 180 mg/dl to diagnose diabetes.<br>Blood pressure was measured using a manual sphygmomanometer. Patients with previously diagnosed diabetes, glucose testing meeting diabetes criteria, or on anti-diabetic medication, were also offered enrollment in the program. A subset of patients who enrolled into MoPoTsyo had been previously enrolled in the Médecins Sans Frontières diabetes clinic at the provincial hospital in Takeo prior to its closure in 2009 and were under treatment at time of enrollment.<br>After enrollment, the peer educators facilitated patient self-management of

	their chronic disease to improve glycemic and BP control. In order to accomplish this, peer educators met with patients individually or in a group setting monthly for the first year in the program to provide ongoing diabetes education, support, and to check FBG levels and BP. In addition, patients were encouraged to use urine glucose strips every two weeks to self-monitor their diabetes control. MoPoTsyo encouraged peer educators to create group meetings. Given the low levels of reimbursement for their work, the large coverage area and workload, the peer educators had considerable flexibility in deciding how to spend their time with the patients. Peer educators typically organized participant groups at their homes on fixed days. Some peer educators visited every village in the health center coverage area to follow up one or more patients individually.
Description/definition of peer support	Peer educators (PEs) were people with diabetes and were selected on their ability to read and write and their willingness to commit to fulfill the role. They received 6 weeks training.<br><br>Definition: Peer support programs utilize peer educators who are non-professionals to provide a variety of functions, including social and emotional support, assistance with disease management, and linkage to clinical care and community resources aiming to engage patients in self-management of their disease to sustain behaviours needed to manage diabetes and decrease the risk of diabetes complications.
Mode of peer support	Face to face.
Setting/Context: Location or Primary site of delivery of intervention	Typically, peer educator homes
Description of Control	N/A
Duration of intervention and control	24 months
Time points of outcome measures	Baseline, 6-month intervals up to 24 months.
List of outcomes and measurement tool	FBG: glucometers<br><br>BP: manual sphygmomanometer
Outcomes mapped to Evans et al	(2) assistance in daily management,

Author, Year	Thuita, 2020
Location (city, country)	Kiambu County, Kenya
Objective	Evaluated the effect of a nutrition education programme, with and without inclusion of peer-to-peer support, on metabolic syndrome (MetS) in Type 2 diabetes mellitus (T2D) patients.
Study Design	Randomized controlled trial.
Inclusion/Exclusion Criteria	Inclusion: suffering from T2D aged between 20 and 79 years, regular attendance at the Diabetes Comprehensive Care Centre (DCC); not planning to move from the study area during the study period; not pregnant; no complications such as renal failure, congestive heart failure, or stroke.<br><br>Exclusion: NR
Overall n (number invited)	153
Intervention n (number invited)	Nutrition education with peer-to-peer support (NEP): 51, nutrition education (NE): 51
Control n (number invited)	51
Loss to follow-up, n (%)	O: 10 (7); I: NEP: 3 (6), NE: 2 (4); C: 5 (10)
Age Mean (SD)	I: NEP: 57.0 (10.88); NE: 55.0 (12.34); C: 56.0 (11.97)
Female I: n (%); C: n (%)	I: NEP: 34(66.7), NE: 27(52.9); 30(58.8)
Male I: n (%); C: n (%)	I: NEP: 17(33.3), NE: 24(47.1); 21(41.2)
Diseases at Baseline	T2D<br>Foot disease<br>Eye problem<br>Kidney problem<br>Neuropathy<br>Arthritis
BMI Mean (SD)	I: NEP: 27.64 (5.72), NE: 26.34 (4.16); C: 27.11 (4.04)
Blood pressure Mean (SD)	SBP: I: NEP: 145.33 (21.33), NE: 146.04 (19.50); C: 139.98 (18.66); DBP: I: NEP: 87.88 (10.37), NE: 90.69 (8.79); C: 88.12 (9.15)
HbA1c Mean (SD)	I: NEP: 8.81 (1.94), NE: 8.37 (1.81); C: 8.28 (1.81)
Description of Intervention	Before random assignment to control or intervention groups, all study participants received standard education. After the standard education, the intervention groups (NEP and NE) underwent a nutrition education programme for 8 weeks, which also covered the importance of physical activity (NE group). In addition, the NEP group was trained on peer-to-peer support. The nutrition education given to the NE and NEP intervention groups included weekly (120 min each) nutrition classes conducted over 8 weeks by the principal investigator (PI).<br><br>The NE curriculum was written in English and supplemented by photos and illustrations to help the patient understand the content better. It focused on

	nutrition in relation to diabetes; food portion control for weight reduction; healthier food choices; individualized meal planning,; glycemic index and glycemic loads of different food and their importance in blood glucose control; the food pyramid, and its use together with food exchange list in meal planning. Patients learnt about the basics food groups, the difference between simple and complex carbohydrates and their relation to glycemic index and glycemic load, fibre content of different cereals and starches, the difference between saturated and unsaturated fats and their relation to diabetes management; sources of protein and the different nutrient content of each, hidden calories contained in beverages, and the micronutrient and fiber values of fruits and vegetables.<br>The nutrition education content was presented using lectures, demonstrations, discussions, and other participatory methods.<br>The physical activity lesson was given to the intervention groups (NE and NEP group) in the last week of the education programme. The aim of the physical activity was to ensure that patients accumulate a minimum of 150 min of moderate intensity exercise each week from personal activity at home that includes walking, digging, jogging, cycling, house hold duty, aerobics and sport activities. The participants were encouraged to perform the exercise at least three days each week with no more than two consecutive days without exercise. During the physical activity lesson, the patients were led through the importance of physical activity in management of T2D. Additionally, demonstrations on activities they could do at home were done by a physiotherapist experienced in diabetes management. The participants were encouraged to continue with the exercises at home in addition to normal routine work.<br><br>Participants in the NEP group were divided into small support groups (5–10 participants); depending on the location they came from as well as their age. After each education session, members of the support groups were encouraged to set and share with one another other weekly goals for specific changes in their eating and physical activity behavior. The goals were aimed at making healthy food choices, reduction of portion sizes and being active. The participants reported on their progress to the group members at the beginning of the next session. After the 8-week training, participants were followed monthly, and they presented their progress and new goals to the group members, for a period of 6 months. A trained peer educator living with diabetes for 13 years from Kenya Defeat Diabetes Association (KDDA) joined the PI during the monthly meetings and encouraged the participants in the peer support groups by sharing his experiences. Together with the PI he also assisted them to review and adjust their goals during monthly meetings. Also, group counseling was done on each visit for participants requiring more support.
Description/definition of peer support	The Nutrition education with Peer-to-Peer support (NEP) group were given peer-to-peer support training in addition to the nutrition training program. Members of the peer support group were encouraged to set and share with one another other weekly goals for specific changes in their eating and physical activity behaviour. A trained peer educator living with diabetes for

	13 years joined the PI during monthly meetings and encouraged participants in the peer support groups by sharing his experience.<br><br>Definition: Peer to peer social and emotional support has been shown to help people apply disease management or prevention plans in daily life, and links individuals with clinical, community, and other resources.
Mode of peer support	Face to face.
Setting/Context: Location or Primary site of delivery of intervention	Thika Level 5 Hospital.
Description of Control	Standard education that covered content on diabetes pathophysiology, risk factors, symptoms, complications, hyperglycemia and hypoglycemia symptoms, foot care treatment goals and modalities, blood glucose and blood pressure monitoring, treatment for those with problem as well as education on diabetes care by a clinician on monthly basis. The Standard education relied on pictorial flip charts and additional learning material with diabetes management information.
Duration of intervention and control	Intervention: 8 weeks
Time points of outcome measures	Baseline, 6 months post intervention.
List of outcomes and measurement tool	Weight: calibrated electronic weigh scale (Seca)<br>Height: stadiometer<br>BMI: WHO classification<br>Waist and hip circumference: anthropometric tape<br>Blood pressure: sphygmomanometer (model: Autortensio® noSPG440)<br>Serum triglycerides (TG), total cholesterol (TC), high density lipoprotein (HDL-c), low-density lipoprotein cholesterol (LDL-c): enzymatic method<br>Glycated hymoglobin (HbA1c): high-performance liquid chromatography<br>Blood glucose: glucose oxidase method<br>Physical activity: questionnaire<br>Dietary intake: adapted from perceived dietary adherence questionnaire (PDAQ), dietary approach to stop hypertension questionnaire (DASH), and medical nutrition therapy (MNT)
Outcomes mapped to Evans et al	(2) assistance in daily management,

Author, Year	Yin, 2015
Location (city, country)	Hong Kong
Objective	Examined the effects of participating in a "train-the-trainer" program and being a peer supporter on metabolic and cognitive/psychological/ behavioral parameters in Chinese patients with type 2 diabetes.
Study Design	Non randomized control trial.
Inclusion/Exclusion Criteria	Inclusion: Patients with type 2 diabetes aged 18 to 75 years with fair glycemic control (HbA1c <8%); good understanding of living with diabetes; clear communication skills; and a desire to serve<br><br>Exclusion criteria: illiteracy; physical impairment; and mental illness impairing communication with others
Overall n (number invited)	139
Intervention n (number invited)	79
Control n (number invited)	60
Loss to follow-up, n (%)	O: 20 (14); I: 20 (25); C: 0
Age Mean (SD)	I: 55.6 (11.5); C: 56.5 (10.9)
Female I: n (%); C: n (%)	I: 51 (65); C: 24 (40)
Male I: n (%); C: n (%)	I: 28 (35); C: 36 (65)
Diseases at Baseline	Type 2 Diabetes
BMI Mean (SD)	NR
Blood pressure Mean (SD)	I: SBP: 127 (13.6); DBP: 71 (7.0); C: SBP: 129 (29.5); DBP: 79 (11.2)
HbA1c Mean (SD)	I: 7.1 (0.3); C: 7.1 (0.5)
Description of Intervention	The program consisted of four monthly workshops, each lasting eight hours, for a total of 32 hours. Health care experts led the workshops, which included both didactic components and interactive components such as role playing and group sharing.<br>Participants who completed the training program and passed assessments were invited to be peer supporters. Those who agreed ("agreed trainees") were compared with those who declined ("refused trainees"). A group of patients from the same sites under usual care who had similar glycemic control but did not attend the training program were selected as comparison group subjects.<br>Each agreed trainee was assigned 10 patients of the same gender to support. The peer supporters were asked to provide structured peer support for at least one year, with provisions for a voluntary extension of three more years. Peer supporters were asked to give each of their assigned patients a 15- to 20-minute telephone call biweekly for the first three months, monthly for the second three months, and every two months for the last six months. Peer supporters were given a checklist to use in reviewing specific self-

	management skills, including medication adherence, healthy diet, regular exercise, sick day management, foot care, and glucose monitoring. They were also encouraged to provide psychological support based on their own experiences. Peer supporters submitted their phone call checklists every three months for documentation of their discussion items, duration of each call, and relevant remarks. Additional electronic communication and group gatherings were left to the discretion of the participants. During the voluntary extension period, the peer supporters were asked to maintain contact with their assigned patients every one to two months for another three years. They were also required to document the calls and return the checklists to the project coordinator every year.
Description/definition of peer support	Peers were those living with T2DM, aged 18 to 75 years with HbA1C <8%, had a good understanding of living with diabetes, clear communication skills. They underwent the "train-the-trainer" program.<br><br>Definition: Peer support refers to the transfer of experiential knowledge of a specific behavior or coping strategy for a stressor between people who share a particular characteristic.
Mode of peer support	Phone calls
Setting/Context: Location or Primary site of delivery of intervention	Managed in the usual care setting of their hospital or community-based clinic.
Description of Control	Usual care
Duration of intervention and control	Six months
Time points of outcome measures	Baseline, six months and yearly follow up for 4 years.
List of outcomes and measurement tool	HbA1c<br><br>blood pressure<br><br>lipid profile<br><br>cognitive/psychological behavioural measures: Depression Anxiety and Stress Scale (DASS), EuroQol-5D (EQ5D), Diabetes Empowerment Scale (DES), Patient Health Questionnaire (PHQ), General Health Questionnaire (GHQ), Summary of Diabetes Self Care Activities (SDSCA)
Outcomes mapped to Evans et al	(2) assistance in daily management,<br>(3) social and emotional support to promote disease self-management and coping with negative emotions,

Author, Year	Zeng, 2016
Location (city, country)	Shanghai, China
Objective	Evaluated the effectiveness of a community-based psychological intervention administered by non-specialized clinicians and volunteers for alleviating depressive and anxiety symptoms in individuals with chronic physical illnesses.
Study Design	Non-randomized controlled trial.
Inclusion/Exclusion Criteria	Inclusion: age≥ 18 years; resided in the community; registered at the community health center with a diagnosis of adult-onset diabetes or primary hypertension; no physical illness that was so severe it made it impossible to participate; no mental disorder or cognitive impairment that made it impossible to participate; written or oral informed consent<br><br>Exclusion: NR
Overall n (number invited)	10164
Intervention n (number invited)	8122
Control n (number invited)	2042
Loss to follow-up, n (%)	O: 5886 (58); I: 5083 (63); C: 803 (39)
Age Mean (SD)	I: 69.4 (10.3); C: 70.4 (10.3)
Female I: n (%); C: n (%)	I: 3776 (54.7); C: 1050 (54.8)
Male I: n (%); C: n (%)	I: 3121 (45.3); C: 866 (45.2)
Diseases at Baseline	Diabetes<br>Hypertension
BMI Mean (SD)	NR
Blood pressure Mean (SD)	NR
HbA1c Mean (SD)	NR
Description of Intervention	All participants received routine management of their chronic illness.<br>The community-based comprehensive psychological intervention used in this study was an adaptation of the IMPACT model developed in the United States for use in Shanghai. In addition to the routine management of their diabetes and/or hypertension, all intervention group subjects also received community based education about psychological health. Some individuals in the intervention group also received additional psychological support: individual counseling was offered to individuals whose baseline scores on the Patient Health Questionnaire-9 (PHQ-9) or the Generalized Anxiety Disorder 7-item scale (GAD-7) were >10; and small-group peer support was offered to individuals whose total score on either scale was >5.<br>The community-based mental health education component involved distributing brochures, broadcasting educational videos, and hosting lectures about psychosomatic health for individuals with chronic illnesses. The

	content focused on the identification and management of the symptoms of depression and anxiety, the relationship between psychological health and somatic health, and the relationship between stress and depression or anxiety.<br>The peer support group intervention targeted patients with diabetes or hypertension who had PHQ-9 or GAD-7 scores > 5 but also welcomed the participation of other community members who expressed interest in the groups. This intervention involved monthly 60-90 minute meetings led by community volunteers who had received guidance from counselors. The group meetings, which typically included 9-18 individuals, focused on: the management of chronic diseases; healthy lifestyles; psychological coping skills for dealing with diabetes and hypertension; knowledge about depression and anxiety; and self-awareness of negative emotions. In addition to the transmission of crucial information, the meetings also provided emotional and social support to the participants.<br>The individual intervention targeted individuals whose PHQ-9 or GAD-7 score was >10. Counselors provided one 60-minute and six 30-minute sessions of Problem Solving Treatment for Primary Care (PST-PC) to each individual. The counseling focused on alleviating symptoms of depression and anxiety by assisting these individuals to become more self-aware, to learn how to analyze and deal with their problems, to decrease their feelings of frustration, and to increase their feelings of control over their lives. PST has been found to be effective in the management of emotional problems among patients treated at community health centers.
Description/definition of peer support	Peers were community volunteers who had received guidance from counselors. Meetings by the peers focused on (a) the management of chronic diseases, (b) healthy lifestyles, (c) psychological coping skills for dealing with diabetes and hypertension, (d) knowledge about depression and anxiety, and (e) self-awareness of negative emotions. The meetings also provided emotional and social support to the participants.<br>Definition: N/R
Mode of peer support	Face to face.
Setting/Context: Location or Primary site of delivery of intervention	Community
Description of Control	All participants received routine management of their chronic illness which included registration, complete annual physical examinations, and quarterly follow-up of community residents with adult-onset diabetes and primary hypertension.<br>The quarterly follow-up assessments include assessment of blood pressure and fasting blood glucose, identification of sequelae or comorbid health conditions, health education about lifestyle issues, medication management, and, if necessary, referral to hospital outpatient or inpatient services for more extensive evaluation or treatment.
Duration of intervention and	6 months

control	
Time points of outcome measures	Baseline, 6 months post intervention.
List of outcomes and measurement tool	Depression: PHQ-9<br>Anxiety: GAD-7<br>Quality of life: SF-12
Outcomes mapped to Evans et al	(3) social and emotional support to promote disease self-management and coping with negative emotions,

Author, Year	Zhong, 2015
Location (city, country)	Anhui Province, China
Objective	Evaluated a peer leader–support program (PLSP) for diabetes self-management in China in terms of acceptability and feasibility; implementation; perceived advantages; disadvantages and barriers; reach and recruitment; effectiveness in terms of diabetes knowledge and clinical impacts; adoption; and sustainability.
Study Design	Randomized controlled trial.
Inclusion/Exclusion Criteria	Inclusion: diagnosis of type 2 diabetes made at a hospital, based on WHO criteria; age at least 15 years; residence in one of the project communities for more than one year.<br><br>Exclusion: inability to participate due to physical or mental disabilities.
Overall n (number invited)	726
Intervention n (number invited)	365
Control n (number invited)	361
Loss to follow-up, n (%)	O: 264 (36); I: 170 (47); C: 94 (26)
Age Mean (SD)	NR
Female I: n (%); C: n (%)	O: 356 (49)
Male I: n (%); C: n (%)	O: 370 (51)
Diseases at Baseline	Diabetes
BMI Mean (SD)	I: 24.3 (2.88); C: 23.5 (2.50)
Blood pressure Mean (SD)	I: SBP: 136 (14.91), DBP: 82.5 (8.72); C: SBP: 130 (11.80), DBP: 79.0 (8.67)
HbA1c Mean (SD)	NR
Description of Intervention	Nineteen “peer support groups,” one for each peer leader, were set up in the subcommunities randomized to the PLSP condition. Each group consisted of 10-15 participants. Twelve biweekly education meetings over six months to co-led by peer leaders with Community Health Service Center (CHSC) staff involvement titrated to peer leaders’ needs. Meetings lasted 1.5-2 hours and covered a range of topics such as diet, physical activity, medications, foot care, stress management and depression, barriers to self-management, and obtaining resources and support from the community, family, friends, and the health system. For efficiency, groups were often combined, resulting in meetings of more than 30 participants and consequently limited opportunity for discussion. Accordingly, peer leaders led 12 biweekly discussion meetings over six months. These reviewed the topics of the education meetings and included sharing experiences and modeling self-management practices.<br>Peer leaders also led or encouraged informal activities (for instance, walking and tai chi groups) among group members. Because peer leaders and participants lived within the same subcommunities, casual interactions and

	activities were common.
Description/definition of peer support	Peer leaders were those diagnosed with T2DM for more than 1 year, willing to volunteer and generally adhered to both medication and behavioral management regimens. Additional criteria were altruism, positive and sociable personality, availability of time, an understanding of the importance of patient confidentiality, good relationships with community residents and leadership in their communities. They underwent 3 days' training. Training emphasized the key functions of peer support promoted by Peers for Progress. Peer leaders were retired adults who had diabetes for a mean of 9.3 years.<br><br>Definition: Assistance in daily management, social and emotional support, linkage to clinical care and community resources, and ongoing availability of support.
Mode of peer support	face to face
Setting/Context: Location or Primary site of delivery of intervention	Community Health Service Centers and participants homes.
Description of Control	Usual care.
Duration of intervention and control	Six months
Time points of outcome measures	Baseline, six months post intervention.
List of outcomes and measurement tool	Knowledge<br>Attitudes<br>Self-Efficacy<br>Social support<br>Self-management (Diet, Physical activity, Glucose monitoring, Medication adherence)<br>BMI (kg/m^2)<br>SBP<br>DBP<br>Fasting glucose<br>2-Hour post-prandial glucose
Outcomes mapped to Evans et al	(2) assistance in daily management,<br>(3) social and emotional support to promote disease self-management and coping with negative emotions,
