# Supplementary material for: Peer Support for Type 2 Diabetes Management in Low- and Middle-Income Countries (LMICs): A Scoping Review
Source: Glob Heart. 2024 Feb 20;19(1):20. doi: 10.5334/gh.1299 (PMC10885823; doi:10.5334/gh.1299)
Supplement: Supplemental File 3. — TIDiER. [file gh-19-1-1299-s3.pdf]

Supplemental File 3: TIDiER

	TIDiER Tool Item											
	Name	Why	What		Who	How	Where	When and How much	Tailoring	Modification	How well	
Author, year			Materials	Procedures							Planned	Actual
Ahmadi, 2018	X	X	N/A	X	X	X	N/R	X	N/R	N/R	N/R	N/R
Assah, 2015	X	X	N/R	X	X	X	X	X	X	N/R	N/R	N/R
Baumann, 2015	X	X	X	X	X	X	N/R	X	N/R	N/R	X	X
Castillo-Hernandez, 2021	X	X	X	X	X	X	X	X	X	N/R	N/R	N/R
Chan, 2014	X	X	X	X	X	X	X	X	X	N/R	N/R	N/R
Debussche, 2018	X	X	X	X	X	X	N/R	X	N/R	X	N/R	N/R
Gagliardino, 2013	X	X	X	X	X	X	X	X	N/R	N/R	N/R	N/R
Ghasemi, 2019	X	X	N/A	X	X	X	X	X	N/R	N/R	N/R	N/R
Hernandez, 2021	X	X	N/R	X	X	X	N/R	N/R	X	N/R	N/R	N/R
Ju, 2018	X	X	X	X	X	X	X	X	X	N/R	N/R	N/R
Khan, 2018	X	X	X	X	X	X	N/R	X	X	N/R	N/R	N/R
Khetan, 2019	X	X	X	X	X	X	X	X	X	N/R	X	N/R
Latina, 2020	X	X	X	X	X	X	X	X	X	N/R	X	N/R
Liu, 2020	X	X	X	X	X	X	X	X	X	X	N/R	N/R
Mwakalinga 2021	X	X	N/A	X	N/A	N/R	X	N/A	N/A	N/R	N/R	N/R
Paz-Pacheco, 2017	X	X	N/R	X	X	X	X	X	N/R	N/R	N/R	N/R
Peimani, 2018	X	X	X	X	X	X	X	X	X	N/R	N/R	N/R
Rao, 2020	X	X	N/R	X	X	X	N/R	X	N/R	N/R	N/R	N/R
Rotheram-Borus, 2012	X	X	X	X	X	X	X	X	X	N/R	N/R	N/R
Sazlina, 2015	X	X	N/R	X	X	X	N/R	X	X	N/R	N/R	N/R
Shahsavari, 2021	X	X	X	X	X	X	X	X	N/R	X	N/R	N/R
Sreedevi, 2017	X	X	X	X	X	X	N/R	X	N/R	N/R	N/R	N/R
Taniguchi, 2018	X	X	N/R	X	X	X	X	X	X	N/R	N/R	N/R
Thuita, 2020	X	X	X	X	X	X	X	X	X	N/R	N/R	N/R
Yin, 2015	X	X	X	X	X	X	N/A	X	X	N/R	N/R	N/R
Zeng, 2016	X	X	X	X	X	X	X	X	X	N/R	X	X
Zhong, 2015	X	X	X	X	X	X	X	X	N/R	X	X	X

Ahmadi, 2018	
**TIDiER Tool Item**	**Main Paper**
**BRIEF NAME**<br>Provide the name or a phrase that describes the intervention.	Outcomes of peer-led diabetes education compared to education delivered by health professionals
**WHY**<br>Describe any rationale, theory, or goal of the elements essential to the intervention.	This study aimed to compare the effect of education by health care provider and peer on self-care behaviours among Iranian patients with diabetes.
**WHAT**<br>Materials: Describe any physical or informational materials used in the intervention, including those provided to participants or used in intervention delivery or in training of intervention providers. Provide information on where the materials can be accessed (e.g., online appendix, URL).	N/A
Procedures: Describe each of the procedures, activities, and/or processes used in the intervention, including any enabling or support activities.	Sessions were held in groups of 20 patients. Session content was designed based on the needs of a diabetes patient with particular attention to the main items of the SDSCA questionnaire such as diet control, physical activities, medication adherence and foot care which was all coordinated with the peer. The peer was asked to exchange his experience in diabetes control.<br>Simultaneously, the control group received the clinic's routine diabetes education program and did not undergo any intervention. After 12 weeks, we evaluated the self-care behaviors of all patients in the three groups.
**WHO PROVIDED**<br>For each category of intervention provider (e.g., psychologist, nursing assistant), describe their expertise, background and any specific training given.	Inclusion criteria for selection of peer consisted of good control of blood glucose, a few complications, ability to manage sessions, personal interest to collaborate and provide support, good social communication skills (e.g. good appearance, tone of voice, eye contact) and education higher than middle school.<br>The chosen peer received training on managing sessions and implementing the educational program.
**HOW**<br>Describe the modes of delivery (e.g., face-to-face or by some other mechanism, such as internet or telephone) of the intervention and whether it was provided individually or in a group.	Face to face, group sessions
**WHERE**<br>Describe the type(s) of location(s) where the intervention occurred, including any necessary infrastructure or relevant features.	N/R
**WHEN and HOW MUCH**<br>Describe the number of times the intervention was delivered and over what period of time including the number of sessions, their schedule, and their duration, intensity or dose.	12 weeks of education, one session per week during the first six weeks and one session every other week during the second 6 weeks. Each session lasted for 1 h.<br>12 week intervention
**TAILORING**<br>If an intervention was planned to be personalised, titrated or adapted, then describe what, why, when, and how.	N/R
**MODIFICATION**	N/R

If an intervention was modified during the course of the study, describe the changes (what, why, when, and how).	
**HOW WELL**<br>Planned: If intervention adherence or fidelity was assessed, describe how and by whom, and if any strategies were used to maintain or improve fidelity, describe them.	N/R
Actual: If intervention adherence or fidelity was assessed, describe the extent to which the intervention was delivered as planned.	N/R

Assah, 2015	
**TIDiER Tool Item**	**Main Paper**
**BRIEF NAME**<br>Provide the name or a phrase that describes the intervention.	Community-based peer support in people with Type 2 diabetes
**WHY**<br>Describe any rationale, theory, or goal of the elements essential to the intervention.	To examine the effectiveness of a community-based multilevel peer support intervention in addition to usual diabetes care on improving glycaemic levels, blood pressure and lipids in patients with Type 2 diabetes in Yaounde, Cameroon.
**WHAT**<br>Materials: Describe any physical or informational materials used in the intervention, including those provided to participants or used in intervention delivery or in training of intervention providers. Provide information on where the materials can be accessed (e.g., online appendix, URL).	N/R
Procedures: Describe each of the procedures, activities, and/or processes used in the intervention, including any enabling or support activities.	The intervention arm underwent a structured community based multilevel peer support intervention adapted to the sociocultural context of Cameroon, in addition to their usual clinical care. The control arm continued usual clinical care.<br>A peer supporter was recruited for each of the 10 groups of the intervention arm based on their past history and clinical profile. These peer supporters, who were invited to volunteer, underwent a 2–day training workshop that fortified their knowledge and skills to support persons with diabetes, emphasizing on building and reinforcing the participant's knowledge on diabetes, training on communication skills, effective group and face-to-face meetings, and use of personal history as examples in peer support.<br>The peer support intervention was implemented through group meetings, personal encounters between peer supporters and their group members, and telephone calls. There were six group meetings following a standard schedule, five monthly personal encounters and phone calls between peers and group members. Group meetings (on diet and healthy eating, physical exercise, observance to treatment, feet and body care, complications of diabetes and living with diabetes), were held monthly.
**WHO PROVIDED**<br>For each category of intervention provider (e.g., psychologist, nursing assistant), describe their expertise, background and any specific training given.	Trained peer supporters with better glycaemic and metabolic control than their peers, more compliant with their clinic visits, and more experiential knowledge on diabetes
**HOW**<br>Describe the modes of delivery (e.g., face-to-face or by some other mechanism, such as internet or telephone) of the intervention and whether it was provided individually or in a group	Face to face, group and individual<br>Telephone, individual
**WHERE**<br>Describe the type(s) of location(s) where the	Group meetings (on diet and healthy eating, physical exercise, observance to treatment, feet and body care,

intervention occurred, including any necessary infrastructure or relevant features.	complications of diabetes and living with diabetes), were held monthly at locations related to each group's common affinity, out of the hospital setting.
**WHEN and HOW MUCH**<br>Describe the number of times the intervention was delivered and over what period of time including the number of sessions, their schedule, and their duration, intensity or dose.	6 scheduled group meetings<br>5 monthly personal encounters and phone calls<br>Length of visits N/R<br><br>6 month intervention
**TAILORING**<br>If an intervention was planned to be personalised, titrated or adapted, then describe what, why, when, and how.	Planned to be personalised
**MODIFICATION**<br>If an intervention was modified during the course of the study, describe the changes (what, why, when, and how).	N/R
**HOW WELL**<br>Planned: If intervention adherence or fidelity was assessed, describe how and by whom, and if any strategies were used to maintain or improve fidelity, describe them.	N/R
Actual: If intervention adherence or fidelity was assessed, describe the extent to which the intervention was delivered as planned.	N/R

Baumann, 2015	
**TIDiER Tool Item**	**Main Paper**
**BRIEF NAME**<br>Provide the name or a phrase that describes the intervention.	Peer Support for Adults with Type 2 Diabetes
**WHY**<br>Describe any rationale, theory, or goal of the elements essential to the intervention.	The purpose of this pre-post quasi-experimental study was to test the feasibility of a peer intervention to improve the following: diabetes self-care behaviors, glycemic control, social support and emotional well-being, linkages to health care providers, and to assess the sustainability of the intervention 18 months later.
**WHAT**<br>Materials: Describe any physical or informational materials used in the intervention, including those provided to participants or used in intervention delivery or in training of intervention providers. Provide information on where the materials can be accessed (e.g., online appendix, URL).	Packet of materials for participants that contained the following: a consent form, the Diabetes Self-Care Questionnaire, Screening Data Form, Take Care of Your Feet poster, Peer Champion Contact Logbook, Peer Champion Training Booklet, and "The ABC's of Diabetes" brochure
Procedures: Describe each of the procedures, activities, and/or processes used in the intervention, including any enabling or support activities.	The intervention was designed to address key functions of peer support: (1) assistance in applying disease management or prevention in daily life, (2) emotional and social support, (3) linkage to clinical care, and (4) ongoing support.<br>Ugandan physicians and nurses, who are specialists in diabetes care, delivered the diabetes training sessions in English for champions and in both English and Luganda for the partners.<br>This single-group pre-post study examined a 4-month peer support intervention in which participants were trained in diabetes self-care, some serving as peer champions and others as peer partners. Participants were asked to complete a written contact log after each contact with a peer; a prepaid telephone network was activated among all participants, and call logs were recorded electronically. Measures of diabetes self-care and physiologic outcomes were obtained at a final group meeting 4 months later (T2).<br>At a 1-day meeting (T1), held separately 2 weeks apart for champions and partners, all participants completed premeasures and received 5 hours of education on diabetes self-care. Additionally, the champions received 1 hour of review and role play in using supportive communication skills, such as active listening and providing assistance with daily management. At the conclusion of the partner meeting, the champions and partners were matched in pairs or triads by age and gender and agreed to make telephone or personal contact weekly throughout the trial period. All participants were provided with mobile phones linked to a prepaid network so that calls could be made at no cost.<br>The curriculum addressed areas of diabetes self-care that included healthy eating, being active, taking medications, monitoring blood sugar, problem solving, reducing risks, and problem solving. All participants were given a packet

	of materials that contained the following: a consent form, the Diabetes Self-Care Questionnaire, Screening Data Form, Take Care of Your Feet poster, Peer Champion Contact Logbook, Peer Champion Training Booklet, and "The ABC's of Diabetes" brochure. Materials were written at the fifth grade reading level and printed in a large font.
**WHO PROVIDED**<br>For each category of intervention provider (e.g., psychologist, nursing assistant), describe their expertise, background and any specific training given.	Champions who had to read and speak English and receive additional training in communication skills to provide peer partners emotional support and assistance with daily management.<br>Ugandan physicians and nurses, who are specialists in diabetes care.
**HOW**<br>Describe the modes of delivery (e.g., face-to-face or by some other mechanism, such as internet or telephone) of the intervention and whether it was provided individually or in a group.	Face to face, individual or group<br>Telephone, individual
**WHERE**<br>Describe the type(s) of location(s) where the intervention occurred, including any necessary infrastructure or relevant features.	N/R
**WHEN and HOW MUCH**<br>Describe the number of times the intervention was delivered and over what period of time including the number of sessions, their schedule, and their duration, intensity or dose.	1 day meeting for champions and partners held separately 2 weeks apart. All participants received 5 hours of education with an additional 1 hour of education for champions.<br>4 month intervention
**TAILORING**<br>If an intervention was planned to be personalised, titrated or adapted, then describe what, why, when, and how.	N/R
**MODIFICATION**<br>If an intervention was modified during the course of the study, describe the changes (what, why, when, and how).	N/R
**HOW WELL**<br>Planned: If intervention adherence or fidelity was assessed, describe how and by whom, and if any strategies were used to maintain or improve fidelity, describe them.	Participant Logbooks All participants were given a paper logbook in which they were to record each peer contact. The champion logbook included four items: date of contact, topic discussed, result of the discussion, and plan for next contact. The partner logbook included the following: date of contact, goal for the week, change(s) made, and moods and feelings.<br><br>Phone Records A prepaid monthly closed network user group was purchased for mobile phones to allow participants to call any of the participants, the Mityana Diabetes Clinic nurse and study partners from Mulago Hospital. Phone activity was electronically tracked over the intervention period and included the origination number, recipient number, date, time, and duration of the call.<br><br>Narrative Notes The study nurse recorded every contact between study participants and research staff, including the date, participant identification number, and a brief

	description of the nature of the call or visit and advice given. Narrative summaries were taken by the study nurse of the educational meetings held at the diabetes clinic at 2 and 3 months during the intervention.
Actual: If intervention adherence or fidelity was assessed, describe the extent to which the intervention was delivered as planned.	Electronic phone logs and data from participant logbooks show that most participants both utilized the telephones and network of peer supporters and contacted the diabetes clinic more often than preintervention. Electronic phone records showed that 68 % of participants made a phone contact with a peer at least weekly. Attendance for the first and second educational meetings was 76 (n=31) and 88 % (n=36), respectively. When reporting total contact with peers, 93 % (n=40) used cell phones, and 60 % (n=28) reported personal contact. Of participants who completed the study, no one had fewer than six contacts with a peer during the intervention period. An item from the post-questionnaire about how often they contacted the diabetes clinic, using a three-point scale (more often, less often, or same as before the program), showed that 89.7 % (n=35) of the participants reported increased contact with a health care provider during the intervention.

Castillo-Hernandez, 2021	
**TIDiER Tool Item**	**Main Paper**
**BRIEF NAME**<br>Provide the name or a phrase that describes the intervention.	Peer Support Added to Diabetes Education in Adults Living with Type 2 Diabetes
**WHY**<br>Describe any rationale, theory, or goal of the elements essential to the intervention.	The objective of our study was to evaluate the effect of peer support added to a diabetes education program on glycemic control and diabetes related quality of life when compared with a conventional diabetes education program in patients with type 2 diabetes in a Mayan community in Mexico.
**WHAT**<br>Materials: Describe any physical or informational materials used in the intervention, including those provided to participants or used in intervention delivery or in training of intervention providers. Provide information on where the materials can be accessed (e.g., online appendix, URL).	Leaders were trained to start each meeting with an "icebreaker" introduction, followed by a discussion session and goal setting, as described in the Peer Leader Manual (manual was referenced)
Procedures: Describe each of the procedures, activities, and/or processes used in the intervention, including any enabling or support activities.	All participants in the study and PLs attended a 4-month government-sponsored DSME program named "A 7 Pasos del Control" (i.e. "Seven Steps to Achieve Control") delivered by a dietitian and diabetes educator certified by the Mexican Council of Diabetes Educators. PLs had the additional role of providing logistics support to study participants during the general educational sessions, if needed. The DSME program consisted of 16 1-hour weekly group sessions that were offered both in the morning and afternoon to increase opportunities for participation. In addition, all study participants completed a comprehensive one on-one nutrition counselling session with a dietitian during the first month of the study. Study subjects were also encouraged to participate in 2 50-minute exercise sessions per week during the 8 months of the study. These sessions were led by certified trainers who had experience in working with adults with chronic conditions.<br>Participants in the PSEG also attended peer-support meetings facilitated by 9 PLs throughout the study. Each PL facilitated a single group of 3 or 4 participants. Each meeting was facilitated by the PL without the presence of health professionals. The discussion segment was based on the DSME theme of the previous education session for the first 4 months, and for the remaining sessions the theme was identified by each PL according to the needs and interests of the groups.
**WHO PROVIDED**<br>For each category of intervention provider (e.g., psychologist, nursing assistant), describe their expertise, background and any specific training given.	DSME delivered by a dietitian and diabetes educator certified by the Mexican Council of Diabetes Educators.<br><br>Exercise sessions were led by certified trainers who had experience in working with adults with chronic conditions.
**HOW**<br>Describe the modes of delivery (e.g., face-to-face or by some other mechanism, such as internet or telephone) of the intervention and whether it was	Face to face, group

provided individually or in a group.	
**WHERE**<br>Describe the type(s) of location(s) where the intervention occurred, including any necessary infrastructure or relevant features.	The trial was conducted at a community centre in the town of Komchén, a semirural Mayan village located approximately 25 kilometers (16 miles) from Mérida, the closest urban area, in the State of Yucatán, Mexico.<br>Peer-support meetings were held in the community premises or at the homes of the PLs at a convenient time for both the leaders and participants.
**WHEN and HOW MUCH**<br>Describe the number of times the intervention was delivered and over what period of time including the number of sessions, their schedule, and their duration, intensity or dose.	The DSME program consisted of 16 1-hour weekly group sessions PL support intervention took place during the first 16 weeks and 4 months after.<br>There were 20 peer-support meetings over 8 months per group: 1 session every week, except for 2 holiday weeks and during a chikungunya outbreak<br>The duration of the meetings varied between 30 and 60 minutes.<br>8 month intervention
**TAILORING**<br>If an intervention was planned to be personalised, titrated or adapted, then describe what, why, when, and how.	The discussion segment was based on the DSME theme of the previous education session for the first 4 months, and for the remaining sessions the theme was identified by each PL according to the needs and interests of the groups.
**MODIFICATION**<br>If an intervention was modified during the course of the study, describe the changes (what, why, when, and how).	N/R
**HOW WELL**<br>Planned: If intervention adherence or fidelity was assessed, describe how and by whom, and if any strategies were used to maintain or improve fidelity, describe them.	N/R
Actual: If intervention adherence or fidelity was assessed, describe the extent to which the intervention was delivered as planned.	N/R

Chan, 2014	
**TIDiER Tool Item**	**Main Paper**
**BRIEF NAME**<br>Provide the name or a phrase that describes the intervention.	Peer Support, Empowerment, and Remote Communication Linked by Information Technology [PEARL]
**WHY**<br>Describe any rationale, theory, or goal of the elements essential to the intervention.	To investigate if frequent contacts through a telephone-based peer support program (Peer Support, Empowerment, and Remote Communication Linked by Information Technology [PEARL]) would improve cardiometabolic risk and health outcomes by enhancing psychological well-being and self-care in patients receiving integrated care implemented through a web-based multicomponent quality improvement program (JADE [Joint Asia Diabetes Evaluation]).<br>*Related to Yin et al 2015
**WHAT**<br>Materials: Describe any physical or informational materials used in the intervention, including those provided to participants or used in intervention delivery or in training of intervention providers. Provide information on where the materials can be accessed (e.g., online appendix, URL).	JADE portal (refer to video at http://www.idfce-hk.org)<br>All peer supporters were given a booklet on resources (eg, websites and telephone numbers of community centers, lay associations, and hospital diabetes centers and titles of self-help books) and a 3-monthly checklist to document the discussion items (diet, exercise, self-monitoring of blood glucose, sick day management, foot care, emotional support, resources for information, and clinical care), duration of each call, and relevant remarks
Procedures: Describe each of the procedures, activities, and/or processes used in the intervention, including any enabling or support activities.	The JADE Program<br>The study was conducted in 3 diabetes centers that provide twice-weekly structured comprehensive assessments implemented through the JADE portal. Using these clinical and biochemical results, the JADE portal generated 1 of 4 risk categories based on different combinations of cardiovascular-renal complications, chronic kidney disease (CKD) (defined as estimated glomerular filtration rate [eGFR] less than 60 mL/min/1.73m2), risk scores for cardiovascular-renal disease, and number of risk factors (see eAppendix in Supplement). All patients received their reports 4 to 6 weeks later during a 2-hour nurse led group empowerment class with reinforcement on selfcare and attainment of multiple treatment targets. All patients were followed up in their usual clinics every 3 to 4 months, when most physicians ordered HbA1c measurement and recorded BP and bodyweight, in accordance with international guidelines. However, as in most public health care institutions, different physicians reviewed these patients with short consultation time. In this project, we enhanced the care by using a research assistant to retrieve the appointment dates, laboratory results, and clinic measurements from the Clinical Management System. The available data were entered into the JADE portal to generate follow-up reports, which were mailed to the patients with a cover letter, encouraging them to discuss their progress with their care team as appropriate.

	For patients assigned to the JADE + PEARL group, the nurses put them in groups of 10 and arranged a separate 2-hour session when 2 to 3 groups of patients were introduced to their assigned peer supporters, each of whom was assigned 10 patients. During these weekend sessions, the nurses facilitated group sharing on self-care and stress management. After exchanging telephone numbers, the peer supporters were instructed to call their assigned peers at least 12 times—initially, biweekly calls for 3 months, then monthly calls for 3 months, and then 1 call every other month for 6 months, with an anticipated 15 minutes per call. Both peer supporters and peers were encouraged to call one another ad lib.
**WHO PROVIDED**<br>For each category of intervention provider (e.g., psychologist, nursing assistant), describe their expertise, background and any specific training given.	The diabetes nurses first invited 79 motivated patients with HbA1c levels lower than 8% to attend a "Train-the-Trainer" program consisting of four 8-hour workshops, each attended by 30 to 35 patients. The training program was designed by health care professionals and behavioral scientists and run by neurolinguistic consultants, sports scientists, psychologists, nurses, and physicians. The training format included tutorials, case sharing, reflections, role playing, games, and activities with peer supporters receiving tutorial notes and reference materials. Throughout these sessions, they were reinforced on the principles of communication and empathic listening and encouraged to share their positive experiences to assist their peers to manage diabetes on a day-to-day basis. The peer supporters were reminded of factors that could influence blood glucose level, eg, diet, exercise, poor sleep, stress, changes in daily routines, body weight, medications, and concurrent illnesses, and thus the importance of self-monitoring of blood glucose. Some of them were active members of patient groups organized by lay associations or diabetes centers. All participants underwent a before and after evaluation of diabetes knowledge and psychological-behavioral measures.<br><br>33 agreed to become peer supporters and attended an additional 3-hour briefing session on the rationale, objectives, and protocol of the study, led by the project team. Of these 33 peer supporters, 35% were male and the mean (SD) age, disease duration, and HbA1c level were 55.6 (11.5) years, 11.03 (6.71) years, and 7.25% (1.27%), respectively; 9 were treated with insulin. Among them, 29 had secondary school or higher education, 7 of whom had tertiary education. The majority of them were retired managers or skilled or nonmanual workers.
**HOW**<br>Describe the modes of delivery (e.g., face-to-face or by some other mechanism, such as internet or telephone) of the intervention and whether it was provided individually or in a group.	Face to face and telephone, group and individual
**WHERE**	Diabetes centre and home (phone calls/virtual)

Describe the type(s) of location(s) where the intervention occurred, including any necessary infrastructure or relevant features.	
**WHEN and HOW MUCH**<br>Describe the number of times the intervention was delivered and over what period of time including the number of sessions, their schedule, and their duration, intensity or dose.	1 intro meeting of 2hrs<br>12 phone calls with peers over 12 months (2/month for months 1 to 3, 1 per month for months 4 to 6, every other month for months 7 to 12) 15 min phone calls<br>12 month intervention
**TAILORING**<br>If an intervention was planned to be personalised, titrated or adapted, then describe what, why, when, and how.	Planned to be personalised
**MODIFICATION**<br>If an intervention was modified during the course of the study, describe the changes (what, why, when, and how).	N/R
**HOW WELL**<br>Planned: If intervention adherence or fidelity was assessed, describe how and by whom, and if any strategies were used to maintain or improve fidelity, describe them.	N/R
Actual: If intervention adherence or fidelity was assessed, describe the extent to which the intervention was delivered as planned.	N/R

Debussche, 2018	
**TIDiER Tool Item**	**Main Paper**
**BRIEF NAME**<br>Provide the name or a phrase that describes the intervention.	The Structured Type 2 Diabetes Self-Management Education by Peers (ST2EP)
**WHY**<br>Describe any rationale, theory, or goal of the elements essential to the intervention.	Our objective was to evaluate the effectiveness of peer-led self-management education in improving glycaemic control in patients with type 2 diabetes in a low-income country (Mali).<br><br>The peer-led structured patient education intervention drew on the 'Learning Nests' (Nids d'apprentissage) approach, which has been described elsewhere. Briefly, this empowerment-based approach, derived from socio constructivist theory takes into account the context of the illness, prevailing health practices, and the chronic dimension of the disease. It promotes patients' understanding of key concepts in their interactions with their social environment.
**WHAT**<br>Materials: Describe any physical or informational materials used in the intervention, including those provided to participants or used in intervention delivery or in training of intervention providers. Provide information on where the materials can be accessed (e.g., online appendix, URL).	Educational materials and booklets for participants<br>Booklets for peer educators (Education Prévention des Maladies Chroniques or EPMC booklets)
Procedures: Describe each of the procedures, activities, and/or processes used in the intervention, including any enabling or support activities.	The ST2EP intervention included 3 courses delivered in the community by trained peer educators over 1 year. Each course was composed of 4 different thematic sessions (4–10 participants) offered over a period of 3 months. Duration of sessions during the trial with peer educators was 1.5–2 hours. The themes addressed were cardiovascular risk management, food intake, exercise, and blood glucose and insulin management. The content, approach and programme of each group session were detailed in specific booklets for learners (including learners with literacy difficulties) and culturally adapted for Mali (food habits, language specificities, occupational and environment issues). Peer educators were also given specific booklets that comprised the session programme in French (Education Prévention des Maladies Chroniques or EPMC booklets), allowing for the replication of the educational intervention.
**WHO PROVIDED**<br>For each category of intervention provider (e.g., psychologist, nursing assistant), describe their expertise, background and any specific training given.	Trained peer educators selected with the following criteria: having diabetes, living in the locality, undergoing regular checks with a referent physician, volunteering to deliver educational sessions, and being fluent in both French and the local Bambara<br>language.
**HOW**<br>Describe the modes of delivery (e.g., face-to-face or by some other mechanism, such as internet or telephone) of the intervention and whether it was	Face to face, group

provided individually or in a group.	
**WHERE**<br>Describe the type(s) of location(s) where the intervention occurred, including any necessary infrastructure or relevant features.	N/R
**WHEN and HOW MUCH**<br>Describe the number of times the intervention was delivered and over what period of time including the number of sessions, their schedule, and their duration, intensity or dose.	4 different thematic sessions (4–10 participants) offered over a period of 3 months (months 1–3, 7–9, and 10–12). Sessions lasting 1.5 - 2 hours long
**TAILORING**<br>If an intervention was planned to be personalised, titrated or adapted, then describe what, why, when, and how.	N/R
**MODIFICATION**<br>If an intervention was modified during the course of the study, describe the changes (what, why, when, and how).	The initial protocol stated 1.5 hour-long sessions, however, the actual duration of sessions during the trial with peer educators was 1.5–2 hours.
**HOW WELL**<br>Planned: If intervention adherence or fidelity was assessed, describe how and by whom, and if any strategies were used to maintain or improve fidelity, describe them.	N/R
Actual: If intervention adherence or fidelity was assessed, describe the extent to which the intervention was delivered as planned.	N/R

Gagliardino, 2013	
**TIDiER Tool Item**	**Main Paper**
**BRIEF NAME**<br>Provide the name or a phrase that describes the intervention.	Type 2 diabetes patients educated by other patients perform as well as patients trained by professionals
**WHY**<br>Describe any rationale, theory, or goal of the elements essential to the intervention.	Compared clinical, metabolic and psychological outcomes in people with type 2 diabetes 1 year after attending a structured diabetes education programme implemented by professional educators versus the same programme implemented by trained peers with diabetes that also provided ongoing peer support.
**WHAT**<br>Materials: Describe any physical or informational materials used in the intervention, including those provided to participants or used in intervention delivery or in training of intervention providers. Provide information on where the materials can be accessed (e.g., online appendix, URL).	All participants received illustrated educational materials were used, as well as a book provided to each patient that included the main contents of the<br>programme<br>To test the diabetes knowledge of the participants, a multiple-choice questionnaire was used.
Procedures: Describe each of the procedures, activities, and/or processes used in the intervention, including any enabling or support activities.	the study included two different groups. The first group is the control patient education group (control) that received the educational intervention at the Houssay Centre (details of its modality and contents have been previously reported [23]). The Diabetes Structured Education Courses for People with T2DM was released through trained educators to no more than ten ambulatory patients in a group setting that allowed active interaction between the educator and participants. It consisted of four weekly teaching units (90–120 min each) and a reinforcement session at 6 months.<br>The first teaching unit included general concepts about type 2 diabetes, the symptoms of hypoglycaemia and hyperglycaemia and glucose self-monitoring, with strong emphasis on the importance of active patient participation in disease control and treatment.<br>In the second teaching unit, the effect of obesity on insulin sensitivity and the advantages of weight reduction and of patient learning to classify and select foods according to their calorie content were discussed.<br>The third teaching unit explained the importance of foot care and regular practice of physical activity, while during the fourth unit they learned the basic rules for ‘sick days’ and which were the examinations and laboratory tests necessary to have good diabetes care. Many illustrated educational materials were used, as well as a book provided to each patient that included the main contents of the programme. To test the diabetes knowledge of the participants, we used a multiple-choice questionnaire.<br>The second group is the peer patient education plus peer support group (peer) that received identical education plus the active participation of peers, who were integrated into the educational models and specific peer activities. The goal of the latter activities was to provide continuing psychological and behavioural support and to<br>teach the patients how to apply in everyday life the

	knowledge acquired during the education course, based upon the peer's personal experience. The peer's postcourse role and activity were complementary to the formal education; for that purpose, we implemented two different activities: (a) peer education and (b) peer support. Peer education was integrated into the educational units serving as 'real world living models' for the attendee. One peer worked fulltime and was responsible for the overall management of peers; he or she received a small direct compensation for his or her teaching, supervisory and administrative role.<br>For each educational module of the course, there was a specific set of supporting activities that the educator-peer shared with the supporting peers. To test the diabetes knowledge of participants, we used the same multiple-choice questionnaire mentioned earlier. Following the initial education course, peers had regular and continuing scheduled contacts with their supportees. Their contacts took the combined pre-established form of scheduled face-to-face visits or whenever a specific issue warranted, and frequent interactions by mobile telephone. The face-to-face visits among peers and their supportees were scheduled every second month.<br>The telephone communications took place at least weekly for the first 6 months, biweekly for the next 3 months and monthly for the remaining study period.<br>They were based on structured interviews that inquired into the patients' clinical, metabolic and psychological progress. This information was recorded and sent to the coordinator, becoming part of the patient's follow-up. In addition to these one-on-one telephone calls, we promoted monthly group calls among peers to share experiences, difficulties and alternative solutions implemented. A critical role of the peers was to provide throughout this system the psychological support that their supportees needed to cope with the day-to-day vicissitudes of diabetes self-care. Thus, more frequent interactions in person or by telephone were encouraged at times when more intensive psychological support was considered appropriate.
**WHO PROVIDED**<br>For each category of intervention provider (e.g., psychologist, nursing assistant), describe their expertise, background and any specific training given.	Trained Peers were recruited on the basis of their excellent diabetes control, self-motivation, communication and support skills and interest<br><br>Trained educators
**HOW**<br>Describe the modes of delivery (e.g., face-to-face or by some other mechanism, such as internet or telephone) of the intervention and whether it was provided individually or in a group.	face to face, group<br>telephone, individual
**WHERE**<br>Describe the type(s) of location(s) where the intervention occurred, including any necessary	The Bernardo A. Houssay Centre in the city of La Plata is a non-profit entity supported by funds from governmental organizations such as the Health Ministry of the province

infrastructure or relevant features.	of Buenos Aires, the pharmaceutical industry and private organizations such as Rotary International, the International Diabetes Federation and personal donors.<br>The Houssay Centre is a referral centre for the education of both people with diabetes and health professionals.
**WHEN and HOW MUCH**<br>Describe the number of times the intervention was delivered and over what period of time including the number of sessions, their schedule, and their duration, intensity or dose.	4 weekly session lasting 90-120 minutes each and a reinforcement session at 6 months.<br><br>Telephone communications took place at least weekly for the first 6 months, biweekly for the next 3 months and monthly for the remaining study period.<br><br>4 education course, one year study period
**TAILORING**<br>If an intervention was planned to be personalised, titrated or adapted, then describe what, why, when, and how.	N/R
**MODIFICATION**<br>If an intervention was modified during the course of the study, describe the changes (what, why, when, and how).	N/R
**HOW WELL**<br>Planned: If intervention adherence or fidelity was assessed, describe how and by whom, and if any strategies were used to maintain or improve fidelity, describe them.	N/R
Actual: If intervention adherence or fidelity was assessed, describe the extent to which the intervention was delivered as planned.	N/R

Ghasemi, 2019	
**TIDiER Tool Item**	**Main Paper**
**BRIEF NAME**<br>Provide the name or a phrase that describes the intervention.	Peer Group Education on the Quality of Life of Elderly Individuals with Diabetes
**WHY**<br>Describe any rationale, theory, or goal of the elements essential to the intervention.	Considering the important role of education and the benefits of peer education in increasing patients' independence in self-care, as well as the socio-economic benefit of using peer education within the public health system, the present study was conducted to assess the effect of peer education on QOL of elderly patients with diabetes.
**WHAT**<br>Materials: Describe any physical or informational materials used in the intervention, including those provided to participants or used in intervention delivery or in training of intervention providers. Provide information on where the materials can be accessed (e.g., online appendix, URL).	N/A
Procedures: Describe each of the procedures, activities, and/or processes used in the intervention, including any enabling or support activities.	The control group, consisting of 23 individuals, received training by the researcher. The intervention group, consisting of 21 individuals, who were interested and highly motivated was educated by peers. Both groups received eight sessions of training, each lasting 30–45 minutes. The content of the training sessions consisted of educational information regarding self-care, including exercise, diet, and skin care, and elements regarding QoL and common worries related to diabetes using lectures, discussions, and question and answer. The content of the sessions were devised under the supervision of the researcher and a diabetes specialist. The educational sessions in both groups were held at the health centers (Imam Ali and Ghaedi centers) on Tuesdays and Wednesdays at different times (at 9–10 a.m. for the peer-trained group and 10–11 a.m. for the researcher-trained group).
**WHO PROVIDED**<br>For each category of intervention provider (e.g., psychologist, nursing assistant), describe their expertise, background and any specific training given.	researcher for control group peers for intervention group background and training
**HOW**<br>Describe the modes of delivery (e.g., face-to-face or by some other mechanism, such as internet or telephone) of the intervention and whether it was provided individually or in a group.	Face-to-face, group
**WHERE**<br>Describe the type(s) of location(s) where the intervention occurred, including any necessary infrastructure or relevant features.	The educational sessions in both groups were held at the health centers
**WHEN and HOW MUCH**<br>Describe the number of times the intervention was delivered and over what period of time including	Eight sessions of training, each lasting 30–45 minutes<br>Study length N/R

the number of sessions, their schedule, and their duration, intensity or dose.	
**TAILORING**<br>If an intervention was planned to be personalised, titrated or adapted, then describe what, why, when, and how.	N/R
**MODIFICATION**<br>If an intervention was modified during the course of the study, describe the changes (what, why, when, and how).	N/R
**HOW WELL**<br>Planned: If intervention adherence or fidelity was assessed, describe how and by whom, and if any strategies were used to maintain or improve fidelity, describe them.	N/R
Actual: If intervention adherence or fidelity was assessed, describe the extent to which the intervention was delivered as planned.	N/R

Hernandez, 2021	
**TIDiER Tool Item**	**Main Paper**
**BRIEF NAME**<br>Provide the name or a phrase that describes the intervention.	MoPoTsyo
**WHY**<br>Describe any rationale, theory, or goal of the elements essential to the intervention.	MoPoTsyo applies a unique healthcare model that utilizes Peer Educators (PEs), patients with diabetes and/or hypertension themselves, to return to their local villages to screen and initiate management of fellow community members. Our article expands on these clinical outcomes to include data until 2016 (a total of 8 years), and additionally to describe the long-term retention of patients, which is an important element of its long-term effectiveness.<br>*related to Taniguchi et al
**WHAT**<br>Materials: Describe any physical or informational materials used in the intervention, including those provided to participants or used in intervention delivery or in training of intervention providers. Provide information on where the materials can be accessed (e.g., online appendix, URL).	N/R
Procedures: Describe each of the procedures, activities, and/or processes used in the intervention, including any enabling or support activities.	MoPoTsyo uses a community-centred approach to long-term care for diabetes and hypertension management. PEs carry out screening of people aged 40 years and over. Only patients with BG or pressure results above these thresholds are enrolled in the program, all other adults in the household are advised to contact the peer educator if they notice symptoms of diabetes. The programme itself provides laboratory services and operates an RDF for vetted, reliable and lower-priced drugs supplied to contracted pharmacies. PEs act as an intermediary between the physician and hard to reach patients to deliver and explain blood results, pick-up prescriptions and monitor blood sugars and pressures.
**WHO PROVIDED**<br>For each category of intervention provider (e.g., psychologist, nursing assistant), describe their expertise, background and any specific training given.	PEs, patients selected for their motivation, are trained to screen and manage these conditions in their local village and health centre catchment area.
**HOW**<br>Describe the modes of delivery (e.g., face-to-face or by some other mechanism, such as internet or telephone) of the intervention and whether it was provided individually or in a group.	Face to face, individual
**WHERE**<br>Describe the type(s) of location(s) where the intervention occurred, including any necessary infrastructure or relevant features.	N/R
**WHEN and HOW MUCH**<br>Describe the number of times the intervention was delivered and over what period of time including the number of sessions, their schedule, and their	N/R

duration, intensity or dose.	
**TAILORING**<br>If an intervention was planned to be personalised, titrated or adapted, then describe what, why, when, and how.	Planned to be personalised at individual level
**MODIFICATION**<br>If an intervention was modified during the course of the study, describe the changes (what, why, when, and how).	N/R
**HOW WELL**<br>Planned: If intervention adherence or fidelity was assessed, describe how and by whom, and if any strategies were used to maintain or improve fidelity, describe them.	N/R
Actual: If intervention adherence or fidelity was assessed, describe the extent to which the intervention was delivered as planned.	N/R

Ju, 2018	
**TIDiER Tool Item**	**Main Paper**
**BRIEF NAME**<br>Provide the name or a phrase that describes the intervention.	Effect of peer support on diabetes distress
**WHY**<br>Describe any rationale, theory, or goal of the elements essential to the intervention.	To investigate whether peer support would reduce diabetes distress and improve glycaemic control when added to usual diabetes education among adults with Type 2 diabetes in China.
**WHAT**<br>Materials: Describe any physical or informational materials used in the intervention, including those provided to participants or used in intervention delivery or in training of intervention providers. Provide information on where the materials can be accessed (e.g., online appendix, URL).	Participants in the usual education group used other resources such as newspapers, networks, or other medical institutions.
Procedures: Describe each of the procedures, activities, and/or processes used in the intervention, including any enabling or support activities.	Usual education consisted of 2 h each month of focused diabetes education. A variety of diabetes self-management interventionists including physicians, certified diabetes educators, dieticians, psychologists and podiatric nurses led individual sessions. Participants also used other resources such as newspapers, networks or other medical institutions to gain information about diabetes self-management.<br><br>Peer leaders guided participants to carry out activities with the help of community health centres or medical volunteers. The activities included themed and non-themed activities.<br>Themed activities reviewed diabetes knowledge and skills at least once a month. An important part of this self-management support was teaching and reinforcing self-management skills. Guided by peer leaders, participants discussed and shared a variety of skills, including healthy meal planning, food preparation, blood glucose monitoring, medication management and physical activities. Peer leaders encouraged participants to communicate and share experience with each other. The leaders also worked with participants to apply knowledge and skills in practice, such as setting or achieving goals, solving problems and overcoming barriers.<br>Non-themed activities included informal communication among participants through home visits, telephone, e-mails and so on. These were facilitated by the peer support with usual education being organized through community health centres serving individual communities. Many participants knew each other and had frequent occasion to meet each other informally within their communities; thus, there was a variety of ways to increase knowledge about diabetes and self-management for peers, such as during tai chi and open-air fitness dancing sessions.
**WHO PROVIDED**<br>For each category of intervention provider (e.g.,	Peer leaders were chosen based on residence, demographics and other characteristics, including

psychologist, nursing assistant), describe their expertise, background and any specific training given.	interpersonal skills evident in interviews, time available and willingness to cooperate as part of a team and follow study protocols.<br><br>Usual care provided by physicians, certified diabetes educators, dieticians, psychologists and podiatric nurses
**HOW**<br>Describe the modes of delivery (e.g., face-to-face or by some other mechanism, such as internet or telephone) of the intervention and whether it was provided individually or in a group.	Face to face, group and individual<br>Telephone and emails, individually
**WHERE**<br>Describe the type(s) of location(s) where the intervention occurred, including any necessary infrastructure or relevant features.	Eight community health centres in the Xuanwu district in Nanjing were selected, each serving a defined community from which peer leaders and participants were recruited. The study team coordinated with the staff members of each community health centre to implement and monitor the study.
**WHEN and HOW MUCH**<br>Describe the number of times the intervention was delivered and over what period of time including the number of sessions, their schedule, and their duration, intensity or dose.	Usual education consisted of 2 h each month for 12 months<br>Peer support occurred for 12 months meeting at least once a month<br><br>12 month study
**TAILORING**<br>If an intervention was planned to be personalised, titrated or adapted, then describe what, why, when, and how.	Designed to be personalized
**MODIFICATION**<br>If an intervention was modified during the course of the study, describe the changes (what, why, when, and how).	N/R
**HOW WELL**<br>Planned: If intervention adherence or fidelity was assessed, describe how and by whom, and if any strategies were used to maintain or improve fidelity, describe them.	N/R
Actual: If intervention adherence or fidelity was assessed, describe the extent to which the intervention was delivered as planned.	N/R

Khan, 2018	
**TIDiER Tool Item**	**Main Paper**
**BRIEF NAME**<br>Provide the name or a phrase that describes the intervention.	Peer-led DSME
**WHY**<br>Describe any rationale, theory, or goal of the elements essential to the intervention.	Peer-led diabetes self-management program was used in this pilot study for determining the feasibility of this program among Bangladeshi people with type 2 diabetes. The aim of the study was to evaluate the effectiveness of a diabetes education program guided by health professionals versus peers in improving diabetes care among people with type 2 diabetes.
**WHAT**<br>Materials: Describe any physical or informational materials used in the intervention, including those provided to participants or used in intervention delivery or in training of intervention providers. Provide information on where the materials can be accessed (e.g., online appendix, URL).	Leaflets, a flip-chart, and posters
Procedures: Describe each of the procedures, activities, and/or processes used in the intervention, including any enabling or support activities.	Sixty-seven patients led by the health professionals were divided to four groups, and each group was directed by a professional. Sixty-six patients guided by peer educators were also divided to four groups, and each group was led by two peer educators. All the patients (both professionals and peer educator groups) attended a two-hour diabetes education program once at the time of their enrollment, following a predesigned curriculum. The first interview of the patients was taken before attending the education session.<br><br>The education program was followed by face-to-face and group discussions (using leaflets, a flip-chart, and posters) for any problems they faced, thereby allowing them to freely discuss general management of diabetes.
**WHO PROVIDED**<br>For each category of intervention provider (e.g., psychologist, nursing assistant), describe their expertise, background and any specific training given.	Peers or health professionals<br>Peers: Twenty-six diabetes patients attended the program and eight peer educators (four males and four females), who had diabetes at least for five years were selected (age > 40 years, HbA1c < 7%, graduation in education, committed to training and willing to spend sufficient time, enthusiastic to be peer educators, and residing in Dhaka city).<br><br>Training: The diabetes education trainer program team of BIRDEM was invited to send four trainers for conducting a three-day training program for professionals and peers. They were requested to evaluate the performance of health professionals and peer educators. The trainers conducted pre- and post-training assessments of the professionals and peers. The trainers were briefed about the background and objectives of the workshop. They reviewed the existing curriculum of the health educator training program, keeping in mind the Funnell's education

	scheme.
**HOW**<br>Describe the modes of delivery (e.g., face-to-face or by some other mechanism, such as internet or telephone) of the intervention and whether it was provided individually or in a group.	Face to face, group
**WHERE**<br>Describe the type(s) of location(s) where the intervention occurred, including any necessary infrastructure or relevant features.	N/R
**WHEN and HOW MUCH**<br>Describe the number of times the intervention was delivered and over what period of time including the number of sessions, their schedule, and their duration, intensity or dose.	one two-hour session<br>12 week study
**TAILORING**<br>If an intervention was planned to be personalised, titrated or adapted, then describe what, why, when, and how.	Free discussion of problems in group meeting
**MODIFICATION**<br>If an intervention was modified during the course of the study, describe the changes (what, why, when, and how).	N/R
**HOW WELL**<br>Planned: If intervention adherence or fidelity was assessed, describe how and by whom, and if any strategies were used to maintain or improve fidelity, describe them.	N/R
Actual: If intervention adherence or fidelity was assessed, describe the extent to which the intervention was delivered as planned.	N/R

Khetan, 2019	
**TIDiER Tool Item**	**Main Paper**
**BRIEF NAME**<br>Provide the name or a phrase that describes the intervention.	Project SEHAT (Study to Enhance Heart Associated Treatments)
**WHY**<br>Describe any rationale, theory, or goal of the elements essential to the intervention.	Use of CHWs to manage hypertension, diabetes, and smoking in an integrated manner would result in improved control of these risk factors, compared with a control group.
**WHAT**<br>Materials: Describe any physical or informational materials used in the intervention, including those provided to participants or used in intervention delivery or in training of intervention providers. Provide information on where the materials can be accessed (e.g., online appendix, URL).	CHWs had a flipbook aid that summarized these strategies and provided a template for discussion. As many of our patients were illiterate, the patient facing side of the flipbook only had pictures, with all textual information conveyed verbally.
Procedures: Describe each of the procedures, activities, and/or processes used in the intervention, including any enabling or support activities.	CHWs provided home based counseling to people with hypertension. This usually lasted for around an hour and consisted of a behavior change strategy focused on modifying the individual’s lifestyle (diet and physical activity), improving health care seeking behavior, and addressing barriers to medication adherence.<br>Importantly, in addition to lifestyle modifications, CHWs specifically focused on encouraging physician visits, medication purchase, and medication adherence. The communication was conducted in the native language of the participant.<br>Six months after the start of the hypertension intervention, CHWs underwent training for diabetes and started visiting patients with diabetes. These visits followed a similar format and frequency as hypertension, and a separate flipbook was provided for diabetes counseling.<br>Two months after the start of the diabetes intervention, CHWs received training for the smoking intervention and started visiting patients who smoked, aided by a flipbook. However, the frequency and nature of visits for smoking were customized depending on whether the participant was contemplative or pre-contemplative about quitting smoking. More intensive support was provided to participants who were contemplative about quitting smoking. Once all the 3 interventions were underway, CHWs continued to follow all participants under their care until the end of the study. For a patient with multiple risk factors (e.g., hypertension and diabetes), CHW visits at the start of the study focused on hypertension, and after undergoing diabetes training at the 6-month mark, CHWs also began to counsel the patient on diabetes (while continuing the hypertension intervention).
**WHO PROVIDED**<br>For each category of intervention provider (e.g., psychologist, nursing assistant), describe their	Community health workers (CHWs) are lay individuals who undergo brief periods of training, usually aimed at a specific health task or disease. Unlike other nonphysician

expertise, background and any specific training given.	health workers such as nurses or pharmacists, CHWs typically do not have formal health care degrees and work in the community setting, outside the traditional health care system. All CHWs, field workers and supervisors were recruited for the purpose of the study and were not previously a part of the health system. The training of the CHWs was also staggered, with initial training and work focused on hypertension, followed by diabetes and then smoking. Training for each risk factor was delivered over 1 to 2 weeks (3 h/day). All CHWs were retained from the start to the end of the intervention, with zero attrition
**HOW**<br>Describe the modes of delivery (e.g., face-to-face or by some other mechanism, such as internet or telephone) of the intervention and whether it was provided individually or in a group.	face to face, individual
**WHERE**<br>Describe the type(s) of location(s) where the intervention occurred, including any necessary infrastructure or relevant features.	a single site in Dalkhola, India<br>Dalkhola is a semiurban town in the state of West Bengal, with an approximate population of 20,000 individuals and an agriculture-based economy. The town is located in the district of Uttar Dinajpur, which in the 2011 census had a literacy rate of 60%, well below the national average of 74%. The town has a single government primary health center, with no secondary or tertiary health care facility. In addition to the single primary health center, health care is provided by private practitioners. The study coordinating center was at University Hospitals, Case Western Reserve University, in Cleveland, Ohio
**WHEN and HOW MUCH**<br>Describe the number of times the intervention was delivered and over what period of time including the number of sessions, their schedule, and their duration, intensity or dose.	CHWs visited patients with hypertension or diabetes every 2 months till the end of the study (2 year study period). Visits were 1hr<br>smoking visits were customized
**TAILORING**<br>If an intervention was planned to be personalised, titrated or adapted, then describe what, why, when, and how.	Designed to be tailored
**MODIFICATION**<br>If an intervention was modified during the course of the study, describe the changes (what, why, when, and how).	N/R
**HOW WELL**<br>Planned: If intervention adherence or fidelity was assessed, describe how and by whom, and if any strategies were used to maintain or improve fidelity, describe them.	CHWs had a template to use for their encounter, which was focused on reinforcing previous recommendations, understanding barriers to behavior change, behavior change communication, and problem solving. Each CHW had a paper diary in which they recorded details of their patient encounters, in a predefined format.<br>To provide supervision and support to CHWs, 1 supervisor was appointed for every 3 CHWs. The supervisors randomly verified 10% of the work done by CHWs every 2 months, following a standard protocol,

	which varied according to patient and trial progress.
Actual: If intervention adherence or fidelity was assessed, describe the extent to which the intervention was delivered as planned.	N/R

Latina, 2020	
**TIDiER Tool Item**	**Main Paper**
**BRIEF NAME**<br>Provide the name or a phrase that describes the intervention.	Grenada Heart Project–Community Health ActioN to EncouraGe healthy BEhaviors (GHPCHANGE):
**WHY**<br>Describe any rationale, theory, or goal of the elements essential to the intervention.	The call to address the increasing prevalence of noncommunicable diseases worldwide motivated the development of the Grenada Heart Project. The GHP-CHANGE was developed to extend the findings in Spain to a LMIC and study the effects of peer education groups on their CV risk factors, quality of life, and health-related behaviors.
**WHAT**<br>Materials: Describe any physical or informational materials used in the intervention, including those provided to participants or used in intervention delivery or in training of intervention providers. Provide information on where the materials can be accessed (e.g., online appendix, URL).	All eligible participants were invited to attend an intense educational lecture series. They were required to participate in at least three workshops in order to enroll in the study. The workshop themes included motivation to change, physical activity, healthy diet, smoking cessation, blood pressure, and stress management. The research team, local experts, and respected community members presented a general overview of the lifestyle intervention and participants had an opportunity to ask questions. Upon consenting, participants received a blank notebook and health literacy materials/brochures provided by the American Heart Association such Easy Food Tips for Heart-Healthy Eating (adapted for Grenadian diet), Just Move, Controlling Your Risk Factors, and Understanding and Controlling High Blood Pressure. These materials can be accessed online at the American Heart Association Website (https://www.heart.org/en/health-topics/consumer-healthcare/order-american-heart-associationeducational-brochures). The educational materials aimed to promote management of risk factors and the notebook provides a means of recording lifestyle behaviors, such as health goals, blood pressure values, and eating habits.
Procedures: Describe each of the procedures, activities, and/or processes used in the intervention, including any enabling or support activities.	The framework for the Grenada Heart Project has been based on the peer group-based lifestyle intervention in Spain, as reported previously. The intervention group was organized into groups of 8–12 individuals in their local parish. A “peer leader” was a community lay-person selected from motivated individuals willing to undergo additional training from the research staff to moderate the peer groups and take attendance at group meetings. The peer group meetings were planned to meet monthly for 1 year. The peer group leaders were educated using evidence-based guidelines and encouraged to promote 150 minutes weekly of physical activity, consumption of at least five fruits and vegetables daily, smoking cessation, and blood pressure management.11-15 Peer leaders were provided topics to discuss at the monthly meetings, such as low salt diet and hypertension, diabetes prevention, coping strategies for stress, and smoking cessation; along a blood pressure machine with

	two different cuff sizes.
**WHO PROVIDED**<br>For each category of intervention provider (e.g., psychologist, nursing assistant), describe their expertise, background and any specific training given.	A "peer leader" was a community lay-person selected from motivated individuals willing to undergo additional training from the research staff to moderate the peer groups and take attendance at group meetings. The leaders underwent an additional three-hour training session on leadership and communication skills in addition to the relevant healthy behavior promotion. The peer group leaders were educated using evidence-based guidelines.
**HOW**<br>Describe the modes of delivery (e.g., face-to-face or by some other mechanism, such as internet or telephone) of the intervention and whether it was provided individually or in a group.	Face to face, group
**WHERE**<br>Describe the type(s) of location(s) where the intervention occurred, including any necessary infrastructure or relevant features.	local parish<br>five parishes around the island: the parishes of St David's, St Andrew's, St George's, St John's, and St Mark's.
**WHEN and HOW MUCH**<br>Describe the number of times the intervention was delivered and over what period of time including the number of sessions, their schedule, and their duration, intensity or dose.	The peer group meetings were planned to meet monthly for 1 year
**TAILORING**<br>If an intervention was planned to be personalised, titrated or adapted, then describe what, why, when, and how.	planned to be personalised<br>Leaders were able to adapt themes for each meeting to their particular interest. For example if they did not have any smokers in their group, they could skip the 'smoking cessation' group meeting.
**MODIFICATION**<br>If an intervention was modified during the course of the study, describe the changes (what, why, when, and how).	N/R
**HOW WELL**<br>Planned: If intervention adherence or fidelity was assessed, describe how and by whom, and if any strategies were used to maintain or improve fidelity, describe them.	The Project administrator in Grenada made routine visits to each group to encourage and monitor the attendance, and to receive feedback from the group leader
Actual: If intervention adherence or fidelity was assessed, describe the extent to which the intervention was delivered as planned.	N/R

Liu, 2020	
**TIDiER Tool Item**	**Main Paper**
**BRIEF NAME**<br>Provide the name or a phrase that describes the intervention.	Peer support in Shanghai's Commitment to diabetes
**WHY**<br>Describe any rationale, theory, or goal of the elements essential to the intervention.	Here, we report staging peer support (PS) for diabetes within the context of a systematic approach to integrating community, specialty, and hospital care. Healthy China 2030 promotes a shift from disease treatment to health promotion and health management, requiring engagement from all sectors in society
**WHAT**<br>Materials: Describe any physical or informational materials used in the intervention, including those provided to participants or used in intervention delivery or in training of intervention providers. Provide information on where the materials can be accessed (e.g., online appendix, URL).	Final training outlines and all other intervention materials described in this paper are available at http://peersforprogress.org/who-we-are/ collaborative-projects/shanghai-integration-model/
Procedures: Describe each of the procedures, activities, and/or processes used in the intervention, including any enabling or support activities.	Informal activities led by PLs ranged from weekly to monthly across all CHCs. Interactions included face-to-face informal gathering, phone calls or WeChat, and WeChat groups to provide ongoing support. Additionally, some CHCs worked with CSMGs or Residential Committees to develop outdoor activities (e.g., field trips), and some encouraged PLs and participants to attend activities organized by Residential Committees such as exercise groups.
**WHO PROVIDED**<br>For each category of intervention provider (e.g., psychologist, nursing assistant), describe their expertise, background and any specific training given.	CHC staff recruited peer leaders based on existing relationships with people in the community. Several CHCs recruited peer leaders who had experience working on prior health projects from Community Self-Management Groups (CSMGs) or from Residential Committees.<br><br>Initial training in February 2017 spanned two and one-half days and included CHC staff and PLs together. Subsequent, 6-h training in August 2017 included an additional 15 PLs along with those from initial training. Training covered diabetes knowledge, diabetes self-care, communication skills, group skills, program protocols, experience sharing, practical tips, as well as key core messages, group facilitation skills, and helping patients make the transition from discussing problems to taking action using a "Diabetes Action Plan" as a framework.<br><br>An additional follow-up training for all PLs in June 2018 reviewed PL activities and skills.
**HOW**<br>Describe the modes of delivery (e.g., face-to-face or by some other mechanism, such as internet or telephone) of the intervention and whether it was	Face to face and virtual; group and individual via technology/social messaging

provided individually or in a group.	
**WHERE**<br>Describe the type(s) of location(s) where the intervention occurred, including any necessary infrastructure or relevant features.	community health centers (CHCs)<br>Ten CHCs were selected according to their having in place the key organizational resources and protocols of the SIM and their willingness to collaborate in the project.
**WHEN and HOW MUCH**<br>Describe the number of times the intervention was delivered and over what period of time including the number of sessions, their schedule, and their duration, intensity or dose.	monthly group classes, exercises, gatherings<br>virtual group discussions<br><br>unclear number of sessions/interactions, timing, duration, etc.<br><br>Training spanned two and one-half days and included CHC staff and PLs together. Subsequent, 6-h training in August 2017
**TAILORING**<br>If an intervention was planned to be personalised, titrated or adapted, then describe what, why, when, and how.	Individual follow up or meetings with those needing or requesting extra support
**MODIFICATION**<br>If an intervention was modified during the course of the study, describe the changes (what, why, when, and how).	Services and activities PLs implemented as revised prior to the program's initiation in February 2017 and further revised based on feedback from CHCs, peer leaders, and participants through the course of implementation.<br><br>Group activities came to focus on group goal-setting while encouraging individualized goals for those who choose them. To supplement those identified by CHC staff, research staff provided additional resources on self-management topics, including, for example, a diabetes patient magazine and two popular books on diabetes edited by one of the present authors, Professor Jia. Additionally, "Patient Insulin Stories" collected from program participants were used to encourage appropriate insulin therapy
**HOW WELL**<br>Planned: If intervention adherence or fidelity was assessed, describe how and by whom, and if any strategies were used to maintain or improve fidelity, describe them.	N/R
Actual: If intervention adherence or fidelity was assessed, describe the extent to which the intervention was delivered as planned.	N/R

Mwakalinga 2021	
**TIDiER Tool Item**	**Main Paper**
**BRIEF NAME**<br>Provide the name or a phrase that describes the intervention.	Evaluation of diabetic peer support
**WHY**<br>Describe any rationale, theory, or goal of the elements essential to the intervention.	The aim of this study is to assess and evaluate the Kamuzu Central Hospital (KCH) diabetic peer support program's (DPSP) impact 4 years after its establishment by assessing knowledge, self-efficacy and behaviours of DPSP members compared to non-members.
**WHAT**<br>Materials: Describe any physical or informational materials used in the intervention, including those provided to participants or used in intervention delivery or in training of intervention providers. Provide information on where the materials can be accessed (e.g., online appendix, URL).	N/A
Procedures: Describe each of the procedures, activities, and/or processes used in the intervention, including any enabling or support activities.	Cross-sectional descriptive study using self and interviewer-administered questionnaires. The survey questions were reviewed by the diabetes peer support trainer, lead supporters as well as clinicians at the diabetes clinic (available in the appendix). Patients self-identified whether they belonged to the diabetes peer support program or not.
**WHO PROVIDED**<br>For each category of intervention provider (e.g., psychologist, nursing assistant), describe their expertise, background and any specific training given.	N/A
**HOW**<br>Describe the modes of delivery (e.g., face-to-face or by some other mechanism, such as internet or telephone) of the intervention and whether it was provided individually or in a group.	N/R
**WHERE**<br>Describe the type(s) of location(s) where the intervention occurred, including any necessary infrastructure or relevant features.	The study was done at Kamuzu Central Hospital. KCH is the main government referral hospital for the central region of the country.
**WHEN and HOW MUCH**<br>Describe the number of times the intervention was delivered and over what period of time including the number of sessions, their schedule, and their duration, intensity or dose.	N/A
**TAILORING**<br>If an intervention was planned to be personalised, titrated or adapted, then describe what, why, when, and how.	N/A
**MODIFICATION**<br>If an intervention was modified during the course of the study, describe the changes (what, why, when, and how).	N/R
**HOW WELL**<br>Planned: If intervention adherence or fidelity was	N/R

assessed, describe how and by whom, and if any strategies were used to maintain or improve fidelity, describe them.	
Actual: If intervention adherence or fidelity was assessed, describe the extent to which the intervention was delivered as planned.	N/R

Paz-Pacheco, 2017	
**TIDiER Tool Item**	**Main Paper**
**BRIEF NAME**<br>Provide the name or a phrase that describes the intervention.	community-based diabetes self-management education (DSME) program in a rural agricultural setting
**WHY**<br>Describe any rationale, theory, or goal of the elements essential to the intervention.	To assess the effectiveness of a community-based DSME program in improving anthropometric, biochemical, and health behavior outcomes among persons with diabetes.
**WHAT**<br>Materials: Describe any physical or informational materials used in the intervention, including those provided to participants or used in intervention delivery or in training of intervention providers. Provide information on where the materials can be accessed (e.g., online appendix, URL).	N/R
Procedures: Describe each of the procedures, activities, and/or processes used in the intervention, including any enabling or support activities.	Participants in both groups were given oral advice on diet, exercise, foot care, and medication compliance on each follow-up visit. The participants in the intervention group additionally received DSME as described here. Modules developed by the International Diabetes Federation (IDF) Consultative Section on Diabetes Education (DECS) in 2002 were translated into Filipino by the Sentrong Wikang Filipino (Center of the Philippine Language) of our university. The translated modules were modified according to the participants' level of knowledge. There were eight modules in the DSME program: (1) overview of diabetes mellitus, (2) diabetes and exercise, (3) diabetes and diet, (4) pharmacologic treatment of diabetes, (5) insulin use, (6) acute complications of diabetes, (7) microvascular and macrovascular complications of diabetes, and (8) foot care.<br>These modules were delivered by peer educators with visual aids followed by group discussions. The teaching sessions were held in the village health centers, with six to 15 participants in attendance in each session. Local venues are good settings for DSME interventions because the educator can address issues that can be more difficult to deal with in the clinical setting, such as cultural, family, and environmental factors affecting lifestyle and barriers to optimal self-care. Two modules were taught per session. Each of the four weekly sessions lasted for ~1 h.
**WHO PROVIDED**<br>For each category of intervention provider (e.g., psychologist, nursing assistant), describe their expertise, background and any specific training given.	In total, 14 volunteer peer educators were recruited among the participants. There was no educational attainment or profession that was required of the peer educators; only the willingness to be trained and eventually share the knowledge to others.<br>Among the peer educators were a village leader (barangay chairman), a retired school principal, a village health worker, a village nutrition scholar, a jeepney driver, whereas the rest were housewives.

	They attended a two-day workshop during which they received a course manual that described both the course content and process on how to teach them. Endocrinologists from our group conducted the workshop. After the peer educators were trained, they were asked to do a return demonstration.
**HOW**<br>Describe the modes of delivery (e.g., face-to-face or by some other mechanism, such as internet or telephone) of the intervention and whether it was provided individually or in a group.	Face to face, group
**WHERE**<br>Describe the type(s) of location(s) where the intervention occurred, including any necessary infrastructure or relevant features.	The municipality of San Juan in the province of Batangas in the Philippines was the setting of this study. It is a rural agricultural town 120 km south of Manila. It has one municipal health officer, three rural health physicians, and nine municipal public health nurses serving a population of around 80 000 people. The teaching sessions were held in the village health centers,
**WHEN and HOW MUCH**<br>Describe the number of times the intervention was delivered and over what period of time including the number of sessions, their schedule, and their duration, intensity or dose.	8 modules taught across 4 weeks. 1 session per week lasting about 1 hr in duration
**TAILORING**<br>If an intervention was planned to be personalised, titrated or adapted, then describe what, why, when, and how.	N/R
**MODIFICATION**<br>If an intervention was modified during the course of the study, describe the changes (what, why, when, and how).	N/R
**HOW WELL**<br>Planned: If intervention adherence or fidelity was assessed, describe how and by whom, and if any strategies were used to maintain or improve fidelity, describe them.	N/R
Actual: If intervention adherence or fidelity was assessed, describe the extent to which the intervention was delivered as planned.	N/R

Peimani, 2018	
**TIDiER Tool Item**	**Main Paper**
**BRIEF NAME**<br>Provide the name or a phrase that describes the intervention.	Peer support intervention in patients with type 2 diabetes
**WHY**<br>Describe any rationale, theory, or goal of the elements essential to the intervention.	This study aims to assess the effectiveness of a peer support intervention, in which patients with T2DM were provided ongoing self-management support by trained peers with diabetes directed at improving self-care behaviors, self-efficacy and life quality.
**WHAT**<br>Materials: Describe any physical or informational materials used in the intervention, including those provided to participants or used in intervention delivery or in training of intervention providers. Provide information on where the materials can be accessed (e.g., online appendix, URL).	Illustrated educational materials and virtual clinic website (http://emri.tums.ac.ir/vclinic) were given to each participant.<br>Diaries for participants to write brief reports<br>Peers were guided by a detailed manual
Procedures: Describe each of the procedures, activities, and/or processes used in the intervention, including any enabling or support activities.	All participants in both groups received usual education. The first session included an overview of type 2 diabetes (symptoms of hyper and hypoglycemia, blood glucose self-monitoring and the importance of patients' active participation in their plan of care). In the second session, main focus was on increasing patients' awareness of the importance of a healthy diet and weight reduction. The third session explained health benefits of regular physical activity and exercise. And finally, during the fourth session, they were taught how to manage the ABCs of diabetes (HbA1c, Blood pressure, Cholesterol). At the final session, participants were randomly allocated to either peer support group or control group<br><br>The intervention group was subdivided into ten groups, each comprising ten persons and randomly paired with one of the trained peers.<br><br>In the first group session, the participants discussed their problems and concerns which had affected their adherence to the medications. In the second session, they discussed their views of the difficulties of complying with diabetic diet and perceptions about their obesity risk and weight control. In the third group meeting, they discussed their common problems and possible solutions for being physically active and doing exercise. In the fourth session, discussion centred around the impacts of chronic illnesses like diabetes on family relationships and patients' expectations regarding compassion and support. The main focus of the fifth session was on patients' fears, worries and concerns about the future and living with diabetes and its chronic complications. And the last session was devoted to feelings of depression, hopelessness and anxiety in everyday self-care activities with the

	emphasis on the strategies for dealing with and overcoming these challenges.<br>In each session, participants discussed and shared their experiences and challenges of diabetes management with each other (two by two) and with the group members and supported each other to set and achieve their goals. Peer encouraged all group members to speak and actively participate in discussions and did not allow participants go outside the mainstream of discussions.<br>After each group meeting, peers were requested to review the recordings of their monthly meetings based on the manual.<br>Peers had telephone contacts weekly with their 10 patients. The aim of these contacts, which were mostly unstructured and individualized, was to provide continuing social, emotional and behavioral support and to help the patients on how to apply their diabetes knowledge in everyday life based on the peer's personal experience and to discuss the practical issues arising from living with diabetes. Peers were allowed to make more contacts with their patients if needed. During the intervention, peers were provided with diaries to write brief reports.
**WHO PROVIDED**<br>For each category of intervention provider (e.g., psychologist, nursing assistant), describe their expertise, background and any specific training given.	Peers with the following criteria<br>Patients' knowledge on the basis of excellent diabetes control (HbA1c < 8.5%). Patients with good interpersonal skills and qualities (e.g., patients who enjoy contact with others, personable). Patients who demonstrate flexibility, self-motivation and good problem-solving skills. Patients with good active and non-judgmental listening skills. Patients who have had type 2 diabetes. Patients who are able to read and write and attend a 3-day course. peers received a 3-day structured, buzzgroup interactive course developed and conducted by the study team. Table 1 presents a summary of the content of the course.<br><br>Usual education provided by a credentialed diabetes educator
**HOW**<br>Describe the modes of delivery (e.g., face-to-face or by some other mechanism, such as internet or telephone) of the intervention and whether it was provided individually or in a group.	Face to face, group<br>Telephone, individual
**WHERE**<br>Describe the type(s) of location(s) where the intervention occurred, including any necessary infrastructure or relevant features.	Patients with T2DM were recruited through a diabetes specialty clinic of the Endocrinology and Metabolism Research Institute affiliated to Tehran University of Medical Sciences. All patients who come to this clinic are routinely under supervision of endocrinologists, dieticians, diabetes nurse educators and qualified psychologists if needed.

	The study took place in a university specialty clinic in the city of Tehran in which people with diabetes receive state-of-the-art medical care, patient education, in addition to services for the prevention and management of complications
**WHEN and HOW MUCH**<br>Describe the number of times the intervention was delivered and over what period of time including the number of sessions, their schedule, and their duration, intensity or dose.	All participants received four weekly sessions (90 min each)<br>Intervention group received 1 group meeting each month for 6 months lasting up to 2 hours and scheduled weekly telephone calls<br>Study duration of 6 months
**TAILORING**<br>If an intervention was planned to be personalised, titrated or adapted, then describe what, why, when, and how.	Peers had regular scheduled telephone contacts weekly with their 10 patients. The aim of these contacts, which were mostly unstructured and individualized
**MODIFICATION**<br>If an intervention was modified during the course of the study, describe the changes (what, why, when, and how).	N/R
**HOW WELL**<br>Planned: If intervention adherence or fidelity was assessed, describe how and by whom, and if any strategies were used to maintain or improve fidelity, describe them.	N/R
Actual: If intervention adherence or fidelity was assessed, describe the extent to which the intervention was delivered as planned.	N/R

Rao, 2020	
**TIDiER Tool Item**	**Main Paper**
**BRIEF NAME**<br>Provide the name or a phrase that describes the intervention.	MoPoTsyo
**WHY**<br>Describe any rationale, theory, or goal of the elements essential to the intervention.	However, MoPoTsyo participants do not uniformly use all four program services, and the relative association of each service with glycemic control has never been assessed. Our aims were to 1) quantify MoPoTsyo participant utilization of each program component and 2) define the relationship between each program component and glycemic control. This analysis may not only reveal opportunities for improvement in MoPoTsyo's population but also advance a more nuanced understanding of the benefit of peer educator programs in resource-poor settings.<br><br>*related to Taniguchi et al
**WHAT**<br>Materials: Describe any physical or informational materials used in the intervention, including those provided to participants or used in intervention delivery or in training of intervention providers. Provide information on where the materials can be accessed (e.g., online appendix, URL).	N/R
Procedures: Describe each of the procedures, activities, and/or processes used in the intervention, including any enabling or support activities.	Once enrolled, participants with diabetes may attend group sessions—typically monthly—hosted by peer educators in their homes for disease monitoring (point-of-care glucose, blood pressure, and weight), self-management education, and support. Peer educators inform MoPoTsyo participants of the scheduled dates that a physician will be providing consultations in their area. MoPoTsyo has established a Revolving Drug Fund to provide lowcost diabetes and hypertension medications to its members. MoPoTsyo purchases 17 medications in bulk on the international market and sells them to local private and public pharmacies. MoPoTsyo then requires these pharmacies to sell these medications to MoPoTsyo members at a fixed published price per tablet established by MoPoTsyo.
**WHO PROVIDED**<br>For each category of intervention provider (e.g., psychologist, nursing assistant), describe their expertise, background and any specific training given.	MoPoTsyo selects peer educators among community members with diabetes based on literacy, motivation, and social aptitude. Each peer educator candidate undergoes a six-week training course developed by physicians, pharmacists, and experienced peer educators. This course aims to teach peer educators about the biology of diabetes as well as key components of monitoring and treatment. At the end of the training course, candidates must pass an exam in order to become qualified MoPoTsyo peer educators. Peer educators return to their communities and perform house-to-house diabetes screening.
**HOW**	Face to face, group

Describe the modes of delivery (e.g., face-to-face or by some other mechanism, such as internet or telephone) of the intervention and whether it was provided individually or in a group.	
**WHERE**<br>Describe the type(s) of location(s) where the intervention occurred, including any necessary infrastructure or relevant features.	N/R
**WHEN and HOW MUCH**<br>Describe the number of times the intervention was delivered and over what period of time including the number of sessions, their schedule, and their duration, intensity or dose.	Monthly visits to community members
**TAILORING**<br>If an intervention was planned to be personalised, titrated or adapted, then describe what, why, when, and how.	N/R
**MODIFICATION**<br>If an intervention was modified during the course of the study, describe the changes (what, why, when, and how).	N/R
**HOW WELL**<br>Planned: If intervention adherence or fidelity was assessed, describe how and by whom, and if any strategies were used to maintain or improve fidelity, describe them.	N/R
Actual: If intervention adherence or fidelity was assessed, describe the extent to which the intervention was delivered as planned.	N/R

Rotheram-Borus, 2012	
**TIDiER Tool Item**	**Main Paper**
**BRIEF NAME**<br>Provide the name or a phrase that describes the intervention.	Diabetes Buddies
**WHY**<br>Describe any rationale, theory, or goal of the elements essential to the intervention.	Feasibility and acceptability of a mobile phone–based peer support intervention among women in resource-poor settings to self-manage their diabetes. Secondary goals were to evaluate the intervention's effectiveness to motivate diabetes-related health choices.<br><br>We adapted the Power to Prevent program, an evidence-based intervention targeted to African Americans in the United States, into a format of peer support suited for delivery by NGOs in South African townships
**WHAT**<br>Materials: Describe any physical or informational materials used in the intervention, including those provided to participants or used in intervention delivery or in training of intervention providers. Provide information on where the materials can be accessed (e.g., online appendix, URL).	Food diaries of residents
Procedures: Describe each of the procedures, activities, and/or processes used in the intervention, including any enabling or support activities.	The intervention had 3 components: (1) a series of 12 psychoeducational group sessions that address improving one's lifestyle of eating, exercising, and abstaining from alcohol and drugs; (2) mobile phone probes that ask about health daily; and (3) text messaging to a buddy to support lifestyle changes.<br>Our program, called Diabetes Buddies, offered a laddered system of peer support. The program paired volunteer peers to offer each other intensive, reciprocal, ongoing support via a mobile phone. Low-cost, easy-to-use, mobile phone text-messaging technology added an element of remote support for enhanced reach, effectiveness, and scalability. Two peer mentors with diabetes were identified: positive role models who had lost weight and increased exercise after their T2DM diagnosis. After they were trained in management of diabetes, support processes, and group management by the project team, peer mentors received payment and functioned as peer educators, who conducted a series of 12 drop-in informational support meetings and offered support to the Diabetes Buddy pairs.<br>Weekly sessions were held that included a sequence of identifying weekly successes; learning new information about nutrition, exercise, and disease self-management; problem solving in how to apply the information in daily life; managing uncomfortable emotions such as anger, anxiety, or depression; role-playing new alternative strategies for coping with

	stress; and sharing a meal. In addition, to providing information and support, meetings included self-check of basic diabetes markers (weight, waist circumference, blood pressure). For on-time arrival, women were given pedometers to self-monitor their number of steps daily
**WHO PROVIDED**<br>For each category of intervention provider (e.g., psychologist, nursing assistant), describe their expertise, background and any specific training given.	peer mentors with diabetes were identified: positive role models who had lost weight and increased exercise after their T2DM diagnosis. They were trained in management of diabetes, support processes, and group management by the project team
**HOW**<br>Describe the modes of delivery (e.g., face-to-face or by some other mechanism, such as internet or telephone) of the intervention and whether it was provided individually or in a group.	Face to face group sessions and mobile phone text messaging
**WHERE**<br>Describe the type(s) of location(s) where the intervention occurred, including any necessary infrastructure or relevant features.	Cape Town, South Africa
**WHEN and HOW MUCH**<br>Describe the number of times the intervention was delivered and over what period of time including the number of sessions, their schedule, and their duration, intensity or dose.	12 group sessions (1x per week)<br>daily text messaging<br>12 weeks in duration
**TAILORING**<br>If an intervention was planned to be personalised, titrated or adapted, then describe what, why, when, and how.	Planned personalized with daily texting and support
**MODIFICATION**<br>If an intervention was modified during the course of the study, describe the changes (what, why, when, and how).	N/R
**HOW WELL**<br>Planned: If intervention adherence or fidelity was assessed, describe how and by whom, and if any strategies were used to maintain or improve fidelity, describe them.	N/R
Actual: If intervention adherence or fidelity was assessed, describe the extent to which the intervention was delivered as planned.	N/R

Sazlina, 2015		
**TIDiER Tool Item**	**Main Paper**	**Other Paper(s)**
**BRIEF NAME**<br>Provide the name or a phrase that describes the intervention.	Personalized feedback alone or combined with peer support to improve physical activity in type 2 diabetics	
**WHY**<br>Describe any rationale, theory, or goal of the elements essential to the intervention.	We evaluated the effectiveness of personalized feedback (PF) about physical activity patterns alone or in combination with PS, in addition to usual diabetes care in improving physical activity levels in sedentary older Malays with T2DM. We also evaluated the effectiveness of these interventions on glycosylated hemoglobin, other cardiovascular risk factors, functional status, quality of life, and psychosocial wellbeing.<br><br>The interventions incorporated constructs of Social Cognitive Theory to promote change in behavior from sedentary behavior to being physically active through social support and self-efficacy	
**WHAT**<br>Materials: Describe any physical or informational materials used in the intervention, including those provided to participants or used in intervention delivery or in training of intervention providers.<br>Provide information on where the materials can be accessed (e.g., online appendix, URL).	N/R	
Procedures: Describe each of the procedures, activities, and/or processes used in the intervention, including any enabling or support activities.	PF and PS groups engaged in a 12-week regular unsupervised walking activity. The participants performed gradual walking activity toward the recommended 30 min a day on ≥5 days in a week at moderate intensity and monitored their walking activity intensity using the Talk Test<br><br>Participants in the PF and PS groups received structured PF and usual diabetes care. The feedback comprised participants' physical activity patterns (based on the calculated minutes spent walking in a week each month) provided in three one-to-one sessions with the first author during monthly clinic visits. Their attending doctors at the clinic provided the usual diabetes care.	

	The participants in the PS group received support from peer mentors in addition to the PF and usual diabetes care. They motivated and provided support to the participants to walk regularly based on the feedback through three face-to-face and three telephone contacts over the 12 weeks.<br><br>Participants in the control group received usual diabetes care and acted as a comparison group. The usual diabetes care practice in this study was based on the Malaysian guideline on the management of T2DM, which includes education on lifestyle modification (including diet and physical activity), medications, and self-care management (37). During the 12-week intervention, the control group attended the clinic at a monthly interval to refill their prescriptions. All participants in this study were given pedometers to objectively measure physical activity levels, not as a motivating tool. The motivating factor for the intervention groups was to achieve the recommended duration and frequency of the walking activity. The pedometer readings were not assessed during the 12 weeks of intervention.	
**WHO PROVIDED**<br>For each category of intervention provider (e.g., psychologist, nursing assistant), describe their expertise, background and any specific training given.	Trained Peer mentors were volunteers aged ≥60 years with T2DM who lived in the same community as the participants. PF provided by the first author. Attending doctor provided usual care	Trial protocol page 4<br>A peer mentor is a volunteer with ≥5 years of T2DM, engaged in regular physical activity, has glycosylated haemoglobin level (HbA1c) <8% and living in the community of the study location. Other criteria for a peer mentor include owning a mobile telephone, being willing to attend a 2-day training and complying with the study protocol.
**HOW**<br>Describe the modes of delivery (e.g., face-to-face or by some other mechanism, such as internet or telephone) of the intervention and whether it was provided individually or in a group.	Face to face and telephone, individual	
**WHERE**<br>Describe the type(s) of location(s) where the	N/R	

intervention occurred, including any necessary infrastructure or relevant features.		
**WHEN and HOW MUCH** Describe the number of times the intervention was delivered and over what period of time including the number of sessions, their schedule, and their duration, intensity or dose.	2 day training for peer mentors<br>PF and PS group received 12 weeks of walking gradually to 30 min a day on ≥5 days in a week<br>PF and PS group received feedback through three face-to-face and three telephone contacts over the 12 weeks and three one-to-one sessions.	
**TAILORING** If an intervention was planned to be personalised, titrated or adapted, then describe what, why, when, and how.	Designed to be personalized<br>“personalised feedback” intervention groups	
**MODIFICATION** If an intervention was modified during the course of the study, describe the changes (what, why, when, and how).	N/R	
**HOW WELL** Planned: If intervention adherence or fidelity was assessed, describe how and by whom, and if any strategies were used to maintain or improve fidelity, describe them.	N/R	
Actual: If intervention adherence or fidelity was assessed, describe the extent to which the intervention was delivered as planned.	N/R	

Shahsavari, 2021	
**TIDiER Tool Item**	**Main Paper**
**BRIEF NAME**<br>Provide the name or a phrase that describes the intervention.	Peer support among type 2 diabetic patients
**WHY**<br>Describe any rationale, theory, or goal of the elements essential to the intervention.	This study aimed to investigate the effect of peer support on the QOL among type 2 diabetic patients in deprived areas.
**WHAT**<br>Materials: Describe any physical or informational materials used in the intervention, including those provided to participants or used in intervention delivery or in training of intervention providers. Provide information on where the materials can be accessed (e.g., online appendix, URL).	Education materials for peer supporters
Procedures: Describe each of the procedures, activities, and/or processes used in the intervention, including any enabling or support activities.	To homogenize the participants' basic information, all of them initially participated in a 3-day diabetes self-care education. This education course was conducted by nutritionists and endocrinologists in the clinic. The content of the sessions was prepared according to the 2018 American Diabetes Association Standards of Care and the current instructions of the Ministry of Health of Iran and was implemented after simplifying (Define medical terminology in a language understandable to ordinary people) the concepts. The content included the principles of diabetes self-care (nutrition, physical activity, medication, foot care, and blood sugar control) presented in three 2-h sessions.<br>At the end of the 3-day education course, the research team selected 26 patients with diabetes as potential peers based on the inclusion criteria. The recruited peers participated in four weekly education sessions for 1 month. Each session consisted of 2 h of theoretical education and 1 h of practical education. Regarding the number of peer training sessions, although the references mentioned holding three 2 hour sessions, due to the little information of the selected peers about how to implement the peer support method, the number of sessions increased to 4 sessions. At the end of the education course, which was held by two members of the research team who had worked as a diabetes nurse, the peers received a summary of the educational materials, the schedule and content of the support program prepared for the patients, and a SIM card to communicate with the patients and the research team. During the intervention, the control and intervention groups had no contact with each other.<br><br>The peer support program was conducted within 3 months. During this period, a 2-h education session was held in public places (mosque, coffee shop, and restaurant) per month. The content of the education included the principles of diabetes self-care. In the

	education sessions, the members of the group, while examining the barrier to and facilitators of implementing self-care behaviors, shared their experiences, discussed them, and provided solutions. The peers also arranged a 1-h session, group exercise, and a 2-h group food shopping program for the patients per month. In addition, the peers monitored the patients' care and supported them over the telephone. The duration of telephone calls was 15–20 min once a week. In all the sessions, the peers followed the predetermined topics.<br>They also submitted written details of sessions and telephone conversations to the research team. After reviewing peers' reports, the research team provided them with the necessary feedback to improve the quality of the sessions. During the study, the research team was in contact with the peers by phone. In addition to the 3-day self-care education, the patients in both groups received the routine clinic care, including monthly visits by a diabetes nurse and a nutritionist.
**WHO PROVIDED**<br>For each category of intervention provider (e.g., psychologist, nursing assistant), describe their expertise, background and any specific training given.	Trained peer supporters diagnosed with T2D for at least one year, having at least a high school diploma, having basic knowledge about diabetes (participation in the 3-day education), having no chronic complications of diabetes as discerned by a physician, following their treatment plan (based on the documents on their diabetes record and HbA1C of <8%, having good social, being familiar with the characteristics of the people in the area, attending all peer education sessions, and being approved for their communication and interpersonal skills in the face-to-face interview session by the research team.<br><br>3 day education course conducted by nutritionist and endocrinologist<br><br>Two members of the research team who had worked as a diabetes nurse providing education sessions to peer supporters<br><br>Routine clinic care by a diabetes nurses and nutritionist received by participants in intervention
**HOW**<br>Describe the modes of delivery (e.g., face-to-face or by some other mechanism, such as internet or telephone) of the intervention and whether it was provided individually or in a group.	Face to face, group<br>Telephone, individual
**WHERE**<br>Describe the type(s) of location(s) where the intervention occurred, including any necessary infrastructure or relevant features.	education session was held in public places (mosque, coffee shop, and restaurant)
**WHEN and HOW MUCH**<br>Describe the number of times the intervention was	3 day education course in 2 hour sessions<br>3 hour weekly education sessions for 1 month

delivered and over what period of time including the number of sessions, their schedule, and their duration, intensity or dose.	Within 3 months, 2 hour education sessions, 1 hour group exercises and 2 hour group food shopping<br>15-20 minute weekly phone calls<br>Study duration 3 months
**TAILORING**<br>If an intervention was planned to be personalised, titrated or adapted, then describe what, why, when, and how.	N/R
**MODIFICATION**<br>If an intervention was modified during the course of the study, describe the changes (what, why, when, and how).	Regarding the number of peer training sessions, although the references mentioned holding three 2 hour sessions, due to the little information of the selected peers about how to implement the peer support method, the number of sessions increased to 4 sessions
**HOW WELL**<br>Planned: If intervention adherence or fidelity was assessed, describe how and by whom, and if any strategies were used to maintain or improve fidelity, describe them.	N/R
Actual: If intervention adherence or fidelity was assessed, describe the extent to which the intervention was delivered as planned.	N/R

Sreedevi, 2017	
**TIDiER Tool Item**	**Main Paper**
**BRIEF NAME**<br>Provide the name or a phrase that describes the intervention.	Yoga and peer support on glycaemic outcomes in women with type 2 diabetes mellitus
**WHY**<br>Describe any rationale, theory, or goal of the elements essential to the intervention.	This study aimed at studying the effect of yoga and peer support on glycaemic outcomes, pharmacological adherence and anthropometric measures.
**WHAT**<br>Materials: Describe any physical or informational materials used in the intervention, including those provided to participants or used in intervention delivery or in training of intervention providers. Provide information on where the materials can be accessed (e.g., online appendix, URL).	Participant diaries
Procedures: Describe each of the procedures, activities, and/or processes used in the intervention, including any enabling or support activities.	Instructor driven yoga sessions were conducted for 60 min on two days a week. On the other days the women were instructed to practice at home and maintain a daily log. A record of the food eaten, drugs consumed and exercise particulars were also maintained for two days a week considered to be representative of the entire week. This was reviewed every month.<br>The peer mentors provided support to the study participants in a ratio of 1:14. Peer support meetings: Each peer mentor would visit 13–14 women with diabetes. A face to face meeting with the woman with diabetes in a week for about 45–60 min on assistance in applying disease management or prevention plans in daily life, providing emotional and social support and pro active flexible ongoing support.<br>This was followed up by a telephone call in the same week. A monthly review of the activities was also undertaken by the principal investigator. During the first visit, the peer mentor collected the treatment details including drugs, diet and physical activity. In the follow up visits the peer mentor advised and monitored the woman regarding diet, exercise, timely consumption of drugs, emotional stress, symptoms, foot care etc. In the third month during the last visit the peer mentor conducted a final assessment regarding the entire process, its acceptability, difficulties and usefulness to the woman with diabetes. The woman with diabetes in the peer support group was also given a diary to record the visit, advice of the peer mentor and the changes brought about. The control group was given the usual standard of care including continuing oral hypoglycaemic drugs, advise on diabetic diet and exercise for at least 10 min a day to a level of 150 min/week. All the patients were reviewed monthly and necessary care given.
**WHO PROVIDED**<br>For each category of intervention provider (e.g., psychologist, nursing assistant), describe their	Yoga sessions were conducted by the yoga instructor who had a diploma in yoga and Naturopathy and was assisted by two trained persons with masters in medico-

expertise, background and any specific training given.	social work (MSW).<br>Three Peer mentors were identified from the community and trained. The criteria for eligibility was; having had type two Diabetes for at least one year with a RPG ≤250 mg/dl in the last reading, judged by the investigation team to be generally adherent to treatment and behaviour change regime, capacity and commitment to undergo the training required, an understanding of patients confidentiality, undertaking to liaise with the concerned doctor if unanticipated problems arose during the course of their peer support activity.<br>Peer Mentors underwent a two day training programme consisting of a physician who explained aetiology of diabetes, changes taking place in the body due to Diabetes, complications due to poor glycaemic control and an outline on the drugs used and its mechanism of action and the synergies with physical activity. The nutrition specialist explained all the nutritional and dietary aspects of diabetes; psychologist trained the peer mentors on communication skills, empathy, confidentiality. A training manual was prepared for the peer mentors based on the peers for progress handbook and handed over to the mentors for future reference.
**HOW**<br>Describe the modes of delivery (e.g., face-to-face or by some other mechanism, such as internet or telephone) of the intervention and whether it was provided individually or in a group.	Face-to-face, group
**WHERE**<br>Describe the type(s) of location(s) where the intervention occurred, including any necessary infrastructure or relevant features.	N/R
**WHEN and HOW MUCH**<br>Describe the number of times the intervention was delivered and over what period of time including the number of sessions, their schedule, and their duration, intensity or dose.	Yoga 60 min 2x per week<br>Peer group meetings: once per week face to face for 45-60 min<br>Telephone call once per week<br>12 weeks total
**TAILORING**<br>If an intervention was planned to be personalised, titrated or adapted, then describe what, why, when, and how.	N/R
**MODIFICATION**<br>If an intervention was modified during the course of the study, describe the changes (what, why, when, and how).	N/R
**HOW WELL**<br>Planned: If intervention adherence or fidelity was assessed, describe how and by whom, and if any strategies were used to maintain or improve fidelity, describe them.	N/R
Actual: If intervention adherence or fidelity was	N/R

assessed, describe the extent to which the intervention was delivered as planned.	

Taniguchi, 2018	
**TIDiER Tool Item**	**Main Paper**
**BRIEF NAME**<br>Provide the name or a phrase that describes the intervention.	MoPoTsyo
**WHY**<br>Describe any rationale, theory, or goal of the elements essential to the intervention.	In 2004, MoPoTsyo Patient Information Centre (MoPoTsyo), a Cambodian non-governmental organization, was established to help address this gap in care. MoPoTsyo initially focused on improving access to education and screening through a peer educator network model, then gradually expanded to involve the management of care and treatment. The purpose of this study is to further describe<br>MoPoTsyo's diabetes program in Takeo Province, Cambodia by assessing glycemic and blood pressure (BP) outcomes over 2 years of follow-up.<br>*Related to Hernandez 2021 and Rao 2020
**WHAT**<br>Materials: Describe any physical or informational materials used in the intervention, including those provided to participants or used in intervention delivery or in training of intervention providers. Provide information on where the materials can be accessed (e.g., online appendix, URL).	N/R
Procedures: Describe each of the procedures, activities, and/or processes used in the intervention, including any enabling or support activities.	A retrospective cohort study<br>MoPoTsyo is a Cambodian non-governmental organization based in Phnom Penh, Cambodia.<br>It provides care for adults with diabetes and hypertension through community-based peer educators and access to local medical outpatient consultation, laboratory testing, and a revolving drug fund.<br>A training manual in Khmer was created by MoPoTsyo to educate peer educators about diabetes and hypertension. The diabetes manual informed peer educators about the basic biology of diabetes, medication management, nutrition, and other lifestyle modifications. Candidate peer educators received a total of 6 weeks of training prior to testing needed to become a program peer educator. The first two weeks of training took place in Phnom Penh and included practical training by experienced urban peer educators and theoretical training by medical students hired by MoPoTsyo. The candidate peer educators then received two weeks of field training in screening for diabetes and counseling. The last two weeks allowed for time to revisit and review the knowledge and skills to prepare for a pre-exam. Once they passed the program's pre-exam, they were offered the final exam. Those who passed the pre-exam but failed the final exam had an opportunity to practice and work as a peer educator supervised by a more experienced peer educator until they were ready to retake the final exam

	Peer educators screened travelled from house-to-house in each village screening for the presence of diabetes. To screen for diabetes, MoPoTsyo used urine glucose test strips.<br>After enrollment, the peer educators facilitated patient self-management of their chronic disease to improve glycemic and BP control. In order to accomplish this, peer educators met with patients individually or in a group setting monthly for the first year in the program to provide ongoing diabetes education, support, and to check FBG levels and BP. In addition, patients were encouraged to use urine glucose strips every two weeks to self-monitor their diabetes control. MoPoTsyo encouraged peer educators to create group meetings.
**WHO PROVIDED**<br>For each category of intervention provider (e.g., psychologist, nursing assistant), describe their expertise, background and any specific training given.	MoPoTsyo's candidate peer educators were selected from persons with diabetes based on their ability to read and write and their willingness to commit to fulfill the role. Two thirds were male and levels of education varied widely.
**HOW**<br>Describe the modes of delivery (e.g., face-to-face or by some other mechanism, such as internet or telephone) of the intervention and whether it was provided individually or in a group.	Face to face individual and in groups
**WHERE**<br>Describe the type(s) of location(s) where the intervention occurred, including any necessary infrastructure or relevant features.	Takeo, Cambodia<br>Publicly provided health care in Takeo was divided over 5 operational districts, each one with its own health authority, a referral hospital and health centers typically without physicians. During the period 2007–2013, there was not yet a role for health centers in chronic care provision.
**WHEN and HOW MUCH**<br>Describe the number of times the intervention was delivered and over what period of time including the number of sessions, their schedule, and their duration, intensity or dose.	Monthly meetings (individual or groups) for first year; varied length of visit
**TAILORING**<br>If an intervention was planned to be personalised, titrated or adapted, then describe what, why, when, and how.	Designed to be tailored at group and individual level
**MODIFICATION**<br>If an intervention was modified during the course of the study, describe the changes (what, why, when, and how).	N/R
**HOW WELL**<br>Planned: If intervention adherence or fidelity was assessed, describe how and by whom, and if any strategies were used to maintain or improve fidelity, describe them.	N/R
Actual: If intervention adherence or fidelity was assessed, describe the extent to which the intervention was delivered as planned.	N/R

Thuita, 2020	
**TIDiER Tool Item**	**Main Paper**
**BRIEF NAME**<br>Provide the name or a phrase that describes the intervention.	Nutrition education with and without peer support
**WHY**<br>Describe any rationale, theory, or goal of the elements essential to the intervention.	The purpose of the present study, was to implement a nutrition education (NE) programme with peer to peer support, and evaluate its effect on the MetS and MetS risk factors in adults with Type 2 Diabetes mellitus.
**WHAT**<br>Materials: Describe any physical or informational materials used in the intervention, including those provided to participants or used in intervention delivery or in training of intervention providers. Provide information on where the materials can be accessed (e.g., online appendix, URL).	All study participants received standard education that covered content on diabetes pathophysiology, risk factors, symptoms, complications, hyperglycemia and hypoglycemia symptoms and foot care treatment goals and modalities.<br>Nutrition education program, physical activity lesson, and peer-to-peer training.
Procedures: Describe each of the procedures, activities, and/or processes used in the intervention, including any enabling or support activities.	The study consisted of two intervention groups and a control group. The Nutrition Education (NE) group received nutrition education; the Nutrition Education with Peer-to Peer support (NEP) group received nutrition education with peer-to-peer support; while the control group (C) received standard care. After the standard education, the intervention groups (NE and NEP) underwent a nutrition education programme for 8 weeks, which also covered the importance of physical activity (NE group). In addition, the NEP group was trained on peer-to-peer support. The nutrition education given to the NE and NEP intervention groups included weekly (120 min each) nutrition classes conducted over 8 weeks by the PI. The nutrition education curriculum was developed by the PI after review of related literature on nutrition management of Type 2 diabetes mellitus. The PI also applied her experience gained from practice as a nutritionist. The physical activity lesson was given to the intervention groups (NE and NEP group) in the last week of the education programme. The aim of the physical activity was to ensure that patients accumulate a minimum of 150 min of moderate intensity exercise each week from personal activity at home that includes walking, digging, jogging, cycling, house hold duty, aerobics and sport activities. Participants in the NEP group were divided into small support groups (5–10 participants); depending on the location they came from as well as their age. After each education session, members of the support groups were encouraged to set and share with one another other weekly goals for specific changes in their eating and physical activity behavior. The goals were aimed at making healthy food choices, reduction of portion sizes and being active. The participants reported on their progress to the group members at the beginning of the next session. After the 8 week training, participants were followed monthly, and they

	presented their progress and new goals to the group members, for a period of 6 months. A trained peer educator living with diabetes for 13 years from Kenya Defeat Diabetes Association (KDDA) joined the PI during the monthly meetings and encouraged the participants in the peer support groups by sharing his experiences. Together with the PI he also assisted them review and adjust their goals during monthly meetings. Also, group counseling was done on each visit for participants requiring more support.
**WHO PROVIDED**<br>For each category of intervention provider (e.g., psychologist, nursing assistant), describe their expertise, background and any specific training given.	Principal investigator (PI) together with a clinician who runs the clinic (Registered Clinical Officer with a Bachelor of Science degree in Clinical medicine). trained peer educator living with diabetes for 13 years from Kenya Defeat Diabetes Association.
**HOW**<br>Describe the modes of delivery (e.g., face-to-face or by some other mechanism, such as internet or telephone) of the intervention and whether it was provided individually or in a group.	Face to face, group
**WHERE**<br>Describe the type(s) of location(s) where the intervention occurred, including any necessary infrastructure or relevant features.	The study was conducted at Thika Level 5 Hospital (TL5H) in Kiambu County, Kenya at the Diabetes Comprehensive Care Centre (DCC). The clinic attends to approximately one hundred patients per week. The DCC is an outpatient clinic that operates on a daily basis. Diabetic patients from Kiambu County and nearby areas attend the clinic on appointment days for routine monitoring of blood glucose, blood pressure and nutrition status (body mass index; BMI), as well as for treatment and collection of medication. The clinic serves both male and female patients with Type 1 and Type 2 diabetes mellitus. The patients are mainly from low and middle income backgrounds.
**WHEN and HOW MUCH**<br>Describe the number of times the intervention was delivered and over what period of time including the number of sessions, their schedule, and their duration, intensity or dose.	The nutrition education programme was conducted for 2 h per week for 8 weeks. In addition, the NEP had weekly peerto-peer interactions for 8 weeks. All groups had follow-up sessions for 6 months. The follow up was done monthly after the intervention period. After the end of the 8 weeks intervention the patient were requested to be coming to the hospital monthly on selected days for follow up. Patient in the NEP group continued with peer to peer support during the follow up period.
**TAILORING**<br>If an intervention was planned to be personalised, titrated or adapted, then describe what, why, when, and how.	Planned to be personalized
**MODIFICATION**<br>If an intervention was modified during the course of the study, describe the changes (what, why, when, and how).	N/R
**HOW WELL**<br>Planned: If intervention adherence or fidelity was	N/R

assessed, describe how and by whom, and if any strategies were used to maintain or improve fidelity, describe them.	
Actual: If intervention adherence or fidelity was assessed, describe the extent to which the intervention was delivered as planned.	N/R

Yin, 2015	
**TIDiER Tool Item**	**Main Paper**
**BRIEF NAME**<br>Provide the name or a phrase that describes the intervention.	Peer Support in People With Type 2 Diabetes
**WHY**<br>Describe any rationale, theory, or goal of the elements essential to the intervention.	We examined the effects of participating in a “train-the-trainer” program and being a peer supporter on metabolic and cognitive/psychological/ behavioral parameters in Chinese patients with type 2 diabetes.<br>*Related to Chan et al 2014
**WHAT**<br>Materials: Describe any physical or informational materials used in the intervention, including those provided to participants or used in intervention delivery or in training of intervention providers. Provide information on where the materials can be accessed (e.g., online appendix, URL).	Peer supporters were given a checklist
Procedures: Describe each of the procedures, activities, and/or processes used in the intervention, including any enabling or support activities.	The train-the-trainer program was designed to empower trainees to provide basic knowledge and emotional support to their peers with type 2 diabetes. The program consisted of 4 monthly workshops, each lasting 8 hours, for a total of 32 hours. Health care experts led the workshops, which included both didactic components and interactive components such as role playing and group sharing.<br><br>Each agreed trainee was assigned 10 patients of the same gender to support. Agreed trainees were introduced to their patient groups in several meetings where the rationale, purpose, and expectations for this study were explained. The meeting was hosted by 1 attending doctor, 1 nurse, and the project coordinator. The peer supporters were asked to provide structured peer support for at least 1 year, with provision for a voluntary extension of 3 more years.<br>We have described elsewhere how peer support was delivered during the 1-year structured program. Briefly, the peer supporters were asked to give each of their assigned patients a 15- to 20-minute telephone call biweekly for the first 3 months, monthly for the second 3 months, and every 2 months for the last 6 months. Peer supporters were given a checklist to use in reviewing specific self-management skills, including medication adherence, healthy diet, regular exercise, sick day management, foot care, and glucose monitoring. They were also encouraged to provide psychological support based on their own experiences. Peer supporters submitted their phone call checklists every 3 months for documentation of their discussion items, duration of each call, and relevant remarks. Additional electronic communication and group gatherings were left to the discretion of the participants. During the voluntary extension period, the

	peer supporters were asked to maintain contact with their assigned patients every 1 to 2 months for another 3 years. They were also required to document the calls and return the checklists to the project coordinator every year
**WHO PROVIDED**<br>For each category of intervention provider (e.g., psychologist, nursing assistant), describe their expertise, background and any specific training given.	Trained peer supporters with type 2 diabetes aged 18 to 75 years with fair glycemic control (HbA1c <8%), good understanding of living with diabetes, clear communication skills, and a desire to serve were invited to attend a “train-the-trainer” program. Exclusion criteria included illiteracy, physical impairment, and mental illness impairing communication with others.
**HOW**<br>Describe the modes of delivery (e.g., face-to-face or by some other mechanism, such as internet or telephone) of the intervention and whether it was provided individually or in a group.	Telephone, individual
**WHERE**<br>Describe the type(s) of location(s) where the intervention occurred, including any necessary infrastructure or relevant features.	N/A
**WHEN and HOW MUCH**<br>Describe the number of times the intervention was delivered and over what period of time including the number of sessions, their schedule, and their duration, intensity or dose.	15-20 minute telephone calls biweekly for the first 3 months, monthly for the second 3 months, and every 2 months for the last 6 months (1 year)<br>During extension period, maintain contact every 1 to 2 months for another 3 years
**TAILORING**<br>If an intervention was planned to be personalised, titrated or adapted, then describe what, why, when, and how.	Designed to be personalised (behavioural psychology – goal setting)
**MODIFICATION**<br>If an intervention was modified during the course of the study, describe the changes (what, why, when, and how).	N/R
**HOW WELL**<br>Planned: If intervention adherence or fidelity was assessed, describe how and by whom, and if any strategies were used to maintain or improve fidelity, describe them.	N/R
Actual: If intervention adherence or fidelity was assessed, describe the extent to which the intervention was delivered as planned.	N/R

Zeng, 2016	
**TIDiER Tool Item**	**Main Paper**
**BRIEF NAME**<br>Provide the name or a phrase that describes the intervention.	Community-based controlled trial of a comprehensive psychological intervention
**WHY**<br>Describe any rationale, theory, or goal of the elements essential to the intervention.	Community-based health services in China do not have the resources or personnel needed to provide sophisticated, individual based psychopharmacological or psychotherapeutic services to these individuals, so we decided to adapt the multi-faceted 'Collaborative Care Model,' originally developed in the United States, for use in Shanghai. This care-delivery model is targeted at all patients with hypertension or diabetes, regardless of the severity of their psychological symptoms. It aims to improve service quality by creating community-based health care teams that integrate routine surveillance and positive follow up of patients' medical condition with assessment of their psychological status, and, if necessary, provision of social support to help the individual and his/her family members adjust to their stressful life circumstances. The current study uses a community-based design to assess the effectiveness of this comprehensive approach to improve the psychological health, physical health, and quality of life of individuals with diabetes or hypertension.
**WHAT**<br>Materials: Describe any physical or informational materials used in the intervention, including those provided to participants or used in intervention delivery or in training of intervention providers. Provide information on where the materials can be accessed (e.g., online appendix, URL).	The community-based mental health education component involved distributing brochures, broadcasting educational videos, and hosting lectures about psychosomatic health for individuals with chronic illnesses. The content focused on the identification and management of the symptoms of depression and anxiety, the relationship between psychological health and somatic health, and the relationship between stress and depression or anxiety.
Procedures: Describe each of the procedures, activities, and/or processes used in the intervention, including any enabling or support activities.	All participants received routine management of their chronic illness. As described above, in CHCs in Shanghai this is officially supposed to include registration, complete annual physical examinations, and quarterly follow-up of community residents with adult-onset diabetes and primary hypertension.<br>The quarterly follow-up assessments include assessment of blood pressure and fasting blood glucose, identification of sequelae or comorbid health conditions, health education about lifestyle issues, medication management, and, if necessary, referral to hospital outpatient or inpatient services for more extensive evaluation or treatment. The degree to which community residents with diabetes and hypertension participate in these CHC services varies considerably.<br>The community-based comprehensive psychological intervention used in this study was an adaptation of the IMPACT model developed in the United States for use in Shanghai. In addition to the routine management of

	their diabetes and/or hypertension, all intervention group subjects also received community based education about psychological health. Some individuals in the intervention group also received additional psychological support.<br>The peer support group intervention targeted patients with diabetes or hypertension who had PHQ-9 or GAD-7 scores > 5 but also welcomed the participation of other community members who expressed interest in the groups. This intervention involved monthly 60-90 minute meetings led by community volunteers who had received guidance from counselors. The group meetings, which typically included 9-18 individuals, focused on (a) the management of chronic diseases, (b) healthy lifestyles, (c) psychological coping skills for dealing with diabetes and hypertension, (d) knowledge about depression and anxiety, and (e) self-awareness of negative emotions. In addition to the transmission of crucial information, the meetings also provided emotional and social support to the participants, something that previous research has shown to reduce depressive symptoms and improve the control of diabetes and hypertension.<br>The individual intervention targeted individuals whose PHQ-9 or GAD-7 score was >10. Counselors (individuals who had a nationally approved Level-2 counseling certificate) provided one 60-minute and six 30-minute sessions of Problem Solving Treatment for Primary Care (PST-PC) to each individual. The counseling focused on alleviating symptoms of depression and anxiety by assisting these individuals to become more self-aware, to learn how to analyze and deal with their problems, to decrease their feelings of frustration, and to increase their feelings of control over their lives. PST has been found to be effective in the management of emotional problems among patients treated at community health centers.
**WHO PROVIDED**<br>For each category of intervention provider (e.g., psychologist, nursing assistant), describe their expertise, background and any specific training given.	Each service team typically includes a general doctor, a nurse, and a public health clinician; among other responsibilities, they are expected to establish and maintain a registry of all residents with hypertension or diabetes in the neighborhoods; assess their blood pressure, blood sugar, and medication adherence at least four times a year; provide a full medical exam annually; refer those who need more advanced treatment; and provide related health education. The three components of this community-based intervention in the 34 neighborhoods was collaboratively coordinated and provided by 391 individuals, including local administrators, community clinicians, community public health workers, counselors, and volunteers. All individuals who provided each of the three components of the

	intervention received appropriate training before implementing the intervention.
**HOW**<br>Describe the modes of delivery (e.g., face-to-face or by some other mechanism, such as internet or telephone) of the intervention and whether it was provided individually or in a group.	Face to face, individual and group
**WHERE**<br>Describe the type(s) of location(s) where the intervention occurred, including any necessary infrastructure or relevant features.	Community health services in Shanghai are provided by community health centers (CHCs) distributed throughout the municipality's 16 districts. Each community health center has a number of 'community health service teams' responsible for monitoring chronic illnesses among residents of several neighborhoods within the service area covered by the community health center.<br>Study participants were community residents registered with diabetes or hypertension from three CHCs in two of Shanghai's 16 districts (the Xinhua CHC and the Huayang CHC in the Changning District and the Xinzhuang CHC in the Minhang District). As shown in Figure 1, participants came from 62 neighborhoods in the catchment areas of these three CHCs that were provided services by 11 separate community health service teams; all 17 neighborhoods serviced by four community health service teams in the Xinhua CHC; all 21 neighborhoods serviced by four community health service teams in the Huayang CHC; and 24 of the 55 neighborhoods serviced by three of the community health teams in the Xinzhuang CHC.
**WHEN and HOW MUCH**<br>Describe the number of times the intervention was delivered and over what period of time including the number of sessions, their schedule, and their duration, intensity or dose.	standard care, monthly 60-90min peer group meetings, and/or one 60min and six 30min individual sessions<br>6 months
**TAILORING**<br>If an intervention was planned to be personalised, titrated or adapted, then describe what, why, when, and how.	parts were designed to be personalised via groups or individual meetings
**MODIFICATION**<br>If an intervention was modified during the course of the study, describe the changes (what, why, when, and how).	N/R
**HOW WELL**<br>Planned: If intervention adherence or fidelity was assessed, describe how and by whom, and if any strategies were used to maintain or improve fidelity, describe them.	During the intervention process, peer support leaders and the counselors also routinely received professional supervision in order to identify and address any problems in a timely manner.
Actual: If intervention adherence or fidelity was assessed, describe the extent to which the intervention was delivered as planned.	Low participation rate in the small-group peer support effort (31% of eligible individuals participated) and in the PST counseling component of the intervention (9% of eligible individuals participated). Only 349 of the 6897 (5%) individuals in the intervention neighborhoods who completed the baseline assessment participated in

	these components of the intervention

Zhong, 2015	
**TIDiER Tool Item**	**Main Paper**
**BRIEF NAME**<br>Provide the name or a phrase that describes the intervention.	Peer leader–support program (PLSP) for diabetes self-management in China
**WHY**<br>Describe any rationale, theory, or goal of the elements essential to the intervention.	Our research project examined a peer-support intervention for type 2 diabetes in primary care community health services centers (CHSCs) in Anhui Province
**WHAT**<br>Materials: Describe any physical or informational materials used in the intervention, including those provided to participants or used in intervention delivery or in training of intervention providers. Provide information on where the materials can be accessed (e.g., online appendix, URL).	A peer leader handbook included materials for use with participants
Procedures: Describe each of the procedures, activities, and/or processes used in the intervention, including any enabling or support activities.	Nineteen "peer support groups," 1 for each peer leader, were set up in the subcommunities randomized to the PLSP condition. Each group consisted of 10-15 participants. The protocol called for twelve biweekly education meetings over 6 months to be co-led by peer leaders with CHSC staff involvement titrated to peer leaders' needs. Meetings lasted 1.5 to 2 hours and covered a range of topics such as diet, physical activity, medications, foot care, stress management and depression, barriers to self-management, and obtaining resources and support from the community, family, friends, and the health system. For efficiency, groups were often combined, resulting in meetings of more than 30 participants and consequently limited opportunity for discussion. Accordingly, the protocol also called for peer leaders to lead 12 biweekly discussion meetings over 6 months. These reviewed the topics of the education meetings and included sharing experiences and modeling self-management practices. Peer leaders also led or encouraged informal activities (for instance, walking and tai chi groups) among group members. Because peer leaders and participants lived within the same subcommunities, casual interactions and activities were common.
**WHO PROVIDED**<br>For each category of intervention provider (e.g., psychologist, nursing assistant), describe their expertise, background and any specific training given.	CHSC staff recruited 19 peer leaders who had been diagnosed with type 2 diabetes for more than 1 year, were willing to volunteer, and generally adhered to both medication and behavioral management regimens. Additional criteria were altruism, positive and sociable personality, availability of time, an understanding of the importance of patient confidentiality, good relationships with community residents, and leadership in their communities. Further selection was based on willingness to liaise with CHSC staff in response to unanticipated problems, to commit to the project schedule, to take on the responsibilities of peer leaders and adhere to program policies, to attend 3 days'

	training, and to contact group members frequently. Peer leaders were retired adults who had had diabetes for a mean of 9.3 years. Although some were non-professionals, a number had work experience in teaching, nursing, or the like. Sixteen of 19 were male (84.2%). The Anhui CDC research team provided 3 days' training for the peer leaders, including an introduction to the PLSP and training in basic skills and diabetes self-management. Training emphasized the key functions of peer support promoted by Peers for Progress:<br>• Assisting and encouraging daily diabetes management<br>• Providing social and emotional support<br>• Linking with community resources and primary care at the CHSSs<br>• Providing ongoing support
**HOW**<br>Describe the modes of delivery (e.g., face-to-face or by some other mechanism, such as internet or telephone) of the intervention and whether it was provided individually or in a group.	Face to face, group
**WHERE**<br>Describe the type(s) of location(s) where the intervention occurred, including any necessary infrastructure or relevant features.	The Community Chronic Disease Management System provides population-wide primary care through CHSCs and their community health service stations (CHSSs). An important feature of the setting was its integration of primary care with communities. Each community is subdivided into subcommunities, each with its own community-neighborhood committee and each served by its own CHSS. Individuals in a particular housing site receive their care through a clinical team assigned to that site. Where the PLSP is implemented, peer leaders receive care through the same team as those with whom they live.
**WHEN and HOW MUCH**<br>Describe the number of times the intervention was delivered and over what period of time including the number of sessions, their schedule, and their duration, intensity or dose.	twelve biweekly<br>education meetings over 6 months to be co-led by peer leaders with CHSC staff involvement titrated to peer leaders' needs. Meetings lasted 1.5 to 2 hours
**TAILORING**<br>If an intervention was planned to be personalised, titrated or adapted, then describe what, why, when, and how.	N/R
**MODIFICATION**<br>If an intervention was modified during the course of the study, describe the changes (what, why, when, and how).	The formative evaluation indicated substantial support for the PLSP model and for a systematic study of its effectiveness. Therefore, the program was not altered before implementation.
**HOW WELL**<br>Planned: If intervention adherence or fidelity was assessed, describe how and by whom, and if any strategies were used to maintain or improve fidelity, describe them.	Formative evaluation addressed the feasibility, adaptability, and acceptability of the program and its key features relative to community and organization policy. It was also intended to engage and empower local communities to be part of program development. Focus groups were held in 2 communities and individual interviews in all 3 communities. The interviews

	included the leaders of each of the 6 CHSSs, 2 in each community, that had agreed to participate in the study, along with the leader of the district health bureau and the leader of the neighborhood committee associated with each of the 6 CHSSs
Actual: If intervention adherence or fidelity was assessed, describe the extent to which the intervention was delivered as planned.	As detailed in Table 1, key representatives from local Community Neighborhood Committees indicated that PLSP would be acceptable and feasible for their neighborhoods. Responses of CHSC directors, staff, and patients were also positive and included expressions of desire for "more training and direction" along with some concern among staff that "…the program may bring a large work burden for us." Accordingly, health authorities in the 3 cities agreed to provide policy, technical, and modest financial support to the PLSP.<br><br>Implementation in community 3 did not achieve protocol objectives. Only 3 peer leaders were recruited and only 3 peer groups of 10 to 15 participants were organized. As a result, most participants from community 3 did not have the opportunity to attend group meeting and activities.
